# Dual Transcriptomics Reveals Interspecific Interactions between the Mycoparasite *Calcarisporium cordycipiticola* and Its Host Cordyceps militaris

**DOI:** 10.1128/spectrum.04800-22

**Published:** 2023-03-22

**Authors:** Qing Liu, Caihong Dong

**Affiliations:** a State Key Laboratory of Mycology, Institute of Microbiology, Chinese Academy of Sciences, Beijing, China; b University of Chinese Academy of Sciences, Beijing, China; Universita degli Studi del Molise

**Keywords:** *Calcarisporium cordycipiticola*, *Cordyceps militaris*, dual transcriptome, white mildew disease, mycoparasitic strategy, host response, iron, ROS

## Abstract

Calcarisporium cordycipiticola is a mycoparasite of the edible fungus Cordyceps militaris, and mycoparasitism causes devastating diseases of mushrooms. In this study, dual-transcriptomic analysis was performed to reveal interspecific interactions between the mycoparasite C. cordycipiticola and its host C. militaris. At 4 and 8 days postinfection (dpi), 2,959 and 2,077 differentially expressed genes (DEGs) of *C. cordycipiticola* and 914 and 1,548 DEGs of *C. militaris* were identified compared with the mycelial stage, respectively, indicating that *C. cordycipiticola* responded more quickly than *C. militaris*. Lectins of the pathogen may play a role in the recognition of fungal prey. Both Gene Ontology (GO) and Kyoto Encyclopedia of Genes and Genomes (KEGG) analyses showed that primary metabolism was vigorous for the pathogen to colonize the host and that the pathogen’s attack substantially altered *C. militaris*’ primary metabolism. *C. cordycipiticola* upregulated some carbohydrate-active enzyme (CAZyme) genes, including *CBM18*, *GH18*, *GH16*, and *GH76*, for degrading the host cell wall and defending against host immunity. *C. militaris* produced excessive reactive oxygen species (ROS) to respond to the infection. The GO term “heme binding” was the only shared term enriched at both stages at 4 and 8 dpi, indicating that iron was important for both the pathogen and the host. The uptake of iron by pathogens through multiple pathways promoted colonization and removed high ROS levels produced by the host. The transcription levels of *Cmhsp78*, *Cmhsp70*, and *Cmhyd1* in *C. militaris* responded quickly, and these genes have potential as candidates for the breeding of resistant varieties. This study provides clues for understanding the interactions between a mycoparasite and its mushroom host and will be helpful for the breeding of resistant varieties and disease prevention and control for this edible fungus.

**IMPORTANCE** White mildew disease caused by *Calcarisporium cordycipiticola* is devastating for the fruiting body cultivation of Cordyceps militaris, a popular and highly valued edible fungus. Here, the pathogenic mechanisms of *C. cordycipiticola*, the responses of *C. militaris* to the infection, and the interaction of these two phylogenetically close species were revealed by time course dual-transcriptome profiles. In general, the host *C. militaris* responds more slowly than the pathogen *C. cordycipiticola*. For the first time, we found that iron was important for both the mycoparasite and the host. *C. cordycipiticola* takes up iron by multiple pathways to promote colonization and remove high ROS levels produced by the host. The rapidly responding genes *Cmhsp70*, *Cmhsp78*, and *Cmhyd1* in *C. militaris* may have the potential as candidate genes for the breeding of resistant varieties. This study expands our understanding of the mycoparasitic interactions of two species from sister families and will be helpful for the breeding of and disease prevention and control in mushrooms.

## INTRODUCTION

Mycoparasitism is a lifestyle in which one fungus establishes parasitic interactions with other fungi. The fungal host is parasitized by and acts as a source of nutrients for the mycoparasite (1, 2). The best-studied mycoparasitic interactions are those of some species of *Trichoderma* and Clonostachys rosea with their hosts due to the potential for biological control activity against plant-pathogenic species (3, 4).

If the host is a mushroom, mycoparasitic fungi may be pathogenic and, thus, may cause devastating diseases of mushrooms in nature and industry by reducing yields and quality worldwide (5). Reported mycoparasites of mushrooms include Hypomyces perniciosus causing wet bubble disease in Agaricus bisporus (6), Lecanicillium fungicola causing dry bubble disease in A. bisporus (7, 8), Cladobotryum dendroides causing cobweb disease in *A. bisporus* (9), and Paecilomyces penicillatus causing disease in Morchella importuna (10, 11), etc. Some transcriptional analyses were performed during pathogenesis, focusing on the pathogenicity strategy. Protein families of transporters, cell wall-degrading enzymes, cytochrome P450, and secondary metabolites (SMs) are essential for H. perniciosus mycoparasitism to *A. bisporus* and adaptation to harsh environments (6). Genes encoding several oxidoreductases, cell wall-degrading enzymes, and ATP-binding cassette (ABC) and major facilitator superfamily (MFS) transporters as well as other various genes may play roles in the pathosystems of L. fungicola mycoparasitism to *A. bisporus* (8). The expression levels of diphthamide biosynthesis, aldehyde reductase, and NAD(P)H-hydrate epimerase genes were increased during infection of the edible fungus M. importuna by P. penicillatus (10). Except for the shared cell wall-degrading enzymes, it seems that each mycoparasitic fungus has its specific pathogenic strategy.

The outcome of host-parasite interactions in fungal infections is determined by the balance between the pathogenicity of the mycoparasite and the adequacy of the host’s defenses. When plants are infected by a pathogen, resistance responses are induced, including the accumulation and removal of reactive oxygen species (ROS), the generation and transduction of disease resistance signals, the expression and regulation of defense responses, and the synthesis of secondary products (12). How will edible fungi respond when they are infected by pathogens? Genes involved in antioxidant systems, peroxisome biogenesis, autophagy, oxidation-reduction, ribosome biogenesis, and cell wall degradation were found as responses to pathogenic bacteria in Pleurotus eryngii (13). The expression levels of 10 genes, including a cyclin-dependent protein kinase inhibitor and others with unknown functions, in *M. importuna* were increased during infection, and these genes may regulate the response of *M. importuna* to *P. penicillatus* infection (10). The defensive response of mushrooms to mycoparasite action remains broadly unresolved.

Cordyceps militaris (L.) Fr. is an important edible and medicinal fungus, and its annual value has increased year by year, reaching 10 billion renminbi in China in 2016 (14). However, fungal diseases have become one of the bottlenecks in the large-scale production of fruiting bodies, among which white mildew disease caused by Calcarisporium cordycipiticola has been the most destructive and has resulted in huge economic losses to the C. militaris industry in China (15, 16). There has been no effective prevention and control method until now.

**FIG 1:**
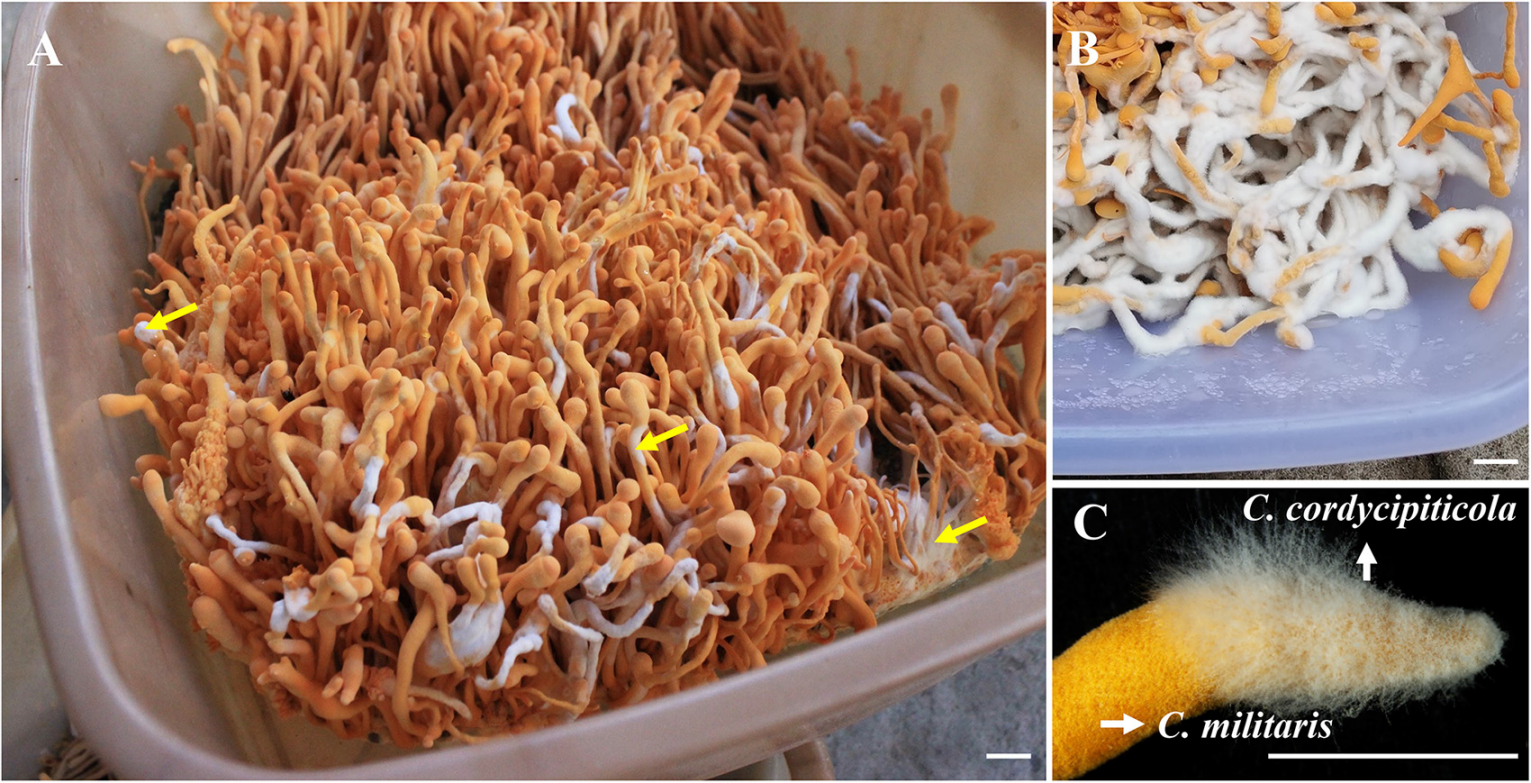
The mycoparasitic fungus *Calcarisporium cordycipiticola* and its host Cordyceps militaris. Diseased fruiting bodies of *C. militaris* are shown. Bars = 1 cm. The yellow arrows indicate different sites of disease on the fruiting bodies.

C. cordycipiticola was confirmed to be the pathogen of white mildew disease of *C. militaris* by Koch’s rules (15). The disease often occurs at the later stages of the growth and development of *C. militaris*, and the mycoparasite can grow on the surfaces of the fruiting bodies and cover them fully (Fig. 1) (15). The hyphae of *C. cordycipiticola* can invade the gaps among hyphae of the fruiting bodies of *C. militaris* and fill them gradually. They can degrade the hyphae of the host by both direct contact and noncontact (16). The parasitism is initially biotrophic and then necrotrophic as the mycoparasitic interaction progresses (16). Beyond that, there had been no information on the pathogenic mechanism of *C. cordycipiticola* (pathogen) and the mechanism of the response of *C. militaris* (host), which is crucial for disease prevention and control.

Dual RNA sequencing (RNA-seq) can simultaneously capture all classes of coding and noncoding transcripts in both the pathogen and the host (17). In this study, time course dual-transcriptome profiles were analyzed to reveal the interactions between the destructive mycoparasite *C. cordycipiticola* and the host *C. militaris*. The genes differentially expressed genes during the infection processes were analyzed for *C. cordycipiticola* and *C. militaris*, and the interactions were then analyzed. The pathogenic mechanisms of *C. cordycipiticola* and the responses of *C. militaris* to the infection were revealed. This study will be helpful for the understanding of the interspecific interactions between this mycoparasite and its host and also for the breeding of resistant varieties of this mushroom.

## RESULTS

### Global transcriptome analysis during infection.

**FIG 2:**
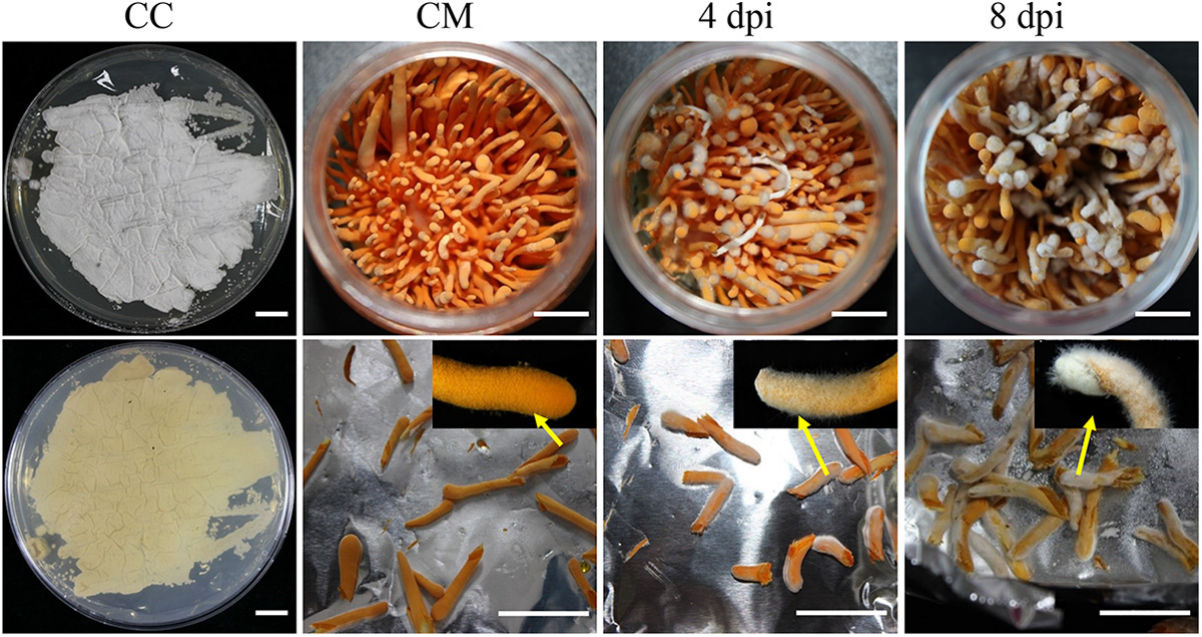
Samples used for the dual-RNA-seq analysis. CC, mycelia of *C. cordycipiticola*; CM, fruiting bodies of *C. militaris*; 4 dpi and 8 dpi, infected fruiting bodies of *C. militaris* at 4 and 8 days postinfection, respectively. Bars = 1 cm. Insets indicate enlarged views of the fruiting bodies.

To characterize the gene expression profiles of *C. militaris* and *C. cordycipiticola* during infection, time course dual-RNA-seq analyses were performed (see Fig. S1 in the supplemental material). The samples were harvested after being infected for 4 and 8 days and named 4 dpi (days postinfection) and 8 dpi, respectively. The mycelia of *C. cordycipiticola* (CC) and the healthy fruiting bodies of *C. militaris* (CM) used for infection served as the controls (Fig. 2).

Illumina paired-end sequencing generated a total of 151.76 Gb of clean data, with averages of 9.45 Gb per pure cultured sample and 15.8 Gb per infected sample (Table S1). All *Q*_30_ percentages for the sequences (with an error probability of 0.01) in the 12 libraries were over 91.50% (Table S1). For the CC sample, 93.17% ± 0.40% of reads were uniquely mapped to the *C. cordycipiticola* reference genome, and only 0.27% ± 0.09% were mapped to that of the host *C. militaris*. Similarly, for the CM sample, 85.27% ± 1.37% of the reads were uniquely mapped to the *C. militaris* reference genome, and 1.34% ± 0.63% were mapped to that of the pathogen *C. cordycipiticola* (Table S1). The mapped data for each sample had genome coverages that were over or close to 100× (Table S1). Although *C. militaris* and *C. cordycipiticola* are phylogenetically closely related, their transcriptome data can be completely separated, which lays the foundation for our interaction transcriptome analysis. Pearson’s correlation coefficients of gene expression between replicates for each sample were >0.86, indicating good repeatability (Fig. S2).

### Differentially expressed genes of *Calcarisporium cordycipiticola* during infection.

**FIG 3:**
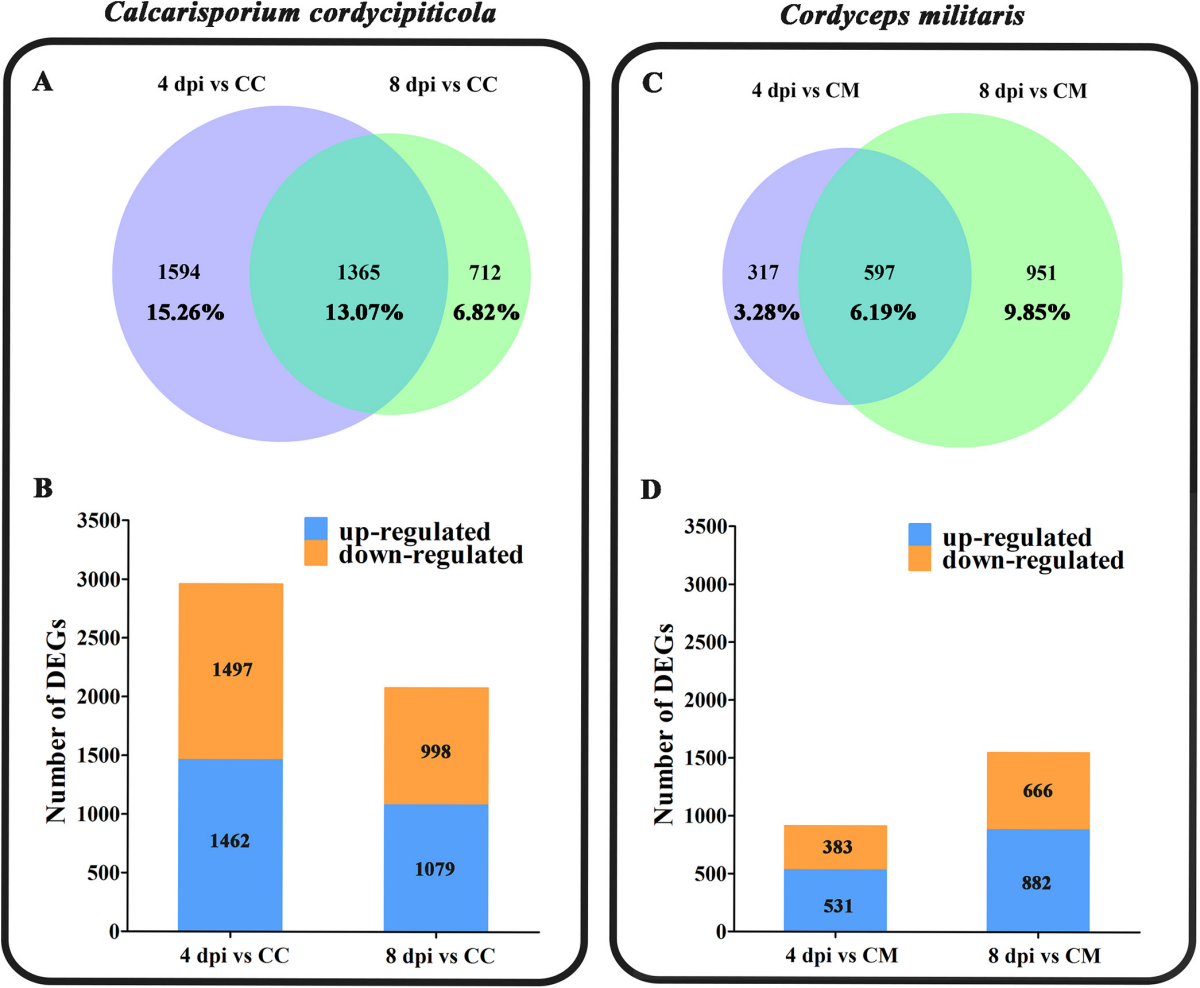
Analysis of DEGs of *Calcarisporium cordycipiticola* and Cordyceps militaris. (A and C) Venn diagrams showing the DEGs of *C. cordycipiticola* (A) and *C. militaris* (C). Percentages are the ratios of the number of DEGs to the total number of genes. (B and D) Up- and downregulated genes of *C. cordycipiticola* (B) and *C. militaris* (D). The numbers of up- and downregulated genes are indicated in the histograms.

**FIG 4:**
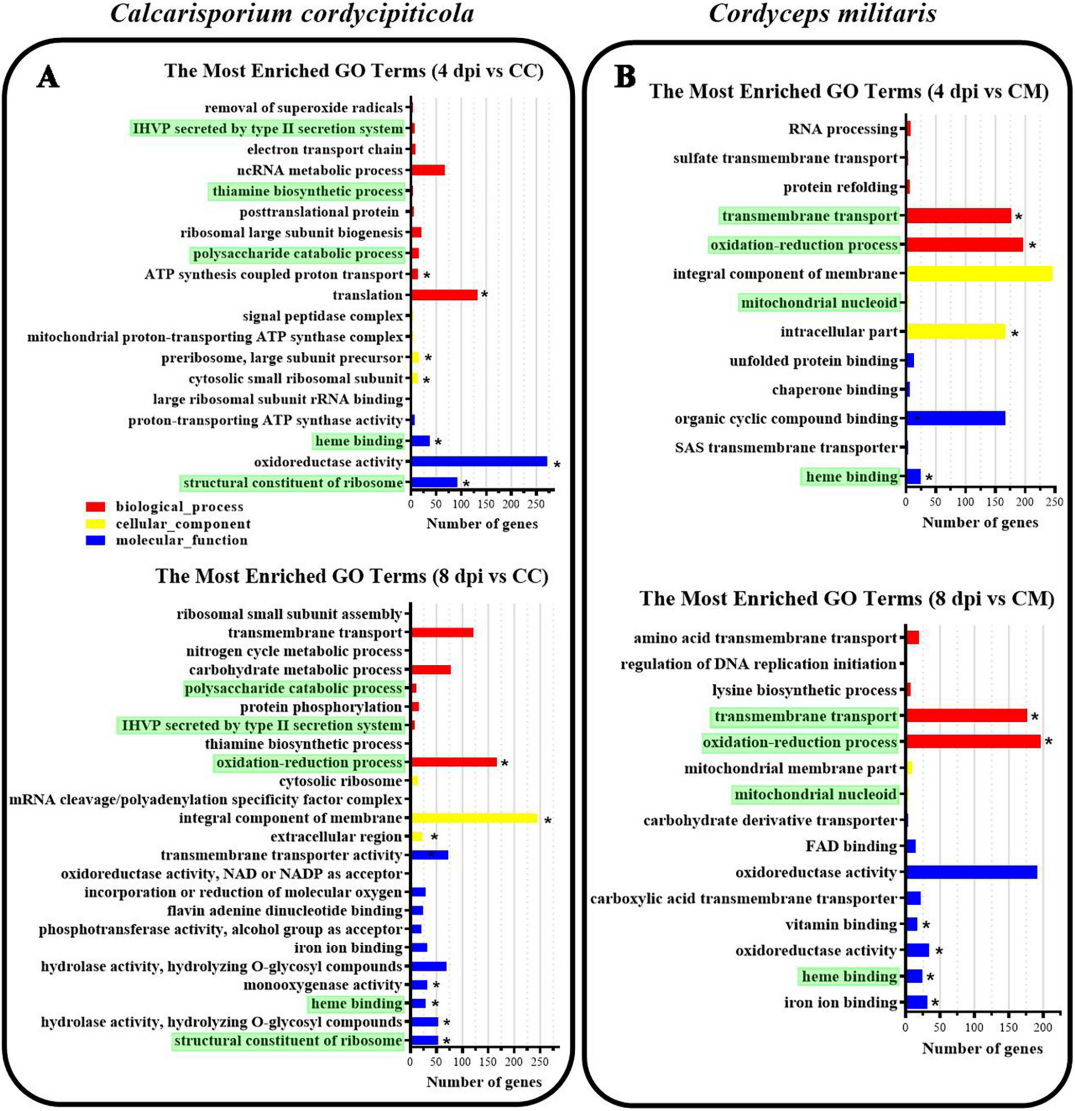
GO functional classification of differentially expressed genes in *Calcarisporium cordycipiticola* (A) and Cordyceps militaris (B). The red, yellow, and blue bars represent biological processes, cellular components, and molecular functions, respectively. Only significant GO terms (*P < *0.01) are shown. Asterisks indicate GO terms with a *P* value of <0.001. Shades of green indicate the shared GO terms at the two stages (4 and 8 dpi). ncRNA, noncoding RNA; FAD, flavin adenine dinucleotide; IHVP, interaction with host via protein; SAS, secondary active sulfate.

For *C. cordycipiticola*, 2,959 and 2,077 differentially expressed genes (DEGs), which were 28.33% and 19.89% of the total genes, were identified at 4 and 8 dpi compared with CC, respectively (Fig. 3A and B), and thus were involved in the interaction. Gene ontology (GO) analysis showed that the DEGs were enriched in 19 and 24 terms at 4 and 8 dpi, respectively (*P < *0.01) (Fig. 4A). Among them, 5 terms were shared, and “structural constituent of ribosome” was the most enriched term at both stages (Fig. 4A). Kyoto Encyclopedia of Genes and Genomes (KEGG) analysis showed that there were 3 and 2 significant pathways at 4 and 8 dpi (corrected *P* value of <0.05), respectively, and the ribosome pathway was the most significant pathway at both stages (Table S2). Both GO and KEGG analyses showed that the ribosome pathway was important, suggesting that protein synthesis was vigorous in the pathogen during infection. The KEGG pathways oxidative phosphorylation and propanoate metabolism were especially enriched (Table S2) at 4 dpi. One KEGG pathway (starch and sucrose metabolism) was especially enriched at 8 dpi (Table S2). It seemed that primary metabolism was vigorous in the pathogen during infection.

Among the top 20 upregulated genes at 4 dpi versus CC, 14 encoded secreted proteins, among which 7 were effectors (Table S3). For the secretome of *C. cordycipiticola*, 52 genes were expressed only during infection (at 4 and 8 dpi), including 6 encoding extracellular metalloproteases and 5 encoding proteases or peptidases (Table S4). Among the expressed genes encoding secreted proteins, 88.40% (381/431) and 87.04% (376/432) were differentially expressed at 4 and 8 dpi compared with CC, respectively (Fig. S3A and Table S4). Among 189 effectors predicted in the genome of *C. cordycipiticola* (16), 89 were differentially expressed at 4 or 8 dpi compared with CC (Table S4), among which 76.4% (68/89) were significantly upregulated (Fig. S3B and Table S4). These DEGs included genes encoding metalloprotease-1, serine proteases or peptidases, and glycoside hydrolases (glycoside hydrolase family 16 [GH16] and GH25), etc. (Table S4), and most of them encoded hypothetical proteins.

The DEG with the highest transcription level at 4 dpi was *CCOR_05591*, encoding a secreted small cysteine-rich protein with a common fungal extracellular membrane (CFEM) domain. It was upregulated 3-fold at 4 dpi, with an average fragments per kilobase of transcript per million fragments mapped (FPKM) value of 15,829 (Table S3). CFEM domain proteins are specific to fungi (18) and are involved in differentiation, stress responses, and pathogenicity (19). Eleven proteins with CFEM domains (CcCFEM1 to CcCFEM11) were identified in *C. cordycipiticola*, and most *Cccfem* genes were highly expressed during infection (Table S5).

### Transcriptional response of Cordyceps militaris induced by infection with *Calcarisporium cordycipiticola*.

For *C. militaris*, 914 and 1,548 DEGs, which were 9.47% and 16.04% of the total genes, were identified at 4 and 8 dpi compared with CM, respectively (Fig. 3C). There were many more DEGs at 8 dpi, indicating that *C. militaris* may respond slowly. The number of upregulated genes was higher than the number of downregulated genes at both 4 and 8 dpi (Fig. 3D).

GO analysis showed that DEGs were enriched for 13 and 15 terms at 4 and 8 dpi, respectively (*P < *0.01) (Fig. 4B). Among them, 4 terms were shared, and “heme binding” was the most enriched term at both stages (Fig. 4B). KEGG analysis showed that 5 and 7 significant pathways were enriched at 4 and 8 dpi, respectively. 2-Oxocarboxylic acid metabolism was the most significant pathway at both stages (corrected *P* value of <0.05) (Table S2). 2-Oxocarboxylic acids, also called 2-oxo acids and α-keto acids, are the most elementary set of metabolites, which include pyruvate (2-oxopropanoate), 2-oxobutanoate, oxaloacetate (2-oxosuccinate), and 2-oxoglutarate (20). “Butanoate metabolism” was especially enriched at 4 dpi, and “biosynthesis of amino acids,” “biosynthesis of antibiotics,” and “arginine biosynthesis” were especially enriched at 8 dpi (Table S2). Both GO and KEGG analyses showed that the pathogen’s attack substantially altered the primary metabolism of *C. militaris*.

**FIG 5:**
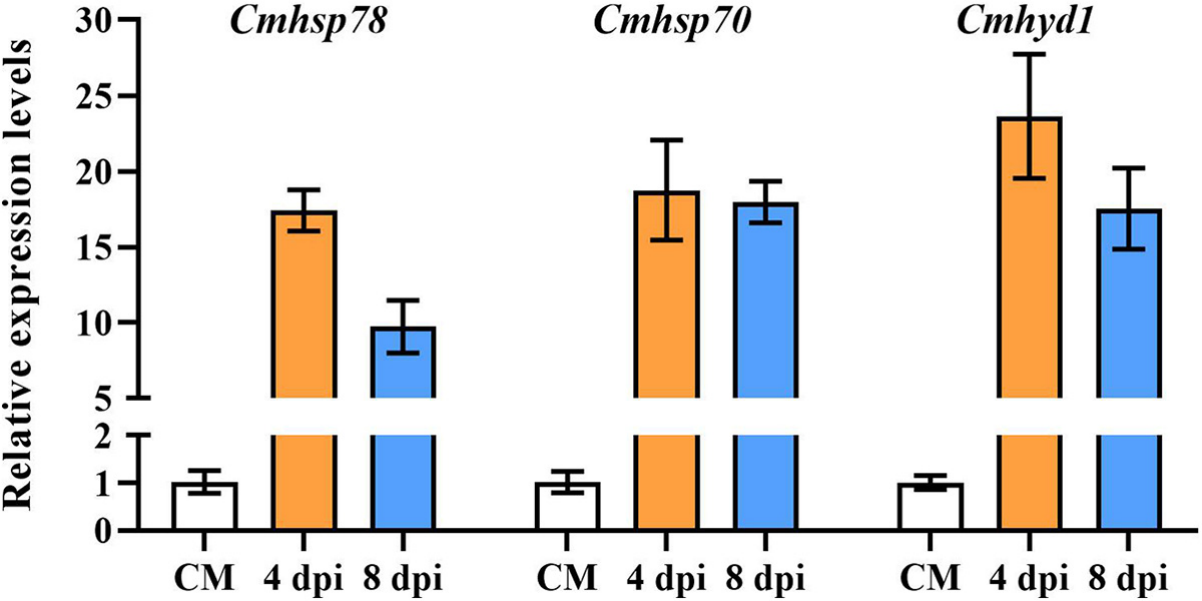
Transcription of *Cmhsp78*, *Cmhsp70*, and *Cmhyd1* at CM and 4 and 8 dpi. Gene transcription was normalized to the expression of *Cmrpb1* (*CCM_05485*). *Cmhsp78* (*CCM_05892*), *Cmhsp70* (*CCM_03788*), and *Cmhyd1* (*CCM_03537*) were upregulated at 4 and 8 dpi. Error bars indicate standard deviations.

Among all of the genes of *C. militaris*, the most upregulated gene was *CCM_05892* encoding CmHSP78, which showed a 10.26-fold change at 4 dpi compared with CM (Table S3). Fifty-one heat shock protein (HSP)-encoding genes were identified in the genome of *C. militaris* (Table S6), among which 20 were expressed differently at 4 and 8 dpi. Most of the differentially expressed *hsp* genes (75%; 15/20) showed higher expression levels at 4 dpi than at 8 dpi, especially *Cmhsp70* genes (Table S6). This was in sharp contrast to the fact that there were many more DEGs at 8 dpi than at 4 dpi for *C. militaris* (Fig. 3D), indicating that *hsp* genes are involved in the early response to infection. The upregulation of *Cmhsp78* (*CCM_05892*) and *Cmhsp70* (*CCM_03788*) after infection for 4 days was confirmed by quantitative real-time PCR (qPCR) (Fig. 5). *Cmhsp78* and *Cmhsp70* qPCR primers are listed in Table S7.

The DEG with the highest transcription level at 4 dpi was *Cmhyd1* (*CCM_03537*), one of the hydrophobin genes. It was expressed with an average FPKM value of 11,334 and showed 5-fold upregulation at 4 dpi, which then decreased slightly at 8 dpi (Table S3). Four hydrophobin genes, termed *Cmhyd1* to *Cmhyd4*, were identified in the *C. militaris* genome (21). There was no response of *Cmhyd2* to *Cmhyd4* at the infection stage, and they maintained low expression levels. The upregulation of *Cmhyd1* was confirmed by qPCR (Fig. 5). It was speculated that *Cmhsp78*, *Cmhsp70*, and *Cmhyd1* were related to the defense response.

### Genes predicted to be involved in recognition and sensing between mycoparasites and hosts.

Genes involved in the recognition of fungal prey include those that encode G-protein-coupled receptors (GPCRs) and lectins (22, 23). All 23 predicted GPCR genes in the genome were expressed in samples from the three stages. The genes *CCOR_06769* (encoding a plasma membrane protein Pth11-like protein) and *CCOR_05453* (encoding microbial opsins) showed the highest transcription levels at 4 dpi (Table S8). Fourteen lectin-related genes were screened in *C. cordycipiticola*, and six were upregulated at 4 dpi. Five of them showed the highest transcription levels at 4 dpi, including three encoding proteins with a ricin-type β-trefoil lectin domain (*CCOR_01894*, *CCOR_03676*, and *CCOR_08831*) and two encoding proteins with a legume-like lectin family domain (*CCOR_01446* and *CCOR_08418*) (Table S9).

The recognition of pathogen-associated molecular patterns (PAMPs) is a fundamental host survival mechanism (24). There have been almost no reports on the PAMPs of mushrooms. The transcripts of 18 genes encoding lectins in *C. militaris* were analyzed (Table S9), and it was found that there was no distinct differential expression at both 4 and 8 dpi compared with CM. Only the *CCM_05447* gene encoding concanavalin A-like lectin and the *CCM_07787* gene encoding mannose-binding lectin were upregulated slightly at 4 or 8 dpi, and two genes encoding proteins with a ricin-type β-trefoil lectin domain (*CCM_03178* and *CCM_03832*) were downregulated slightly at 4 or 8 dpi.

### Differential expression of CAZymes in *Calcarisporium cordycipiticola* and Cordyceps militaris.

**FIG 6:**
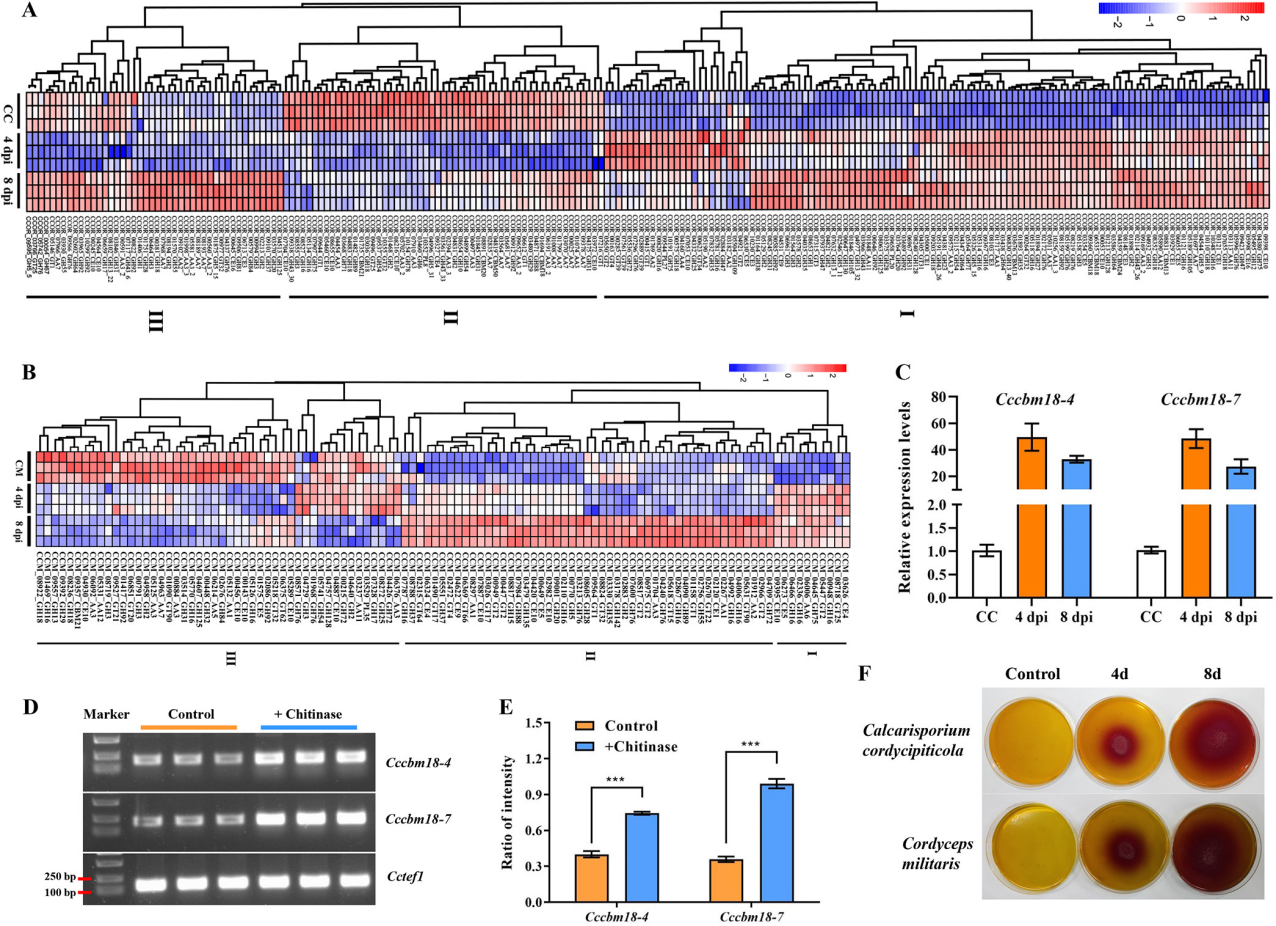
Transcript levels of two genes encoding CBM18 and chitin degradation abilities of *Calcarisporium cordycipiticola* and Cordyceps militaris. (A and B) Heatmaps of the differentially expressed CAZyme genes of *C. cordycipiticola* (A) and *C. militaris* (B). (C) Transcript levels of *Cccbm18-4* (*CCOR_06535*) and *Cccbm18-7* (*CCOR_09360*) at CC and 4 and 8 dpi confirmed by qPCR. Gene expression was normalized to the expression of *Cctef1* (*CCOR_04235*). *Cccbm18-4* and *Cccbm18-7* were upregulated at 4 dpi compared with CC. Error bars indicate standard deviations. (D and E) Transcript levels of *Cccbm18-4* and *Cccbm18-7* under chitinase stress determined by semiquantitative analysis. Gene expression was normalized to the expression of *Cctef1*. *** indicates a significant difference (*P < *0.001). (F) Chitin degradation by *C. cordycipiticola* and *C. militaris*. *C. militaris* has a stronger ability to produce chitinase than *C. cordycipiticola*.

Among the 441 predicted genes encoding carbohydrate-active enzymes (CAZymes) in the genome of *C. cordycipiticola* (16), 213 (48%) were differentially expressed at 4 or 8 dpi compared with CC (Table S10). The genes were grouped into three main clusters according to their expression patterns (Fig. 6A). The genes of cluster I showed upregulation at both 4 and 8 dpi and occupied 53.52% (114/213) of the total differentially expressed CAZyme genes. There were 55 (25.82%; 55/213) genes in cluster II, and they were downregulated after infection. These CAZymes should be related to carbohydrate metabolism when *C. cordycipitcola* is saprophytic. The genes of cluster III (20.66%; 44/213) were expressed at the highest levels at 8 dpi, and they should respond slowly during infection (Fig. 6A; Table S10).

The most upregulated CAZyme gene at 4 dpi was *CCOR_03676*, which had very low expression levels at CC and was upregulated by over 8- and 7-fold at 4 and 8 dpi compared with CC (Table S10). *CCOR_03676* encodes a protein of glycoside hydrolase family 76 (GH76), which is an endo-acting α-mannanase for degrading α-1,6-manooligosaccharides or the α-1,6-linked backbone of fungal mannoproteins (25). Among the 10 predicted GH76-encoding genes in the genome of *C. cordycipiticola* (16), 7 showed upregulation at 4 and 8 dpi compared with CC (Table S10). It was reported previously that there was 9.0 to 17.5% mannose in the cell wall of the host *C. militaris* (26), indicating that the degradation of the cell wall of the host is important for infection by the pathogen *C. cordycipiticola*.

The second and third most upregulated CAZyme genes at 4 dpi were *CCOR_09360* (*Cccbm18-7*) and *CCOR_06535* (*Cccbm18-4*), both belonging to carbohydrate-binding module 18 (CBM18) (Table S10). qPCR confirmed that *Cccbm18-7* and *Cccbm18-4* were highly expressed at 4 and 8 dpi, respectively (Fig. 6C). CBM18 is a subclass of chitin-binding domains that have convergently evolved in fungi, plants, and arthropods (27). There were 7 predicted proteins (CcCBM18-1 to CcCBM18-7) in the genome of *C. cordycipiticola* with CBM18 domains, which can be identified as Pfam domain PF00187 (chitin binding 1) (16). Analysis of the protein sequences with InterPro predicted a secretory signal peptide in both the *Cccbm18-7*- and *Cccbm18-4*-encoded proteins, indicating that they are trafficked outside the cells through the secretion pathway and may function at the cell surface. When fungal chitinase was added to the medium of *C. cordycipiticola*, the transcription levels of *Cccbm18-4* and *Cccbm18-7* increased significantly (Fig. 6D and E). It was demonstrated previously that CBM18 genes can protect the fungus from chitinases in Batrachochytrium dendrobatidis (28). The chitinase-producing abilities of *C. cordycipiticola* and *C. militaris* were compared by staining with bromocresol purple, and it was found that *C. militaris* has a stronger ability to produce chitinase than *C. cordycipiticola* (Fig. 6F). It was hypothesized that CBM18 proteins in *C. cordycipiticola* can bind chitin in its cell wall and thereby prevent chitinase hydrolysis by *C. militaris*.

Other than GH76 (α-mannanase) genes, some GH16 (β-glucanase) and GH18 (chitinase) genes were also important for the degradation of the cell wall of the host. Among the 16 GH16-encoding genes, 7 were upregulated. *CCOR_10348*, *CCOR_05118*, and *CCOR_09367* were upregulated over 4-fold at 4 dpi compared with CC and maintained high expression levels at 8 dpi (Table S10). The content of glucan in the cell wall of *C. militaris* was 52 to 60% (26). The transcripts of the chitinase genes *CCOR_07055*, *CCOR_10110*, *CCOR_08352*, and *CCOR_00575* were upregulated over 3-fold at 4 dpi compared with CC, and high expression levels were maintained at 8 dpi, except for the expression of *CCOR_00575*, which decreased slightly at 8 dpi compared to that at 4 dpi (Table S10).

A total of 330 CAZyme-related genes were predicted in the genome of *C. militaris*, among which 107 (107/330; 32.4%) were differentially expressed at 4 and 8 dpi compared with CM (Table S10). These genes were grouped into three main clusters according to the expression patterns (Fig. 6B). Ten genes were grouped into cluster I and showed upregulation at 4 and 8 dpi. There were 48 (44.86%; 48/107) genes in cluster III, and they were downregulated after infection. The genes of cluster II (45.80%; 49/107) were expressed at the highest levels at 8 dpi (Fig. 6B; Table S10). The most upregulated CAZyme gene at 4 and 8 dpi was *CCM_04420*, which had very low expression levels at CM and was upregulated by over 4- and 6-fold at 4 and 8 dpi, respectively (Table S10). *CCM_04420* was classified as a carbohydrate esterase family 10 (CE10) gene. This family is currently canceled in the CAZyme classification as members appear to act on ester-based modifications of compounds other than carbohydrates (29). Actually, *CCM_04420* contained an acetyl esterase/lipase domain that was related to lipid transport and metabolism. Further analysis found that there were no homologs of this protein in the other species of Cordycipitaceae. The results of the phylogenetic analysis based on the protein sequences was totally incongruent with the species tree, and this gene was probably inherited from Diaporthomycetidae fungi (Fig. S4). Twenty-two GH18 genes were predicted in the genome of *C. militaris* (Table S11), more than in *C. cordycipiticola*. However, only 2 of them were downregulated at 4 dpi (Table S11), indicating that most chitinase genes did not respond to infection, although it was confirmed that *C. militaris* can produce chitinases at the mycelial stage (Fig. 6F).

### Responses of genes related to SMs in *Calcarisporium cordycipiticola* and Cordyceps militaris.

Among the 82 core genes of 66 SM clusters of *C. cordycipiticola* (16), 62 showed the lowest expression levels at 4 dpi (Table S12). It seemed that at 4 dpi, *C. cordycipiticola* grew mainly for colonization, and most of the SMs were inhibited temporarily. Sixteen core genes showed the lowest expression levels at CC and then showed an increasing trend at 4 or 8 dpi (Fig. S5 and Table S12), which should be related to interactions.

**FIG 7:**
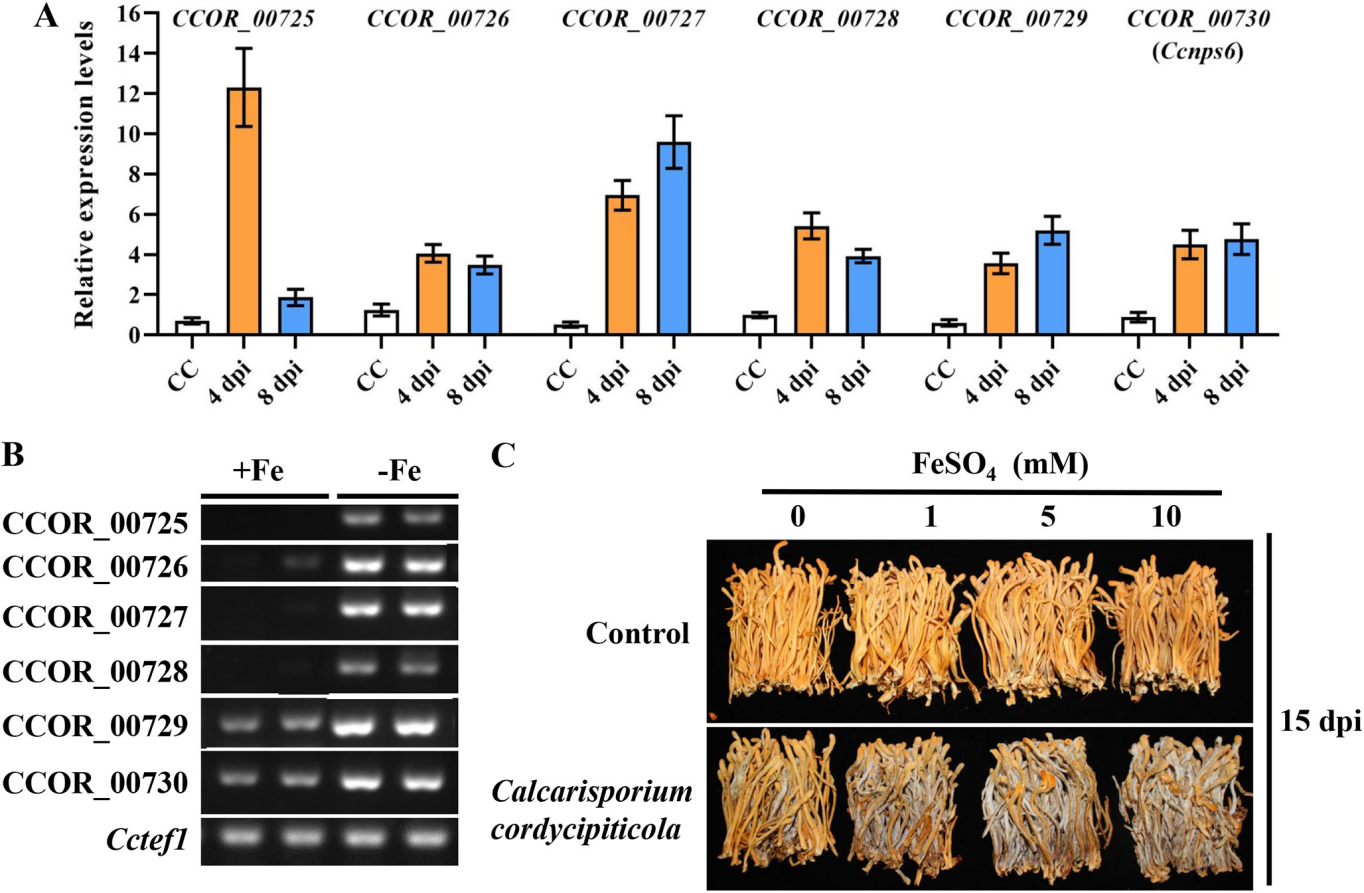
Transcript levels of the genes of the *Ccnps6* cluster and iron enhancement of the virulence of *Calcarisporium cordycipiticola*. (A) Transcript levels of the genes of the *Ccnps6* cluster of *C. cordycipiticola* confirmed by qPCR. (B) Genes of the *Ccnps6* cluster are upregulated under iron-depleted (−Fe) compared to iron-replete (+Fe) conditions in *C. cordycipiticola*. The expression levels of the control gene *Cctef1* are not affected by the presence or absence of iron. (C) The exogenous addition of iron enhances the virulence of *C. cordycipiticola*.

It was assumed that the *CCOR_00730* (*Ccnps6*) cluster was responsible for the biosynthesis of siderophores in *C. cordycipiticola* (16). There were 14 genes in the *Ccnps6* gene cluster (16), and most of them were highly expressed at the infection stage (Fig. 7A; Table S13). It was inferred that *C. cordycipiticola* upregulated the transcripts of genes of the *Ccnps6* cluster and synthesized a high level of siderophores during infection. A homologous cluster was also found in *C. militaris*, but it did not respond to infection (Table S13). The transcripts of genes of the *Ccnps6* cluster in *C. cordycipiticola* were highly induced by iron deficiency (Fig. 7B). The pathogen was in an iron-deficient environment at the initial stage of infection, and the genes of the *Ccnps6* cluster were induced for iron acquisition. The exogenous addition of a certain amount of iron (1, 5, and 10 mM FeSO_4_) distinctly increased pathogenicity (Fig. 7C), indicating that iron was an important factor for the virulence of *C. cordycipiticola*.

Among the 40 core genes of 30 SM clusters of *C. militaris*, 6 showed the lowest expression levels at CM and then showed an increasing trend at 4 and 8 dpi (Fig. S5 and Table S12). These genes and their products should be related to interactions. Among them, only 1 core gene (NRPS-like) (*CCM_08331*) showed >3-fold upregulation at both 4 and 8 dpi. Pfam annotated this product as “pyoverdine/dityrosine biosynthesis protein,” which may be an efficient iron(III) transporter. More than one-half of the core genes in *C. militaris* showed deceased transcription with infection.

### ROS-mediated interactions between Cordyceps militaris and *Calcarisporium cordycipiticola*.

**FIG 8:**
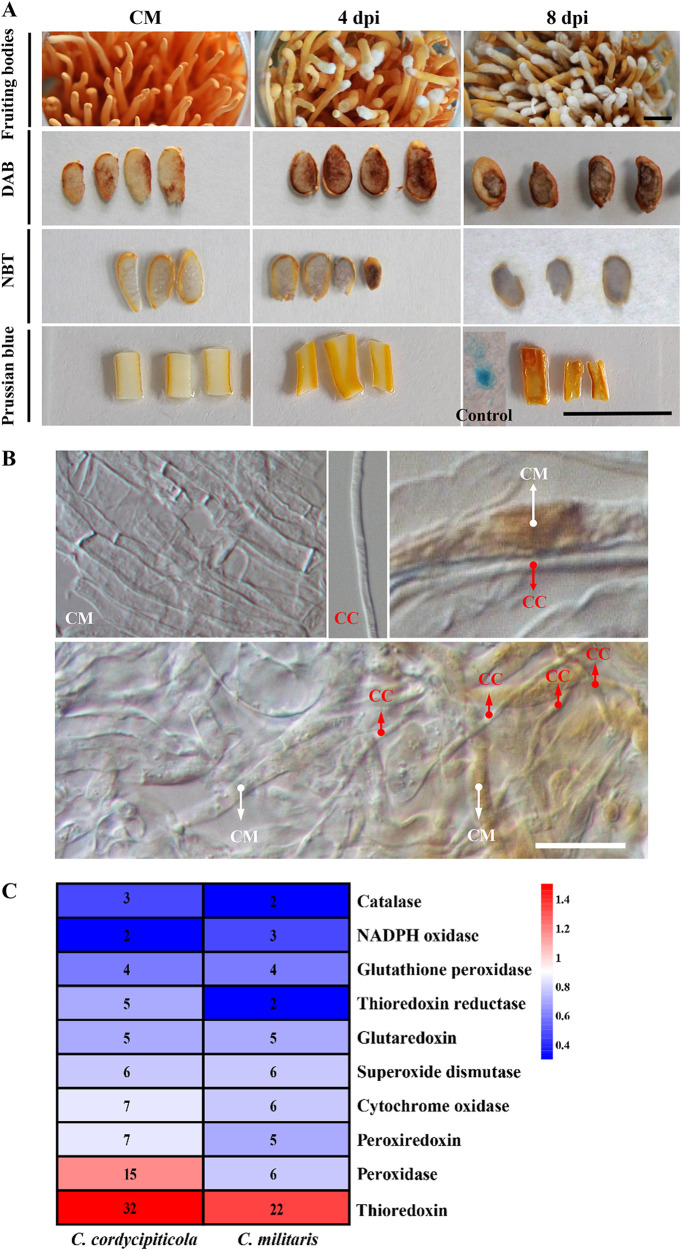
Hydrogen peroxide accumulated in Cordyceps militaris after being infected by *Calcarisporium cordycipiticola*. (A) H_2_O_2_ and iron concentrations in *C. militaris* fruiting bodies after being infected by *C. cordycipiticola* revealed by staining. DAB and NBT were used for staining for H_2_O_2_, and Prussian blue was used for staining for ferric acid. Control shows the color of the positive control. (B) H_2_O_2_ accumulated mainly in the hyphae of *C. militaris*. CC, hyphae of *C. cordycipiticola*; CM, hyphae of fruiting bodies of *C. militaris*. Bars = 1 cm (A) and 10 μm (B). (C) Genes related to antioxidant activity in *C. cordycipiticola* and *C. militaris*.

GO and KEGG analyses revealed that certain redox-related terms or pathways were enriched during infection in both *C. militaris* and *C. cordycipiticola* (Fig. 4; Table S2). The concentration of hydrogen peroxide increased significantly in the *C. militaris* fruiting bodies after being infected by *C. cordycipiticola* and accumulated with the extension of infection (Fig. 8A), but no ROS accumulated in the hyphae of *C. cordycipiticola* as revealed by diaminobenzidine (DAB) and nitroblue tetrazolium chloride (NBT) staining (Fig. 8B). *C. militaris* can produce excessive ROS to respond to infection, which was similar to the immunity reaction of some plants responding to the pathogen. However, no obvious accumulation of ferric iron was observed in the infected tissues by staining with Prussian blue (Fig. 8A).

*C. cordycipiticola* is more sensitive to H_2_O_2_ than *C. militaris* (Fig. S6), indicating that the pathogen *C. cordycipiticola* can remove H_2_O_2_ from the environment by some mechanisms. Other than the upregulation of the *Ccnps6* cluster and *Cccfem* genes (Fig. 7A; Table S5), to acquire an appropriate amount of iron, genes related to antioxidant activity should make a contribution.

Eighty-six genes related to antioxidant activity (superoxide dismutase [SOD], catalase [CAT], glutathione, thioredoxin, and peroxidase systems) were identified in *C. cordycipiticola* (Fig. 8C; Table S14), in which more peroxidases and thioredoxins were identified than in *C. militaris*. A total of 95.35% (82/86) of the genes were expressed (Table S14), and 31 DEGs were grouped into three clusters (Fig. S7). Genes of clusters I and II were upregulated after infection and can remove ROS produced by the host during interactions. Sixty-one genes related to antioxidant activity were identified in *C. militaris*, and 90.16% (55/61) were expressed (Fig. 8C; Table S14). Among these genes, 14 were differentially expressed during at least one stage (Table S14) and were grouped into 3 clusters, among which upregulated genes after infection were dominant (50%) (Fig. S7).

## DISCUSSION

Calcarisporiaceae and Cordycipitaceae are sister families in Hypocreales (30). The parasite *C. cordycipiticola* and the host *C. militaris* are two phylogenetically close species. Here, time course dual RNA-seq was performed to reveal the pathogenic mechanisms of *C. cordycipiticola* and the responses of *C. militaris* to infection. *C. cordycipiticola* and *C. militaris* were in a dynamic battle, and the host *C. militaris* responded more slowly than the pathogen. *C. cordycipiticola* may secrete a large number of effectors to invade the host and evade the defense reactions of the host. Iron and ROS regulated the interaction between *C. cordycipiticola* and *C. militaris*. The *Cmhsp70*, *Cmhsp78*, and *Cmhyd1* genes of *C. militaris* responded to the infection quickly, and they may be used as target genes for the breeding of resistant varieties. This study will be helpful for understanding the mycoparasitic interactions of two phylogenetically close species in their natural setting.

In our previous study, the process of infection of *C. cordycipiticola* in the fruiting bodies of *C. militaris* was monitored (16). It was found that the mycelia of *C. cordycipiticola* proliferated on the surface of the fruiting bodies of *C. militaris* at 3 to 4 dpi, and visible mycelia of *C. cordycipiticola* appeared on the fruiting bodies of *C. militaris* at 4 dpi. The color of the interior of the fruiting bodies gradually became deeper at 7 to 8 dpi. These two stages were key nodes of the infection. However, different infection stages should have their own controls in the best experimental scheme, which we will pay more attention to in our future research.

The pathogenic strategies of *C. cordycipiticola* may include (i) recognition and sensing of the host, (ii) colonization by rapid growth, (iii) suppression of the immune response of the host, (iv) nutrient absorption, (v) killing and degradation of the host, and (vi) adaptation to the microenvironment of the host. The first key step in a successful mycoparasite’s life history is the ability to recognize and sense its host (31). Since early samples for transcriptome analysis were selected at 4 dpi, there was a possibility that the differential expression of genes responsible for recognition was not detected. As for the present data, lectins of pathogens may play a more important role in early recognition (see Table S9 in the supplemental material). The pathogen then regulated the expression of ribosome synthesis genes to synthesize a large number of proteins and promoted its growth and development. More secreted proteins were produced through the type II secretion system (Fig. 4), some of which continued to decompose the host cells and obtain nutrients and others of which surrounded their own cell walls to stabilize the internal environment for their growth.

In general, the host *C. militaris* responded more slowly than the pathogen (Fig. 3). Since *C. cordycipiticola* is the invader, it needs to mobilize more resources to invade and seize favorable ecological niches at the early stage of infection (4 dpi). Although the biomass of *C. cordycipiticola* was not high at the early stage, its gene expression was active, while *C. militaris* was in a defensive state at 4 dpi and was slow to respond to the outside world, and not many genes were mobilized. Later (8 dpi), the infection was severe, and more defense genes of *C. militaris* were mobilized to fight the pathogen.

During the colonization of the host, CAZymes were produced by both *C. cordycipiticola* and *C. militaris* (Table S10). However, *C. cordycipiticola* upregulated some CAZyme genes quickly at 4 dpi and slowly in the host *C. militaris*, which was consistent with the transcription trends of most DEGs. *C. cordycipiticola* is a destructive mycoparasite that kills the host mainly by secreting cell wall-degrading enzymes (GH2, GH16, GH18, and GH76) and extracellular metalloproteases, etc. Finally, the cells of *C. militaris* were broken and died, leading to the wilting of fruiting bodies (16). The top 3 upregulated CAZyme genes at 4 dpi in *C. cordycipiticola* were *CCOR_03676* (GH76), *CCOR_09360*, and *CCOR_06535* (CBM18), whereas *CCM_04420* (CE10), CCM_06273 (CE5), and *CCM_08571* (GH76) were the top 3 upregulated CAZyme gene at 4 dpi in *C. militaris*. Since *C. militaris* has a stronger ability to produce chitinase than *C. cordycipiticola* (Fig. 6D to F), it was speculated that *C. cordycipiticola* can secrete CBM18 proteins with a chitin-binding domain to bind chitin on its cell wall and thereby prevent chitinase hydrolysis by the host *C. militaris*, which may be part of defending host immunity. This strategy has also been found for the plant pathogen Plasmodiophora brassicae (32).

“Heme binding” (GO:0020037) was the only enriched GO term shared by both the pathogen and the host at both stages at 4 dpi and 8 dpi compared with CC or CM (Fig. 4). This GO term was defined as “binding to a heme, a compound composed of iron complexed in a porphyrin (tetrapyrrole) ring.” Four pathways for iron acquisition have been identified in fungi: the siderophore-dependent pathway, the reductive pathway, the low-affinity ferrous iron uptake pathway, and the heme assimilation pathway (33). Heme constitutes a major source of iron for microorganisms (34), indicating that iron is important for both the pathogen *C. cordycipiticola* and the host *C. militaris*. Due to the scarcity of free iron in the host environment, pathogenic microorganisms by necessity have evolved mechanisms to extract iron from the host. Here, *C. cordycipiticola* was confirmed to upregulate the transcription of not only genes of the *Ccnps6* cluster to produce many more siderophores to acquire iron from the host but also genes of the heme assimilation pathway. It was reported previously that CFEM proteins contributed to the acquisition of two forms of iron, heme and ferric ion, in the entire life cycle of Beauveria bassiana (33). The CFEM family members of Verticillium dahliae diverged to have different functions in iron predation and immunity suppression, both of which were necessary for colonization and propagation in the iron-insufficient environment of host plants (35). It was inferred that CFEM proteins may also help *C. cordycipiticola* with iron acquisition. There was an entirely different response of the host *C. militaris* since it can obtain iron from the medium. Genes of the siderophore cluster did not respond to infection. However, it may regulate the heme assimilation pathway to compete with the pathogen. This should be one of the defense mechanisms.

In plant- and animal-pathogenic fungi, iron has been characterized as a virulence determinant (36). The function of iron is still questioned in fungus-fungus interactions. In this study, we confirmed that iron is also a virulence determinant in *C. cordycipiticola* infecting *C. militaris*, which is the first report that iron regulates the virulence of pathogens on mushrooms. Increases in the iron concentrations in the air will accelerate outbreaks of this disease. It was concluded that avoiding the use of iron frames is beneficial for disease control during the cultivation of *C. militaris.*

During the interaction between *C. militaris* and *C. cordycipiticola*, ROS played an important role in both the defense response of *C. militaris* and infection by the pathogen. When *C. cordycipiticola* invades, the host *C. militaris* may, like plant hosts such as rice, induce the expression of its disease resistance genes and produce a hypersensitive response through an oxygen burst, thereby preventing the further expansion of the pathogen. Invasion by *C. cordycipiticola* resulted in the accumulation of excessive ROS in the hyphae of fruiting bodies of *C. militaris* (Fig. 8B), causing much molecular damage such as protein oxidation, DNA mutation, and lipid peroxidation. During the infection process of *C. cordycipiticola*, a small number of hyphae will penetrate the hyphae of *C. militaris* (16), exposing it to an environment with a high level of reactive oxygen, but the hyphae can still grow normally (16), indicating that the pathogen should be able to scavenge reactive oxygen and adapt to the host environment. Genes related to ROS scavenging in *C. cordycipiticola* were also differentially expressed at different levels, and these genes may play an important role in regulating the homeostasis of ROS in their hyphae.

PAMPs can efficiently eliminate invading pathogens and have been researched in detail in both plant and animal hosts. Here, there was no distinct differential expression of 18 lectin-encoding genes at both 4 and 8 dpi compared to CM for the host (Table S9). Whether the host *C. militaris* could perceive the presence of pathogens well is worth studying in the future since there are almost no reports on the PAMPs of mushrooms infected by fungal pathogens.

The pathogen’s attack substantially altered the primary metabolism of the host *C. militaris*, as seen by the fact that only KEGG pathways of amino acid and 2-oxocarboxylic acid (Table S2) were enriched at both 4 and 8 dpi. It was also reported previously that *Rhizoctonia* infection alters the primary metabolism of soybean seedlings, including carbohydrate and amino and carboxylic acid metabolisms (37).

The primary and secondary metabolisms of both the pathogen *C. cordycipiticola* and the host *C. militaris* were changed during the interaction process. The difference is that the pathogen enhances them to promote colonization, while primary and secondary metabolic disorders occurred in the host *C. militaris*. Other than producing excess ROS, the defense mechanisms of *C. militaris* may include the quick upregulation of *Cmhsp78*, *Cmhsp70*, and *Cmhyd1*. In addition to serving as molecular chaperones, HSPs have been found to play essential and regulatory roles in the innate immune responses of plant (38) and animal (39) cells. According to our previous study on the function of *Cmhyd1* (21), it was speculated that CmHYD1 may be secreted to the mycelial surface of *C. militaris*, hinder the recognition of the pathogen, and, thus, improve the disease resistance of *C. militaris*. No *C. militaris* strain resistant to *C. cordycipiticola* has been found; there is an urgent need to explore potential resistance genes for the breeding of *C. militaris*. *Cmhsp78*, *Cmhsp70*, and *Cmhyd1* may be candidate genes.

## MATERIALS AND METHODS

### Strains and culture conditions.

*C. cordycipiticola* strain CGMCC 5.2193 was isolated from diseased fruiting bodies of *C. militaris* strain CGMCC 3.16320 in our laboratory (16). Both strains were maintained on potato dextrose agar (PDA) at 20°C. The fruiting bodies of *C. militaris* were cultivated according to previously reported methods (40).

### Collection of samples for dual RNA-seq.

*C. cordycipiticola* was grown on PDA medium in the dark at 20°C for 7 days and then used to infect 4 to 5-cm-long fruiting bodies of *C. militaris*. Infection was performed according to our previously described methods (16), with modifications. Discs of mycelia (1 cm in diameter) of *C. cordycipiticola* cultured on PDA were cut with a puncher. Ten pieces of mycelia were inoculated onto the surface of fruiting bodies in each cultivation bottle. *C. militaris* pathogenic sites (1 cm^2^) were harvested after infection for 4 and 8 days and named as 4 dpi and 8 dpi, respectively. The mycelia of *C. cordycipiticola* grown on PDA medium covered with a sheet of sterile cellophane in the dark at 20°C for 7 days (CC) and the fruiting bodies of *C. militaris* (CM) used for infection were harvested as controls. CC was collected by scratching fungal mycelia from the cellophane using a sterile spatula. CM was harvested and cut into small pieces. All samples were immediately frozen in liquid nitrogen and stored at −80°C until RNA was extracted. Samples from each of the three different bottles were defined as three biological replicates for each treatment. Three biological replicates were included for each treatment, and 12 samples were used for dual-RNA-seq analysis.

### RNA isolation, library construction, and sequencing.

Dual RNA-seq was performed according to the flow diagram shown in Fig. S1 in the supplemental material. First, total RNA was extracted and sequenced. Next, clean reads were mapped to the reference genome sequences of *C. cordycipiticola* and *C. militaris*. Finally, gene transcription was analyzed as usual.

Total RNA was extracted using an Omega plant kit (Omega Engineering, Norwalk, CT, USA). The RNA concentration was measured using a NanoDrop 2000 instrument (Thermo Fisher Scientific, Waltham, MA, USA), and the RNA integrity was assessed using the RNA Nano 6000 assay kit for the Agilent Bioanalyzer 2100 system (Agilent Technologies Inc., Palo Alto, CA, USA). Twelve libraries were generated using a NEB Next Ultra RNA library prep kit (New England BioLabs [Beijing] Ltd., Beijing, China) according to the manufacturer’s recommendations and sequenced (paired end, 150 bp each) on an Illumina HiSeqX-ten platform (Illumina Inc., San Diego, CA, USA) by Biomarker Technology Co. Ltd. (Beijing, China).

### Read mapping and RNA-seq statistical analysis.

Raw data (raw reads) in fastq format were first processed through in-house Perl scripts based on FastQC (https://raw.githubusercontent.com/s-andrews/FastQC/master/INSTALL.txt). In this step, clean data (clean reads) were obtained by removing reads containing the adapter or poly(N) and low-quality reads from the raw data. All of the downstream analyses were based on clean data of high quality.

For the pathogen *C. cordycipiticola* and the host *C. militaris*, clean reads were aligned to the reference genomes *C. cordycipiticola* CC01 (16) and *C. militaris* CM01 (41), respectively, using HISAT2 (42). The above-described reads were assembled using StringTie (43). The gene expression levels were estimated by the FPKM.

Pearson’s correlation coefficients of gene expression between replicates were used to determine whether the biological replicates were sufficiently similar for subsequent statistical analysis (44). The host and pathogen transcriptomes were subjected to differential expression analysis using the DESeq2 R package (45). A corrected false discovery rate (FDR) of 0.01 and a log_2_ fold change of 1 were set as the thresholds for differential expression unless specified otherwise.

### Functional annotation of DEGs.

The GO enrichment analysis of the DEGs was performed using the GOseq R package based on a Wallenius noncentral hypergeometric distribution, which can be adjusted for gene length bias in DEGs (46). The redundancy in the significantly enriched GO terms was eliminated by using REVIGO (http://revigo.irb.hr/). KOBAS software was used to test the statistically significant enrichment of DEGs in KEGG pathways (47).

### Reverse transcription-PCR and qPCR analyses.

The synthesis of cDNA from RNA (1 μg) was carried out using a HiScript III 1st-strand cDNA synthesis kit (+gDNA wiper) (Vazyme Biotech Co. Ltd., Beijing, China). For the semiquantitative analysis, the intensity of each band in the gel images was measured using ImageJ software (https://imagej.net/ij/index.html), and the ratio of the expression level of the tested gene to the expression level of *Cctef1* (*CCOR_04235*) was calculated. The semiquantitative data sets with and without chitinase were compared via Student’s *t* test.

qPCR was conducted using AceQ qPCR SYBR green master mix (Q111) (Vazyme Biotech Co. Ltd., Beijing, China) with the CFX Connect real-time system (Bio-Rad, Hercules, CA, USA). The relative gene expression levels were calculated using the 2^−ΔΔ^*^CT^* method (48). The *Cctef1* (*CCOR_04235*) and *Cmrpb1* (*CCM_05485*) genes were used as internal standards for *C. cordycipiticola* and *C. militaris*, respectively. All of the primer sequences used in this study are listed in Table S7. The obtained data represent the results from three biological replicates, with two technical replicates each.

### Determination of chitinase activity.

Chitinase activity was determined using bromocresol purple staining according to methods described previously (49), with modifications. Conidia of *C. cordycipiticola* (1 × 10^6^) were inoculated into medium with chitin colloid as the sole carbon source and bromocresol purple as an indicator and then cultured at 20°C. The color of the tested plates was observed.

### ROS detection.

The localization of ROS in *C. militaris* after infection for 4 and 8 days was visualized using DAB and NBT stainings, which were performed as described previously, with slight modifications (50). Briefly, sections with a thickness of 5 to 10 μm were cut transversely under a microtome (EM UC7; Leica, Wetzlar, Germany), treated with 0.05% (wt/vol) DAB and NBT in 50 mM phosphate-buffered saline (pH 7.4), and incubated for 20 min at 20°C. The buffer was then removed, and the sections with *C. cordycipiticola* were washed three times with distilled water. The slices were placed onto absorbent blot paper, observed under an anatomical lens and a light microscope, and photographed.

### Virulence assays and exogenous iron addition.

For the evaluation of the virulence of *C. cordycipiticola*, *C. militaris* fruiting bodies of 3 to 4 cm in length were spray inoculated with 2 mL of a spore suspension (5 × 10^6^ spores/mL) of *C. cordycipiticola* in 0.05% Tween 80. Inoculated *C. militaris* fruiting bodies were kept in a growth chamber under 12 h of light/12 h of darkness at 20°C with 90% relative humidity. Virulence was evaluated 15 days after inoculation, and the infected fruiting bodies were harvested from three independent tissue culture vessels. Ten infected fruiting bodies were randomly chosen from each tissue culture vessel, and the area of each lesion was measured by using Nano Measurer 1.2 (51).

To determine the effect of iron on infection by *C. cordycipiticola*, 3 mL of an iron solution (0, 1, 5, and 10 mM FeSO_4_ solution in 0.05% Tween 80) and *C. cordycipiticola* conidia were sprayed onto the surface of *C. militaris* fruiting bodies. The fruiting bodies sprayed with FeSO_4_ (0, 1, 5, and 10 mM FeSO_4_ solution in 0.05% Tween 80) were used as controls. Virulence assays were performed as described above.

### Data availability.

The raw data were deposited in GenBank under BioProject accession number PRJNA930069.
