# Supplementary material for: Dual Transcriptomics Reveals Interspecific Interactions between the Mycoparasite Calcarisporium cordycipiticola and Its Host Cordyceps militaris
Source: Microbiol Spectr. 2023 Mar 22;11(2):e04800-22. doi: 10.1128/spectrum.04800-22 (PMC10100745; doi:10.1128/spectrum.04800-22)
Supplement: Supplemental file 1 — Supplemental material. Download spectrum.04800-22-s0001.pdf, PDF file, 6.9 MB [file spectrum.04800-22-s0001.pdf]

Figure S1. Dual RNA-seq flow diagram

Figure S2. Pearson correlation between samples

Figure S3. The expression pattern of genes for secreted proteins, effector of *Calcarisporium cordycipiticola* during infection *Cordyceps militaris* at 4 and 8 dpi

Figure S4. Maximum-Likelihood (ML) phylogeny of homologs of *CCM_04420*

Figure S5. Differential expression of the core secondary metabolic genes in *Calcarisporium cordycipiticola* and *Cordyceps militaris* at different infected stages

Figure S6. Sensitivity of *Calcarisporium cordycipiticola* and *Cordyceps militaris* to H_2O_2

Figure S7. Heat map showing changes in expression of antioxidant-related genes in *Calcarisporium cordycipiticola* and *Cordyceps militaris* at different infected stages

Table S1 Summary of the sequencing data

Table S2 KEGG enrichment of DEGs in *Calcarisporium cordycipiticola* and *Cordyceps militaris*

Table S3 The top 20 up-regulated and highly expressed genes for 4 dpi vs CC and 4 dpi vs CM

Table S4 Differentially expressed secretory proteins and effectors at 4 and 8 dpi compared with CC

Table S5 The putative CFEM proteins in *Calcarisoprium cordycipiticola* (CC) and *Cordyceps militaris* (CM)

Table S6 Genes encoding for HSP in *Cordyceps militaris*

Table S7 Primers used in this study

Table S8 GPCRs genes in *Calcarisporium cordycipiticola*

Table S9 Lectin-related genes in *Calcarisporium cordycipiticola* (CC) and *Cordyceps militaris* (CM)

Table S10 DEG CAZyme genes in *Calcarisporium cordycipiticola* (CC) and *Cordyceps militaris* (CM)

Table S11 GH18 genes in *Cordyceps militaris*

Table S12 Core gene in *Calcarisporium cordycipiticola* (CC) and *Cordyceps militaris* (CM)

Table S13 Siderophore synthesis related gene clusters in *Calcarisporium cordycipiticola* (CC) and *Cordyceps militaris* (CM)

Table S14 ROS- related genes in *Cordyceps militaris* (CM) and *Calcarisporium cordycipiticola* (CC)

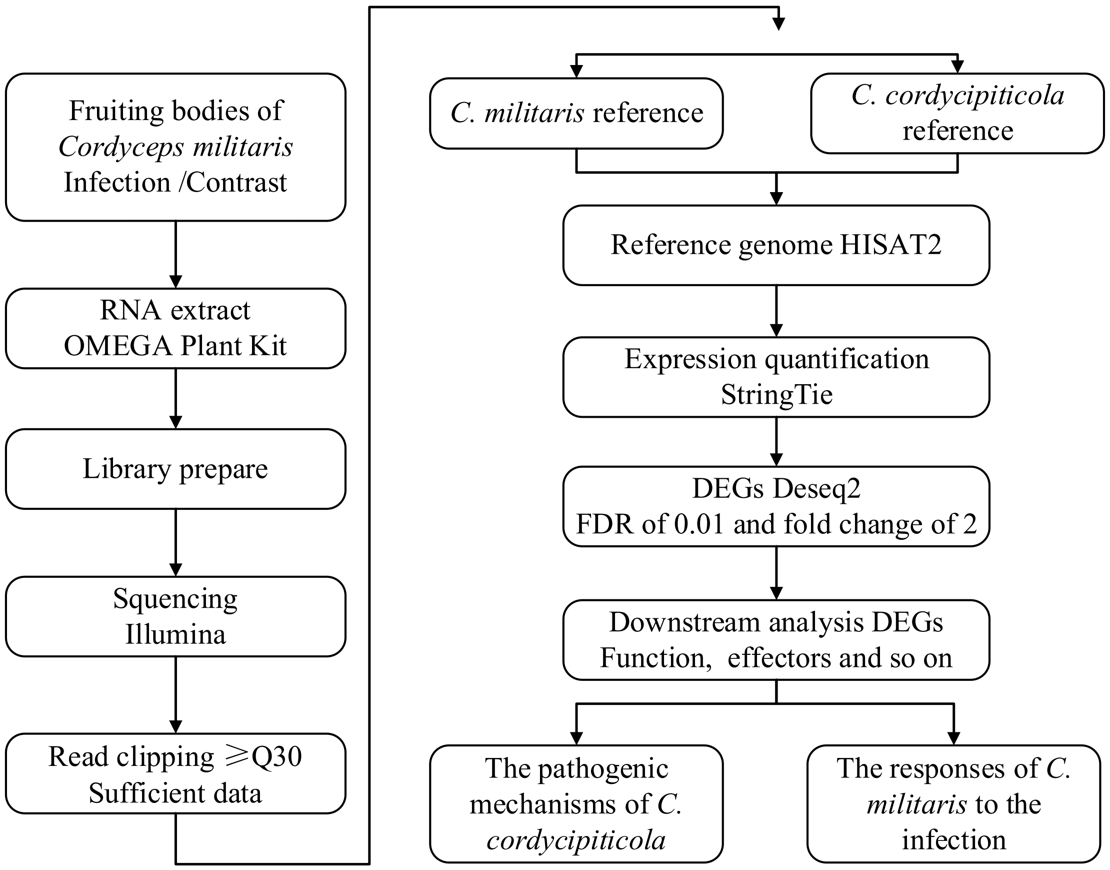

Figure S1. Dual RNA-seq flow diagram.

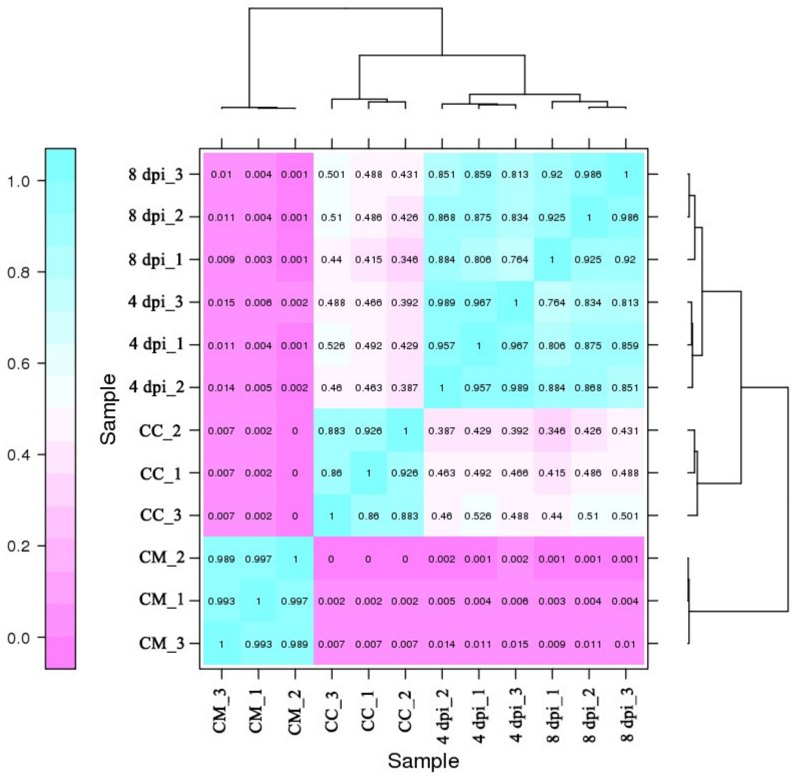

Figure S2. Pearson correlation between samples.

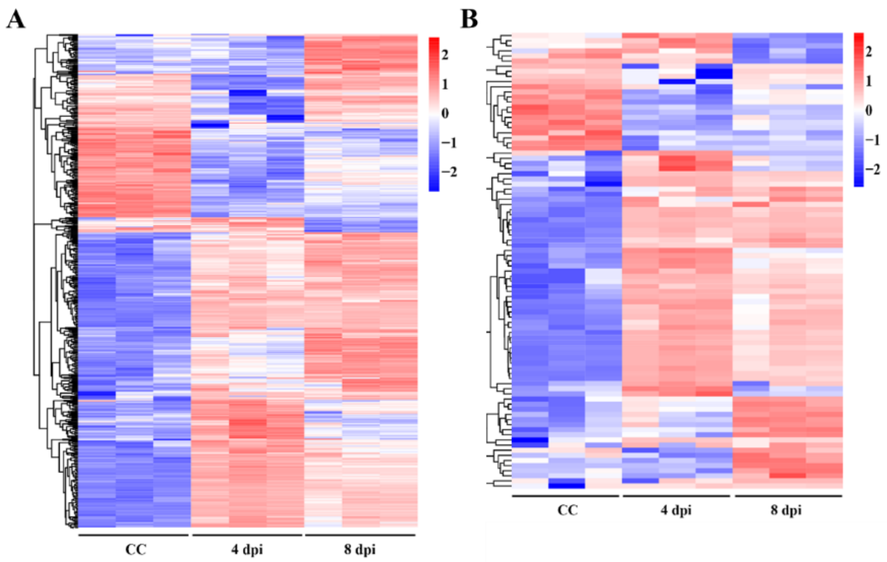

Figure S3. The expression pattern of genes for secreted proteins, effector of *Calcarisporium cordycipiticola* during infection *Cordyceps militaris* at 4 and 8 dpi. (A) Differentially expressed genes encoding predicted secreted proteins. (B) 89 genes encoding putative effectors were differentially expressed at 4 and 8 dpi compared with CC.

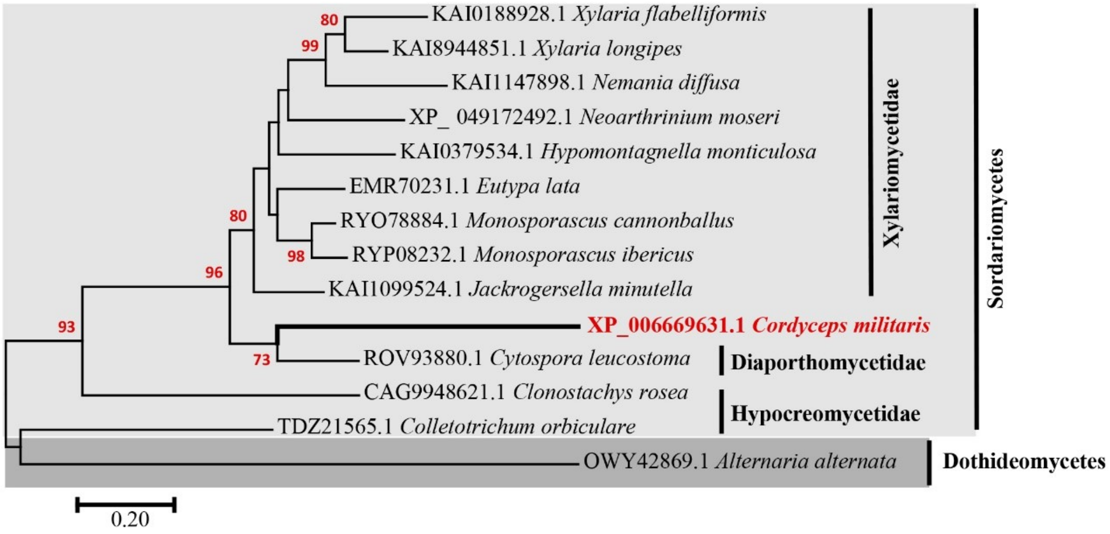

Figure S4. Maximum-Likelihood (ML) phylogeny of homologs of *CCM_04420*. The tree was reconstructed from an alignment of 389 variable amino acids. Bootstrap values greater than 70% are given at the nodes.

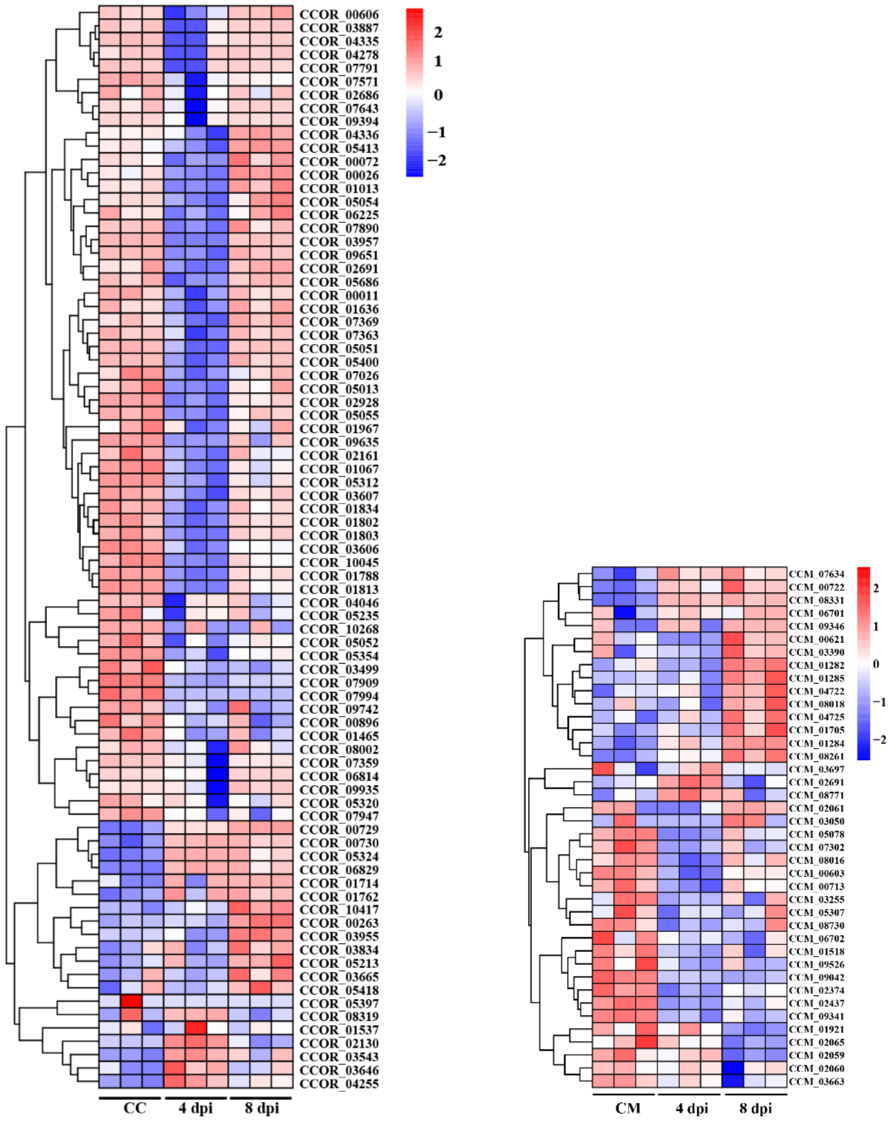

Calcarisporium cordycipiticola *Cordyceps militaris*

Figure S5. Differential expression of the core secondary metabolic genes in *Calcarisporium cordycipiticola* and *Cordyceps militaris* at different infected stages.

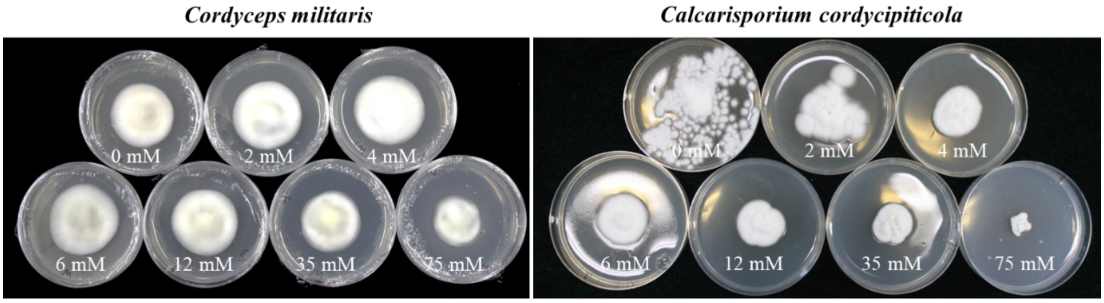

Figure S6. Sensitivity of *Calcarisporium cordycipiticola* and *Cordyceps militaris* to H_2O_2.

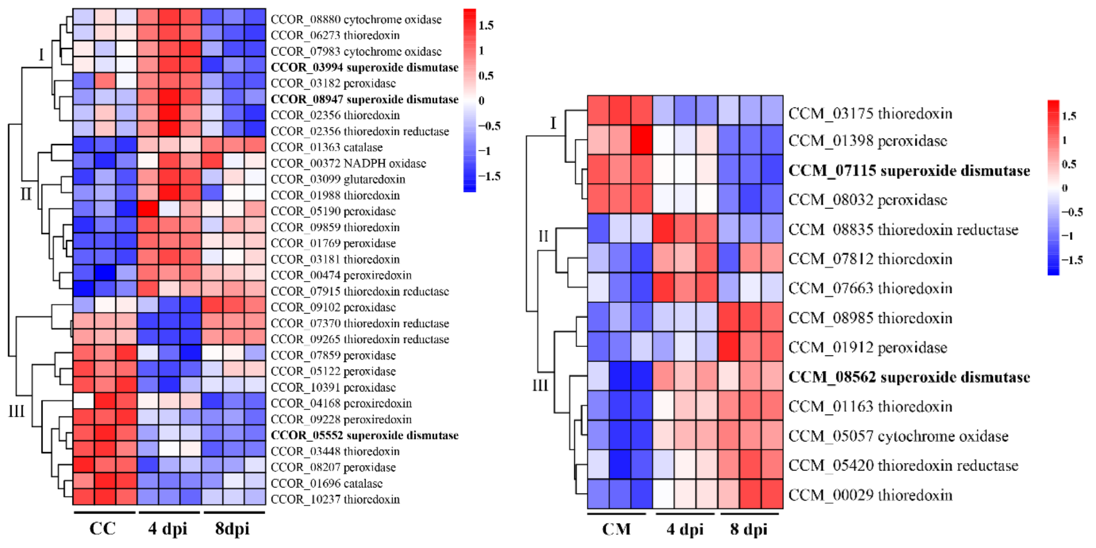

Calcarisporium cordycipiticola *Cordyceps militaris*

Figure S7. Heat map showing changes in expression of antioxidant-related genes in *Calcarisporium cordycipiticola* and *Cordyceps militaris* at different infected stages.

Table S1 Summary of the sequencing data.

Sample	Total reads	Clean bases (G)	%≥Q30	Mapped reads with *C. cordycipiticola* genome	Mapped reads with *C. militaris* genome
CC_1	56,328,294	8.42	92.84	52,698,051 (93.56%)	100,340 (0.18%)
CC_2	56,623,550	8.47	92.91	52,773,756 (93.20%)	204,710 (0.36%)
CC_3	71,537,130	10.7	92.79	66,357,180 (92.76%)	184,488 (0.26%)
CM_1	64,312,098	9.62	92.22	803,120 (1.25%)	55,366,768 (86.09%)
CM_2	63,044,102	9.43	91.74	1,357,794 (2.15%)	52,549,576 (83.35%)
CM_3	67,303,920	10.06	92.39	413,565 (0.61%)	58,140,216 (86.38%)
4_dpi_1	94,183,774	14.08	92.42	20,795,413 (22.08%)	64,745,076 (68.74%)
4_dpi_2	111,685,136	16.71	92.09	20,366,644 (18.24%)	79,627,892 (71.30%)
4_dpi_3	91,516,142	13.68	92.89	18,065,540 (19.74%)	65,847,305 (71.95%)
8_dpi_1	122,939,792	18.38	92.62	58,757,693 (47.79%)	55,829,249 (45.41%)
8_dpi_2	108,157,970	16.17	92.74	53,745,482 (49.69%)	46,797,363 (43.27%)
8_dpi_3	107,075,426	16.01	93.24	55,664,948 (51.99%)	44,551,082 (41.61%)

Table S2 KEGG enrichment of DEGs in *Calcarisporium cordycipiticola* and *Cordyceps militaris*.

Group	Pathway	KO	Enrichment_ Factor	*P*-value (<0.05)	Gene_ Number
4 dpi vs CC	Ribosome	ko03010	2.744799	1.45E-24	80
	Oxidative phosphorylation	ko00190	1.790524	2.82E-03	38
	Propanoate metabolism	ko00640	2.261714	2.22E-02	16
8 dpi vs CC	Ribosome	ko03010	3.263847	3.37E-16	53
	Starch and sucrose metabolism	ko00500	2.396225	2.46E-02	17
4 dpi vs CM	2-Oxocarboxylic acid metabolism	ko01210	4.155506	0.002317446	11
	Lysine degradation	ko00310	4.492804	0.006536185	9
	Alanine, aspartate and glutamate metabolism	ko00250	3.931203	0.020719056	9
	Tyrosine metabolism	ko00350	3.812076	0.026779365	9
	Butanoate metabolism	ko00650	4.659204	0.033097671	7
8 dpi vs CM	2-Oxocarboxylic acid metabolism	ko01210	3.877401	6.38E-06	18
	Biosynthesis of amino acids	ko01230	2.087437	1.24E-03	33
	Biosynthesis of antibiotics	ko01130	1.719714	1.64E-03	52
	Lysine biosynthesis	ko00300	4.25078	1.63E-02	8
	Arginine biosynthesis	ko00220	3.775364	2.01E-02	9
	Alanine, aspartate and glutamate metabolism	ko00250	2.98883	2.64E-02	12
	Tyrosine metabolism	ko00350	2.898259	3.69E-02	12

Table S3 The top 20 up-regulated and highly expressed genes for 4 dpi vs CC and 4 dpi vs CM.

Group	Gene ID	CC/CM_fpkm	4 dpi_fpkm	8 dpi_fpkm	4 dpi vs CC/4 dpi vs CM_log2FC	8 dpi vs CC/4 dpi vs CM_log2FC	4 dpi vs CC/4 dpi vs CM_FDR	8 dpi vs CC/8 dpi vs CM_FDR	function_annotation
The top 20 up-regulated and highly expression genes for 4 dpi vs CC	CCOR_08465	0.02	165.35	12.40	12.96	8.88	0.00	0.00	Beta/Gamma crystallin
	CCOR_08160	0.30	859.18	364.40	11.97	10.51	0.00	0.00	--
	CCOR_03663	0.05	128.75	69.51	11.76	10.51	0.00	0.00	Hypothetical protein
	CCOR_07851	1.25	2445.43	2508.52	11.49	11.18	0.00	0.00	Hypothetical protein
	CCOR_07855	0.02	55.99	22.99	11.33	9.68	0.00	0.00	Hypothetical protein
	CCOR_07896	0.11	185.08	31.56	11.22	8.32	0.00	0.00	2-oxoglutarate-dependent dioxygenase citB
	CCOR_00488	0.48	704.46	196.72	11.05	8.87	0.00	0.00	Hypothetical protein
	CCOR_09388	0.02	31.06	5.50	10.96	8.11	0.00	0.00	Hypothetical protein
	CCOR_00430	0.15	191.97	89.64	10.74	--	0.00	--	Glucose-6-phosphate 1-epimerase
	CCOR_08298	0.39	335.43	66.21	10.31	7.62	0.00	0.00	Probable extracellular serine carboxypeptidase
	CCOR_08239	0.04	32.90	13.65	10.25	8.64	0.00	0.00	Subtilisin-like serine protease PR1C, partial
	CCOR_10068	0.34	278.61	158.24	10.18	9.01	0.00	0.00	--
	CCOR_03917	0.98	655.10	456.55	9.95	9.09	0.00	0.00	Hypothetical protein
	CCOR_06890	0.12	81.25	38.69	9.83	8.42	0.00	0.00	Putative candidate effector 5 protein
	CCOR_00143	0.17	104.53	29.48	9.70	7.53	0.00	0.00	Repressed by EFG1 protein 1
	CCOR_08135	0.11	58.85	3.29	9.50	4.99	0.00	0.00	Extracellular metalloprotease
	CCOR_10440	0.31	152.04	86.77	9.50	8.36	0.00	0.00	Metallopeptidase, catalytic domain protein
	CCOR_08857	0.25	114.27	0.23	9.40	--	0.00	--	Heat shock protein 60
	CCOR_04710	0.45	174.02	72.60	9.13	7.52	0.00	0.00	Hypothetical protein
	CCOR_07502	0.11	42.00	0.12	9.05	--	0.00	--	Protein N-terminal amidase
The top 20 up-regulated genes for 4 dpi vs CC	CCOR_05591	3207.54	15829.92	12491.38	2.88	2.19	0.00	0.00	CFEM domain
	CCOR_01614	3243.87	9706.86	6996.47	2.12	1.26	0.00	0.00	Hypothetical protein
	CCOR_06200	3076.92	8875.09	3832.09	2.11	0.54	0.00	0.21	Clock-controlled protein 6
	CCOR_00474	928.77	6338.37	3609.64	3.34	2.19	0.00	0.00	Peroxisomal matrix protein
	CCOR_03749	3599.45	7089.09	2497.07	1.49	-0.36	0.00	0.71	Fungal hydrophobin
	CCOR_08998	1812.91	3560.75	2797.73	1.53	0.86	0.00	0.10	Heat shock 70 kDa protein

	CCOR_03744	2713.27	4184.35	2638.80	1.18	0.19	0.00	0.70	Cyclophilin A
	CCOR_00773	2960.66	3986.96	2625.16	1.00	0.06	0.01	0.93	Histone H4
	CCOR_00772	2588.71	3758.88	3196.31	1.11	0.53	0.00	0.14	Histone H3
	CCOR_06194	1342.18	2833.32	3792.85	1.64	1.72	0.00	0.00	Hypothetical protein
	CCOR_07310	1673.49	2507.22	2025.31	1.16	0.05	0.01	0.95	Clock-controlled protein 6
	CCOR_04431	542.71	2942.87	1053.74	3.02	1.18	0.00	0.00	60S ribosomal protein L18-B
	CCOR_01054	604.71	2763.64	1205.82	2.73	1.30	0.00	0.00	60S ribosomal protein L2
	CCOR_01093	709.41	2422.98	1306.34	2.34	1.11	0.00	0.00	40S ribosomal protein S18-B
	CCOR_01062	748.93	2661.96	1194.70	2.41	0.90	0.00	0.00	60S ribosomal protein L10a
	CCOR_05516	920.36	1785.23	1875.00	1.50	1.26	0.00	0.07	Heat shock protein 90 homolog
	CCOR_00424	509.76	2410.00	1011.78	2.82	1.22	0.00	0.00	60S ribosomal protein L20-B
	CCOR_04017	1011.26	2754.76	1677.34	2.03	0.96	0.00	0.00	CFEM domain
	CCOR_08348	741.18	2217.00	1207.99	2.13	0.94	0.00	0.00	40S ribosomal protein S23
	CCOR_04169	704.87	2250.69	1219.13	2.25	1.02	0.00	0.00	60S ribosomal protein L24
The top 20 up-regulated genes for 4 dpi vs cm	CCM_05892	0.12	149.97	175.76	10.26	10.23	0.00	0.03	Heat shock protein 78
	CCM_09298	0.00	2.48	3.87	9.13	9.49	0.00	0.00	Acetyltransferase
	CCM_00455	0.02	3.84	8.43	7.22	8.07	0.00	0.00	MFS nicotinic acid transporter Tna1, putative
	CCM_02078	0.27	33.46	0.23	7.00	--	0.00	--	Transcription factor Rba50, putative
	CCM_04721	0.36	43.50	45.43	6.93	6.70	0.00	0.00	Hypothetical protein
	CCM_05133	1.50	176.29	251.55	6.93	7.18	0.00	0.00	Zinc finger protein ZPR1
	CCM_04720	0.31	32.84	61.64	6.78	7.42	0.00	0.00	Hypothetical protein
	CCM_06106	0.12	9.60	10.48	6.32	6.16	0.00	0.00	Ankyrin repeat-containing domain
	CCM_03384	1.63	91.97	62.70	5.87	5.05	0.00	0.00	Hypothetical protein
	CCM_09153	1.38	70.41	304.30	5.85	7.73	0.00	0.00	Coenzyme A transferase
	CCM_04231	0.12	7.82	5.18	5.67	--	0.00	--	Alpha-1,3-mannosyltransferase (Alg3), putative
	CCM_06091	3.62	158.86	513.72	5.53	6.95	0.00	0.00	Cellobiose dehydrogenase
	CCM_01467	0.15	6.44	23.49	5.50	7.11	0.00	0.00	ABC multidrug transporter
	CCM_04719	0.32	11.95	12.74	5.28	5.09	0.00	0.00	Cytochrome P450
	CCM_09002	0.07	2.59	15.36	5.16	7.44	0.00	0.00	Extracellular metalloprotease
	CCM_00123	0.61	19.79	16.64	5.07	4.53	0.00	0.00	C6 transcription factor, putative

	CCM_00714	2.88	88.51	109.74	5.03	5.07	0.00	0.00	Conidiation-specific protein 13
	CCM_02598	10.01	274.26	526.29	4.84	5.50	0.00	0.00	ToxD-like zinc binding oxidoreductase, putative
	CCM_05222	12.97	301.97	277.49	4.61	4.23	0.00	0.01	Putative epoxide hydrolase
	CCM_00077	0.65	15.03	18.28	4.56	4.57	0.00	0.00	NADH-cytochrome b5 reductase 2
The top 20 up-regulated and highly expression genes for 4 dpi vs cm	CCM_03537	409.89	11334.40	1400.39	5.11	1.78	0.00	0.00	Fungal hydrophobin
	CCM_02125	1865.44	5221.90	5753.81	1.55	1.40	0.00	0.01	Hypothetical protein
	CCM_01459	1665.82	4010.33	1189.98	1.34	-0.70	0.00	0.12	Hypothetical protein
	CCM_09221	1084.12	2868.87	3281.27	1.47	1.38	0.00	0.00	heat-stable 19 kDa antigen precursor
	CCM_04804	1451.31	3692.87	4110.28	1.41	1.30	0.01	0.13	Heat shock 70 kDa
	CCM_07839	1063.53	3026.23	2822.30	1.55	1.19	0.00	0.17	Heat shock protein 90
	CCM_04921	308.48	2820.75	3364.61	3.26	3.22	0.00	0.00	Hypothetical protein
	CCM_09218	348.29	1713.01	2162.48	2.37	2.43	0.00	0.00	Hypothetical protein
	CCM_00003	380.16	1336.75	1141.97	1.88	1.37	0.00	0.02	Phosphoserine phosphatase
	CCM_00116	581.48	1304.08	1272.84	1.23	0.93	0.01	0.26	Heat shock protein
	CCM_09559	282.74	1523.26	563.98	2.49	0.77	0.00	0.43	Methyltransferase sdnD
	CCM_00947	374.39	1133.52	568.90	1.67	0.39	0.00	0.36	Beta-Ig-H3/Fasciclin
	CCM_07885	263.91	1160.91	677.07	2.21	1.16	0.00	0.04	Hypothetical protein
	CCM_03749	111.11	1187.83	1209.17	3.48	3.23	0.00	0.00	Saccharopine dehydrogenase
	CCM_08835	531.72	1223.01	504.59	1.28	-0.29	0.00	0.55	Probable thioredoxin reductase
	CCM_08296	62.38	1056.45	852.54	4.18	3.61	0.00	0.00	Hypothetical protein
	CCM_04511	132.65	1000.78	1585.08	2.98	3.37	0.00	0.00	Sarcosine oxidase
	CCM_07705	423.70	832.19	962.17	1.09	0.93	0.01	0.04	Hypothetical protein
	CCM_02954	103.78	505.79	341.45	2.07	1.49	0.00	0.05	Heat shock protein hsp88
	CCM_00632	226.26	846.38	623.31	1.96	1.25	0.00	0.23	Damage response protein 1

Table S4 Differentially expressed secretory proteins and effectors at 4 and 8 dpi compared with CC.

Numbers	Protein classification	Gene ID	CC_fpkm	4 dpi_fpkm	8 dpi_fpkm	4 dpi vs CC_log2FC	8 dpi vs CC_log2FC	4 dpi vs CC_FDR	8 dpi vs CC_FDR
1	SP	CCOR_00042	2.093	134.147	64.224	6.572	5.166	7.23E-24	2.07E-26
2	SP	CCOR_00054	1.858	0.000	5.582	--	1.808	6.98E-14	7.75E-08
3	SP	CCOR_00058	12.054	6.155	33.798	-0.414	1.714	0.6992957	2.47E-05
4	SP	CCOR_00059	34.261	12.343	10.456	-0.903	-1.483	0.0115517	1.64E-07
5	SP	CCOR_00062	1.604	2.749	5.227	1.372	1.921	0.0809118	1.23E-06
6	SP	CCOR_00063	22.289	1.507	5.662	-3.289	-1.751	2.06E-14	2.96E-07
7	SP	CCOR_00064	6.507	1.012	3.303	-2.076	-0.751	0.0008914	0.0622656
8	SP	CCOR_00077	541.080	1333.910	1599.889	1.874	1.789	2.69E-09	7.80E-11
9	SP	CCOR_00093	5.965	0.740	0.459	-2.290	-3.404	0.0398952	0.0015009
10	SP	CCOR_00138	48.455	8.379	8.353	-1.480	-1.827	0.0085398	0.0003594
11	SP	CCOR_00140	31.876	54.830	162.414	1.356	2.583	4.88E-05	1.93E-10
12	SP	CCOR_00143	0.168	104.528	29.478	9.695	7.527	1.62E-127	1.55E-54
13	SP	CCOR_00145	28.841	117.662	252.589	2.647	3.359	3.36E-16	2.71E-19
14	SP	CCOR_00154	972.479	1531.933	1614.235	1.231	0.960	0.002284	0.0084042
15	SP	CCOR_00181	23.567	64.200	86.182	2.003	2.102	1.17E-12	0.000994
16	SP	CCOR_00191	7.255	28.581	53.199	2.546	3.103	6.24E-05	5.91E-39
17	SP	CCOR_00210	11.379	2.880	5.127	-1.398	-0.916	0.0076837	0.0078577
18	SP	CCOR_00211	11.976	4.502	4.896	-0.819	-1.059	0.1439563	0.0048696
19	SP	CCOR_00242	0.004	1.887	29.042	7.424	--	2.77E-05	--
20	SP	CCOR_00313	63.179	95.330	54.652	1.174	0.019	0.0053618	0.9702079
21	SP	CCOR_00465	79.955	63.863	139.168	0.234	1.027	0.4002865	2.75E-05
22	SP	CCOR_00471	6.642	24.050	29.244	2.420	2.363	7.80E-08	3.10E-06
23	SP	CCOR_00481	6.736	1.235	2.398	-1.845	-1.264	0.0006299	0.0006845
24	SP	CCOR_00488	0.478	704.456	196.716	11.052	8.870	1.46E-179	2.78E-172

25	SP	CCOR_00500	430.777	32.791	124.430	-3.132	-1.564	9.95E-08	0.0006568
26	SP	CCOR_00529	6.742	2.170	2.950	-1.311	-1.393	0.009435	0.0018548
27	SP	CCOR_00531	31.835	557.446	95.136	4.707	1.810	3.03E-40	2.50E-11
28	SP	CCOR_00536	3.328	58.498	24.563	4.703	3.107	6.26E-30	3.29E-20
29	SP	CCOR_00578	11.380	29.650	38.806	1.953	1.998	0.0001668	3.02E-09
30	SP	CCOR_00579	14.694	15.484	42.914	0.623	1.774	0.1185953	8.47E-15
31	SP	CCOR_00581	4.991	53.761	130.783	3.991	4.941	2.28E-41	1.50E-77
32	SP	CCOR_00585	12.914	29.974	60.363	1.763	2.455	0.0018749	8.51E-12
33	SP	CCOR_00588	16.099	2.060	2.582	-2.380	-2.411	0.0039113	0.0008921
34	SP	CCOR_00593	101.684	23.441	48.224	-1.554	-0.847	2.88E-06	0.0005429
35	SP	CCOR_00645	3.376	4.215	28.075	0.891	3.282	0.1938227	1.84E-27
36	SP	CCOR_00646	19.821	41.702	127.528	1.633	2.913	9.69E-09	7.93E-31
37	SP	CCOR_00647	0.926	4.317	12.333	2.759	3.951	0.0033738	2.71E-11
38	SP	CCOR_00661	109.884	167.471	131.102	1.123	0.466	0.0002239	0.0773391
39	SP	CCOR_00715	3.274	0.073	0.420	-4.487	-2.705	0.0038235	0.0275399
40	SP	CCOR_00721	1.024	7.359	6.937	3.427	2.980	9.13E-07	4.03E-16
41	SP	CCOR_00820	468.750	1521.434	993.017	1.804	1.311	6.92E-06	0.0001793
42	SP	CCOR_00822	27.160	21.730	33.670	0.218	1.091	0.4690659	2.26E-05
43	SP	CCOR_00845	0.202	0.754	6.023	--	5.048	--	1.14E-16
44	SP	CCOR_00912	22.744	7.323	18.073	-1.245	-0.106	2.12E-05	0.780593
45	SP	CCOR_01008	6.232	1.117	4.682	-1.908	-0.184	0.0011381	0.6811629
46	SP	CCOR_01011	1.312	1.147	4.448	0.369	1.988	0.5995521	3.01E-06
47	SP	CCOR_01016	9.282	293.315	116.708	5.549	3.880	2.64E-76	1.03E-51
48	SP	CCOR_01055	35.412	366.352	295.835	3.954	3.296	7.79E-16	2.48E-05
49	SP	CCOR_01099	38.143	86.593	128.027	1.754	1.975	1.52E-08	1.62E-17
50	SP	CCOR_01121	18.709	36.302	39.282	1.526	1.299	1.15E-06	8.93E-08
51	SP	CCOR_01176	69.329	107.584	78.155	1.207	0.399	3.37E-05	0.1084216
52	SP	CCOR_01213	475.286	1745.778	1361.818	2.453	1.746	1.28E-11	9.60E-09

53	SP	CCOR_01217	111.594	164.449	217.261	1.170	1.185	0.0004319	3.90E-05
54	SP	CCOR_01302	122.120	745.002	338.603	3.191	1.684	1.66E-15	1.43E-07
55	SP	CCOR_01331	2.425	0.548	0.638	-1.390	-1.651	0.0549112	0.0015717
56	SP	CCOR_01343	49.998	61.020	121.994	0.742	1.523	0.0101943	1.16E-09
57	SP	CCOR_01363	1.623	17.224	39.072	3.935	4.771	1.77E-30	1.29E-77
58	SP	CCOR_01427	5.681	1.172	1.883	-1.705	-1.370	0.0220542	0.0040889
59	SP	CCOR_01446	95.771	197.824	123.126	1.610	0.586	1.17E-07	0.0229777
60	SP	CCOR_01482	72.227	11.611	22.473	-2.078	-1.456	8.63E-14	3.55E-10
61	SP	CCOR_01525	5.741	20.730	10.433	2.426	1.093	7.85E-14	0.0043636
62	SP	CCOR_01562	8.108	20.757	13.640	1.926	0.982	2.34E-05	0.020329
63	SP	CCOR_01564	76.354	110.504	210.522	1.091	1.686	0.0001814	3.13E-10
64	SP	CCOR_01570	45.083	36.367	88.113	0.242	1.195	0.4596331	1.03E-06
65	SP	CCOR_01614	3243.874	9706.858	6996.472	2.124	1.263	0.0005713	0.0046197
66	SP	CCOR_01622	228.010	618.751	647.179	2.021	1.734	1.09E-05	5.04E-13
67	SP	CCOR_01632	49.011	113.925	145.252	1.770	1.798	0.0001001	2.05E-08
68	SP	CCOR_01640	21.517	2.461	5.265	-2.563	-1.800	0.0010055	0.0029643
69	SP	CCOR_01645	112.211	651.501	1292.955	3.111	3.752	1.34E-18	1.00E-11
70	SP	CCOR_01690	132.209	30.643	16.652	-1.525	-2.761	0.0003434	8.41E-11
71	SP	CCOR_01692	1.242	7.476	22.786	3.164	4.415	3.45E-06	0.0008176
72	SP	CCOR_01694	1.459	0.017	0.348	-5.338	-2.178	1.98E-08	1.13E-05
73	SP	CCOR_01697	28.393	69.148	77.272	1.842	1.674	6.70E-10	2.53E-13
74	SP	CCOR_01708	257.363	199.350	73.667	0.205	-1.574	0.7257668	0.0024251
75	SP	CCOR_01726	63.362	105.929	165.353	1.303	1.610	8.01E-06	4.81E-10
76	SP	CCOR_01738	30.310	45.238	43.296	1.151	0.742	0.0001012	0.0021521
77	SP	CCOR_01751	3.851	2.490	14.182	-0.079	2.105	1	1.38E-11
78	SP	CCOR_01769	4.600	183.231	53.947	5.879	3.779	3.77E-82	8.18E-39
79	SP	CCOR_01771	0.220	10.719	2.379	6.152	3.643	1.31E-38	4.24E-15
80	SP	CCOR_01772	0.927	114.140	36.304	8.189	5.615	9.30E-53	1.40E-96

81	SP	CCOR_01777	0.713	0.054	0.308	-2.902	-0.983	0.0001706	0.0571002
82	SP	CCOR_01799	852.240	219.807	808.482	-1.398	0.155	4.62E-05	0.698303
83	SP	CCOR_01810	20.408	486.444	307.277	5.151	4.143	4.22E-30	2.41E-15
84	SP	CCOR_01826	4.391	134.806	89.951	5.513	4.593	6.78E-67	0.0001728
85	SP	CCOR_01840	25.784	406.685	212.065	4.552	3.269	1.27E-46	1.01E-37
86	SP	CCOR_01846	12.416	32.789	82.078	1.950	2.955	0.0013258	2.88E-35
87	SP	CCOR_01848	1.351	3.151	7.897	1.779	2.767	0.014804	1.85E-10
88	SP	CCOR_01875	2.781	1.857	6.347	-0.011	1.419	1	0.0092963
89	SP	CCOR_01876	5.022	1.078	5.644	-1.667	0.397	0.0112829	0.3362728
90	SP	CCOR_01893	4.311	61.755	57.167	4.406	3.959	1.58E-51	4.13E-20
91	SP	CCOR_01897	6.553	26.533	45.569	2.584	3.025	1.04E-16	2.39E-37
92	SP	CCOR_01898	14.520	36.572	48.196	1.899	1.956	3.95E-11	1.36E-16
93	SP	CCOR_01944	54.430	73.293	31.761	1.007	-0.547	0.0057281	0.075513
94	SP	CCOR_02097	2.916	276.750	172.555	7.136	6.112	2.24E-111	1.77E-22
95	SP	CCOR_02125	1.427	9.608	1.677	3.325	0.476	6.97E-08	0.6205184
96	SP	CCOR_02129	32.313	24.669	56.536	0.171	1.034	0.5868328	1.04E-05
97	SP	CCOR_02172	6.145	131.227	119.662	4.977	4.512	1.15E-63	1.14E-48
98	SP	CCOR_02183	13.876	1.376	3.108	-2.734	-1.929	1.31E-05	0.0002051
99	SP	CCOR_02188	9.716	273.800	288.247	5.395	5.121	1.13E-15	1.30E-75
100	SP	CCOR_02201	478.849	251.576	44.322	-0.374	-2.955	0.4763694	4.92E-15
101	SP	CCOR_02204	51.425	15.860	28.619	-1.129	-0.618	0.0002143	0.0136355
102	SP	CCOR_02213	11.355	12.414	48.502	0.676	2.322	0.2123011	2.01E-23
103	SP	CCOR_02333	7.398	8.490	20.091	0.767	1.662	0.293333	0.0002105
104	SP	CCOR_02371	34.546	8.825	18.984	-1.406	-0.638	2.45E-05	0.0143377
105	SP	CCOR_02406	7.993	16.539	10.135	1.620	0.572	8.48E-05	0.1050836
106	SP	CCOR_02522	145.563	401.926	680.986	2.026	2.458	1.80E-12	2.41E-14
107	SP	CCOR_02533	9.770	1.866	4.455	-1.798	-0.903	0.0003786	0.0079934
108	SP	CCOR_02546	40.192	134.281	218.174	2.305	2.669	8.11E-16	6.34E-27

109	SP	CCOR_02657	168.142	3.448	1.154	-5.011	-6.955	8.11E-08	1.82E-12
110	SP	CCOR_02683	165.372	229.027	234.343	1.035	0.731	0.0007225	0.0064129
111	SP	CCOR_02697	331.215	879.721	767.521	1.988	1.439	4.25E-07	1.61E-07
112	SP	CCOR_02775	473.221	1535.612	1039.677	2.275	1.360	3.09E-11	0.000352
113	SP	CCOR_02799	178.553	348.521	363.706	1.536	0.897	9.38E-07	0.0009757
114	SP	CCOR_02804	2.776	118.062	114.865	5.971	5.601	2.34E-62	6.99E-32
115	SP	CCOR_02825	0.087	10.256	2.530	7.330	4.965	5.49E-50	2.74E-23
116	SP	CCOR_02862	156.384	267.809	184.977	1.347	0.471	3.11E-06	0.0632195
117	SP	CCOR_02905	19.065	27.314	31.858	1.074	0.970	0.0031285	0.0004132
118	SP	CCOR_03029	707.953	1183.189	1527.893	1.309	1.339	7.49E-05	1.12E-05
119	SP	CCOR_03036	104.811	0.503	3.496	-7.082	-4.678	6.69E-79	5.73E-56
120	SP	CCOR_03076	3.961	0.801	1.073	-1.650	-1.658	0.0224754	0.0086133
121	SP	CCOR_03097	21.276	47.848	28.472	1.741	0.652	4.43E-09	0.2176693
122	SP	CCOR_03113	4.249	107.240	111.873	5.221	4.943	5.63E-16	3.79E-52
123	SP	CCOR_03121	40.640	174.320	188.791	2.667	2.440	2.91E-15	2.52E-21
124	SP	CCOR_03187	36.939	31.835	70.533	0.344	1.164	0.2552697	7.93E-07
125	SP	CCOR_03348	12.503	36.043	21.789	2.105	1.027	2.49E-09	0.0004225
126	SP	CCOR_03354	73.549	169.169	148.043	1.772	1.236	6.42E-10	8.49E-08
127	SP	CCOR_03421	7.536	2.856	11.602	-0.827	0.850	0.1986708	0.0153462
128	SP	CCOR_03423	12.966	449.439	421.197	5.670	5.249	6.07E-43	6.73E-70
129	SP	CCOR_03438	5.652	29.093	16.947	2.931	1.815	4.61E-19	1.92E-05
130	SP	CCOR_03447	7.452	20.508	28.855	2.051	2.177	0.0333408	1.87E-09
131	SP	CCOR_03460	15.252	5.189	4.860	-1.258	-1.480	0.0002712	2.72E-08
132	SP	CCOR_03462	12.271	27.089	36.691	1.693	1.809	0.0566845	9.20E-07
133	SP	CCOR_03480	3.123	0.604	1.837	-1.821	-0.535	0.0087132	0.2257942
134	SP	CCOR_03504	34.116	4.582	9.195	-2.356	-1.660	3.25E-09	5.89E-09
135	SP	CCOR_03516	3.869	11.319	33.409	2.120	3.329	8.50E-08	4.67E-25
136	SP	CCOR_03523	5.170	28.907	19.127	3.055	2.111	2.04E-12	6.68E-16

137	SP	CCOR_03561	32.437	9.930	18.018	-1.145	-0.622	0.0001651	0.0159686
138	SP	CCOR_03563	33.163	100.921	128.404	2.185	2.183	0.0007308	4.51E-21
139	SP	CCOR_03570	44.744	55.291	187.846	0.856	2.298	0.0236021	8.40E-20
140	SP	CCOR_03572	77.265	1340.720	944.419	4.696	3.844	7.69E-22	8.61E-07
141	SP	CCOR_03575	42.030	70.959	200.025	1.310	2.479	0.0048125	9.17E-24
142	SP	CCOR_03577	8.279	7.129	14.465	0.351	1.036	0.4401049	0.0001436
143	SP	CCOR_03578	21.831	77.209	242.136	2.431	3.588	9.53E-05	1.38E-18
144	SP	CCOR_03584	7.824	41.933	34.355	2.990	2.365	1.65E-20	1.23E-09
145	SP	CCOR_03586	0.270	10.780	48.877	5.859	7.697	9.10E-19	2.38E-51
146	SP	CCOR_03588	19.079	10.487	101.733	-0.298	2.647	0.7448494	5.60E-07
147	SP	CCOR_03593	5.221	6.217	10.655	0.832	1.259	0.162897	0.0002244
148	SP	CCOR_03610	1.470	0.443	0.653	-1.985	-0.931	0.0097243	0.0782325
149	SP	CCOR_03612	82.533	298.264	462.922	2.198	2.502	9.35E-08	1.95E-18
150	SP	CCOR_03617	8.060	1071.999	763.455	7.628	6.778	3.16E-107	4.44E-106
151	SP	CCOR_03618	11.167	632.987	426.610	6.393	5.477	4.98E-86	2.99E-44
152	SP	CCOR_03626	5.691	8.548	7.781	1.163	0.675	0.0087973	0.1896627
153	SP	CCOR_03662	3.535	0.047	2.520	-5.342	-0.263	8.98E-11	0.6023273
154	SP	CCOR_03663	0.050	128.753	69.511	11.756	10.515	6.07E-64	7.03E-189
155	SP	CCOR_03664	3.874	200.747	76.838	5.877	4.198	1.26E-26	4.40E-66
156	SP	CCOR_03668	4.470	0.963	1.241	-1.634	-1.611	0.0136746	0.000137
157	SP	CCOR_03675	160.665	705.377	420.320	2.706	1.619	2.79E-10	1.45E-05
158	SP	CCOR_03678	8.757	65.509	47.298	3.467	2.646	7.53E-34	1.51E-30
159	SP	CCOR_03681	73.714	13.490	23.534	-1.881	-1.422	1.72E-11	8.83E-10
160	SP	CCOR_03684	62.347	23.956	12.950	-0.794	-2.036	0.1363992	0.000256
161	SP	CCOR_03689	8.790	17.389	44.057	1.008	1.745	0.0003762	3.35E-14
162	SP	CCOR_03746	0.000	2.208	0.358	Inf	--	5.95E-12	--
163	SP	CCOR_03749	3599.454	7089.089	2497.067	1.489	-0.360	0.0030169	0.7063424
164	SP	CCOR_03750	13.130	28.284	30.253	1.671	1.436	0.0020131	8.81E-06

165	SP	CCOR_03759	4.854	0.836	1.852	-1.950	-1.154	0.0039256	0.0059459
166	SP	CCOR_03764	27.219	32.665	46.574	0.827	1.002	0.004973	2.26E-05
167	SP	CCOR_03766	23.957	9.879	31.654	-0.709	0.631	0.0633585	0.0183829
168	SP	CCOR_03767	7.577	2.006	9.834	-1.295	0.598	0.0981661	0.215337
169	SP	CCOR_03801	2.436	0.721	0.810	-1.125	-1.345	0.1206241	0.0053479
170	SP	CCOR_03805	8.291	12.847	7.887	1.208	0.159	0.0044156	0.7009259
171	SP	CCOR_03821	7.985	6.705	16.147	0.319	1.245	0.6190741	0.0001448
172	SP	CCOR_03825	15.849	2.514	3.221	-2.062	-2.066	0.0028591	0.0007818
173	SP	CCOR_03846	1.381	0.130	0.696	-2.724	-0.759	0.0001533	0.1003505
174	SP	CCOR_03867	8.409	36.610	13.862	2.686	0.949	6.46E-08	0.0354176
175	SP	CCOR_03910	33.726	13.251	12.608	-0.774	-1.191	0.0327147	9.16E-06
176	SP	CCOR_03915	0.934	26.864	13.867	5.393	4.087	1.28E-29	9.92E-08
177	SP	CCOR_03917	0.980	655.099	456.548	9.954	9.091	1.27E-153	7.66E-46
178	SP	CCOR_03918	10.968	931.712	469.909	6.987	5.653	4.81E-54	1.21E-20
179	SP	CCOR_03929	7.376	24.029	34.102	2.285	2.439	0.0006045	5.56E-08
180	SP	CCOR_03930	2.994	0.908	3.998	-1.113	0.643	0.0698143	0.0818841
181	SP	CCOR_03954	7.862	151.287	141.099	4.837	4.394	3.72E-33	7.99E-38
182	SP	CCOR_03964	46.564	319.268	1004.761	3.340	4.666	1.15E-24	5.15E-12
183	SP	CCOR_03966	30.033	73.814	135.978	1.859	2.407	1.06E-10	1.51E-23
184	SP	CCOR_04017	1011.263	2754.759	1677.336	2.026	0.957	1.42E-07	0.0023757
185	SP	CCOR_04022	16.283	0.488	1.346	-4.420	-3.366	2.15E-16	1.77E-21
186	SP	CCOR_04035	14.027	21.393	28.389	1.165	1.240	3.19E-05	1.92E-06
187	SP	CCOR_04047	21.222	4.343	15.180	-1.725	-0.256	2.49E-05	0.4393995
188	SP	CCOR_04058	1.406	0.014	0.180	-5.230	-2.692	2.50E-07	2.56E-07
189	SP	CCOR_04113	46.271	84.972	53.814	1.459	0.445	0.0003108	0.0923644
190	SP	CCOR_04164	19.087	8.853	1.610	-0.517	-3.316	0.4724717	0.0003704
191	SP	CCOR_04229	378.614	1693.758	1095.890	2.728	1.763	4.84E-14	9.82E-08
192	SP	CCOR_04255	323.451	555.047	429.457	1.343	0.637	6.74E-06	0.0173277

193	SP	CCOR_04302	7.190	59.344	31.726	3.619	2.368	9.32E-26	5.23E-20
194	SP	CCOR_04327	0.359	5.726	17.886	4.526	5.824	2.50E-08	5.08E-50
195	SP	CCOR_04332	18.491	50.507	31.541	2.024	1.005	2.58E-09	0.1921141
196	SP	CCOR_04333	387.033	2456.957	2158.766	3.253	2.711	9.25E-06	7.94E-06
197	SP	CCOR_04337	8.971	6.155	22.533	0.013	1.556	0.8826649	8.55E-10
198	SP	CCOR_04347	3.706	10.304	9.324	2.043	1.560	1.39E-05	0.0004676
199	SP	CCOR_04359	25.561	85.763	195.910	2.331	3.165	0.0006741	4.26E-14
200	SP	CCOR_04380	2.627	5.006	6.459	1.518	1.521	0.0665829	0.0005062
201	SP	CCOR_04381	1.608	4.748	7.018	2.135	2.356	0.0020201	0.0003678
202	SP	CCOR_04454	5.795	0.708	1.082	-2.435	-2.186	1.30E-05	3.02E-09
203	SP	CCOR_04513	25.498	26.220	63.392	0.601	1.541	0.0421155	1.22E-11
204	SP	CCOR_04520	39.350	140.510	172.006	2.397	2.358	4.02E-16	6.95E-20
205	SP	CCOR_04547	0.136	4.264	1.454	5.413	--	2.10E-11	--
206	SP	CCOR_04556	256.105	643.527	741.393	1.886	1.757	1.13E-08	0.0001717
207	SP	CCOR_04634	41.297	20.168	58.127	-0.473	0.720	0.1339552	0.0028946
208	SP	CCOR_04635	31.030	14.115	42.858	-0.585	0.694	0.0735662	0.0040261
209	SP	CCOR_04644	148.435	246.323	263.975	1.298	1.062	1.71E-05	0.0012193
210	SP	CCOR_04669	108.721	43.283	121.670	-0.755	0.393	0.0060045	0.160962
211	SP	CCOR_04703	8.615	0.665	2.477	-3.021	-1.558	1.01E-05	0.0001584
212	SP	CCOR_04710	0.450	174.022	72.597	9.130	7.523	1.20E-33	6.87E-11
213	SP	CCOR_04711	37.127	52.966	31.684	1.086	0.000	0.0017213	0.987332
214	SP	CCOR_04712	43.172	59.226	33.792	1.031	-0.123	0.0005749	0.6538043
215	SP	CCOR_04734	148.766	1408.424	1172.454	3.807	3.213	1.39E-10	7.33E-21
216	SP	CCOR_04741	1.340	471.628	192.749	8.916	7.295	1.99E-73	6.56E-21
217	SP	CCOR_04826	3.188	78.274	150.569	5.180	5.790	3.94E-48	8.76E-95
218	SP	CCOR_04861	0.114	16.727	21.297	7.543	7.564	2.63E-26	4.01E-47
219	SP	CCOR_04883	33.286	10.602	12.325	-1.085	-1.205	0.0014825	2.06E-06
220	SP	CCOR_04885	15.089	15.773	49.459	0.638	1.945	0.1082809	2.12E-05

221	SP	CCOR_04892	1.416	0.249	0.361	-1.852	-1.741	0.0143041	0.0003821
222	SP	CCOR_04893	0.223	50.324	14.819	8.281	6.166	1.33E-22	3.58E-17
223	SP	CCOR_04909	56.245	197.498	264.586	2.373	2.463	2.84E-16	3.21E-22
224	SP	CCOR_04915	38.651	164.184	178.399	2.645	2.437	5.35E-21	2.74E-17
225	SP	CCOR_04946	31.584	72.032	102.098	1.754	1.926	0.0004698	0.0001549
226	SP	CCOR_04948	94.589	10.260	44.184	-2.614	-0.883	1.21E-22	0.0001752
227	SP	CCOR_04977	2.156	5.183	14.253	1.826	2.949	0.000197	1.36E-26
228	SP	CCOR_04980	3.534	35.648	3.393	3.899	0.177	3.40E-10	0.8963026
229	SP	CCOR_04988	6.884	79.811	182.856	4.100	4.961	2.16E-34	1.29E-77
230	SP	CCOR_04989	121.526	39.262	96.354	-1.071	-0.111	0.0001569	0.7951055
231	SP	CCOR_04995	4.080	5.516	8.675	1.001	1.315	0.051996	4.93E-05
232	SP	CCOR_04996	4.922	0.437	1.218	-2.852	-1.842	2.75E-06	2.77E-06
233	SP	CCOR_04998	20.858	38.055	54.718	1.431	1.621	8.15E-07	2.28E-12
234	SP	CCOR_05000	16.396	43.899	58.004	1.964	2.054	0.003938	2.86E-05
235	SP	CCOR_05002	5.359	0.134	0.742	-4.528	-2.621	1.50E-09	2.99E-09
236	SP	CCOR_05122	2.885	0.113	0.766	-3.911	-1.667	2.46E-06	0.0009577
237	SP	CCOR_05127	0.941	56.682	45.879	6.457	5.816	7.45E-59	1.22E-16
238	SP	CCOR_05129	2.347	7.923	21.647	2.310	3.433	4.50E-06	1.01E-43
239	SP	CCOR_05135	329.209	83.716	75.549	-1.397	-1.896	2.15E-07	3.60E-14
240	SP	CCOR_05153	2.693	2.006	5.569	0.171	1.271	0.8903895	0.0005573
241	SP	CCOR_05163	0.452	131.645	47.583	8.742	6.927	1.27E-68	1.70E-123
242	SP	CCOR_05165	3.835	9.043	11.636	1.834	1.888	0.1200752	0.0055876
243	SP	CCOR_05171	2.739	107.569	57.765	5.866	4.623	1.49E-39	1.38E-18
244	SP	CCOR_05183	7.849	428.978	315.723	6.338	5.559	2.76E-38	1.28E-96
245	SP	CCOR_05189	0.086	3.292	5.800	--	6.211	--	1.29E-32
246	SP	CCOR_05191	0.103	0.746	1.395	--	3.778	--	1.12E-11
247	SP	CCOR_05192	0.325	36.243	17.846	7.323	5.973	1.32E-79	1.61E-18
248	SP	CCOR_05199	1.925	86.304	55.280	5.313	4.470	5.04E-70	8.62E-09

249	SP	CCOR_05200	5.122	19.342	31.960	2.485	2.870	5.17E-10	5.97E-26
250	SP	CCOR_05239	0.332	10.386	5.610	5.486	4.243	2.18E-14	2.82E-15
251	SP	CCOR_05314	3.050	12.087	10.248	2.557	1.974	4.03E-07	4.79E-08
252	SP	CCOR_05326	7.225	38.928	29.994	3.010	2.283	2.47E-06	5.23E-19
253	SP	CCOR_05356	5.966	53.651	38.287	3.733	2.911	1.25E-34	1.37E-15
254	SP	CCOR_05364	6.167	21.656	16.453	2.306	1.649	9.02E-05	6.09E-12
255	SP	CCOR_05391	169.736	46.650	62.318	-1.296	-1.218	1.68E-06	9.56E-07
256	SP	CCOR_05409	14.647	210.314	68.355	5.226	3.254	1.23E-63	1.87E-38
257	SP	CCOR_05411	13.554	84.032	50.154	3.206	2.120	1.40E-25	1.66E-05
258	SP	CCOR_05413	40.717	21.863	62.835	-0.342	0.854	0.3594397	0.0002496
259	SP	CCOR_05425	5.193	19.288	8.231	2.474	0.891	9.14E-07	0.0288659
260	SP	CCOR_05449	34.930	50.993	44.166	1.116	0.566	0.0001382	0.0209128
261	SP	CCOR_05450	124.767	391.492	369.535	2.215	1.762	3.32E-13	7.16E-08
262	SP	CCOR_05495	56.247	113.729	187.645	1.587	1.973	0.0125058	0.0017541
263	SP	CCOR_05499	2.806	6.691	6.171	1.813	1.363	0.0192701	0.0002906
264	SP	CCOR_05536	510.800	985.824	655.916	1.525	0.588	5.12E-05	0.0829968
265	SP	CCOR_05581	17.527	12.375	32.763	0.071	1.130	0.9072331	2.83E-06
266	SP	CCOR_05587	29.052	36.558	41.052	0.875	1.001	0.0026003	1.11E-05
267	SP	CCOR_05592	90.596	24.113	43.120	-1.425	-0.737	5.11E-05	0.0093403
268	SP	CCOR_05622	172.847	67.063	238.510	-0.805	0.691	0.0070609	0.0197927
269	SP	CCOR_05681	14.053	1.914	5.651	-2.287	-1.084	9.83E-08	0.0001712
270	SP	CCOR_05742	26.529	12.375	36.702	-0.528	0.699	0.13842	0.0066156
271	SP	CCOR_05769	8.885	26.065	29.654	2.120	1.966	9.00E-11	8.77E-16
272	SP	CCOR_05781	274.638	515.401	359.287	1.487	0.613	0.0013208	0.0208085
273	SP	CCOR_05787	33.790	77.265	54.967	1.764	0.930	8.41E-10	7.82E-05
274	SP	CCOR_05819	129.210	272.221	223.723	1.643	1.017	1.47E-08	1.94E-05
275	SP	CCOR_05890	78.888	286.636	441.534	2.429	2.715	8.11E-16	2.26E-15
276	SP	CCOR_05950	42.159	91.936	16.400	1.708	-1.130	0.0002844	0.0002825

277	SP	CCOR_05992	66.393	283.431	106.362	2.671	0.908	2.31E-16	0.0001066
278	SP	CCOR_05998	36.180	48.701	64.945	0.999	1.075	0.0009596	3.79E-05
279	SP	CCOR_06008	76.589	150.070	134.006	1.539	1.037	1.11E-07	1.15E-05
280	SP	CCOR_06026	7.478	2.472	9.996	-0.999	0.646	0.0409781	0.0290269
281	SP	CCOR_06055	1.492	12.937	18.071	3.656	3.824	6.82E-05	3.35E-07
282	SP	CCOR_06150	36.918	16.658	38.819	-0.716	0.261	0.0163428	0.2968535
283	SP	CCOR_06165	55.319	386.589	191.662	3.373	2.021	1.20E-30	4.42E-16
284	SP	CCOR_06181	121.316	37.457	62.841	-1.104	-0.728	5.61E-05	0.0032542
285	SP	CCOR_06188	117.373	409.874	621.759	2.375	2.639	6.88E-16	4.84E-11
286	SP	CCOR_06200	3076.921	8875.089	3832.091	2.107	0.543	0.0005404	0.2062986
287	SP	CCOR_06230	12.507	6.516	18.311	-0.389	0.775	0.3479024	0.0009479
288	SP	CCOR_06282	2.271	19.468	79.120	3.668	5.346	1.34E-16	2.13E-78
289	SP	CCOR_06295	6.285	4.490	20.317	0.122	1.924	0.9455593	0.0072181
290	SP	CCOR_06299	38.909	17.035	51.634	-0.604	0.637	0.1619644	0.0282804
291	SP	CCOR_06337	119.356	308.119	372.660	1.939	1.870	4.52E-11	5.66E-14
292	SP	CCOR_06350	2.645	0.169	1.180	-3.190	-0.938	3.65E-06	0.0391453
293	SP	CCOR_06358	51.540	393.595	458.806	3.501	3.384	9.57E-34	2.40E-19
294	SP	CCOR_06395	43.180	134.444	61.579	2.220	0.743	6.51E-05	0.0022012
295	SP	CCOR_06398	9.243	14.183	23.188	1.183	1.555	0.0859728	1.64E-10
296	SP	CCOR_06418	13.370	153.416	55.665	4.095	2.288	9.93E-25	1.51E-10
297	SP	CCOR_06452	2.128	31.283	33.451	4.453	4.201	1.01E-21	3.39E-59
298	SP	CCOR_06458	55.736	381.021	999.867	3.145	4.182	3.54E-23	2.27E-32
299	SP	CCOR_06459	6.332	2.006	7.622	-1.081	0.496	0.0329276	0.1261243
300	SP	CCOR_06464	10.188	8.576	18.031	0.310	1.053	0.367314	1.91E-05
301	SP	CCOR_06535	8.874	1559.978	428.504	8.037	5.820	6.12E-20	2.99E-98
302	SP	CCOR_06549	3.340	8.520	18.277	1.925	2.678	0.0001304	2.30E-20
303	SP	CCOR_06556	20.503	46.278	48.613	1.739	1.472	1.48E-09	1.41E-10
304	SP	CCOR_06624	596.199	140.673	121.679	-1.513	-2.065	3.46E-07	5.12E-13

305	SP	CCOR_06626	0.803	0.079	0.351	-2.349	-0.960	0.0015429	0.0674727
306	SP	CCOR_06659	2.553	0.137	3.009	--	0.483	--	0.8259415
307	SP	CCOR_06713	13.293	13.853	70.896	0.637	2.639	0.1989881	6.78E-27
308	SP	CCOR_06714	20.619	25.964	111.202	0.057	2.038	0.9069486	9.64E-18
309	SP	CCOR_06772	54.730	59.005	21.009	0.700	-1.153	0.3411174	0.0216568
310	SP	CCOR_06799	59.263	8.689	9.961	-2.189	-2.343	1.51E-11	6.44E-16
311	SP	CCOR_06801	19.359	31.959	62.148	1.287	1.910	1.22E-05	8.14E-17
312	SP	CCOR_06815	18.979	4.862	39.224	-1.398	1.274	0.0002165	7.32E-08
313	SP	CCOR_06827	3.285	0.333	0.871	-2.641	-1.679	0.0001043	0.0001278
314	SP	CCOR_06848	1.108	0.109	0.891	-2.370	-0.093	0.0017213	0.903882
315	SP	CCOR_06869	0.000	0.132	1.021	--	Inf	--	9.75E-21
316	SP	CCOR_06874	0.044	14.439	5.487	8.728	7.100	2.50E-08	7.30E-24
317	SP	CCOR_06961	31.357	51.981	91.784	1.306	1.728	4.81E-06	2.22E-12
318	SP	CCOR_06963	1.666	4.989	32.178	1.759	3.660	0.0003855	4.82E-46
319	SP	CCOR_06967	11.414	18.776	22.205	1.051	1.097	0.0002717	2.27E-05
320	SP	CCOR_06973	613.752	207.197	108.111	-1.077	-1.648	0.0063731	6.12E-05
321	SP	CCOR_06974	13.437	63.362	3.354	2.759	-1.788	0.049844	0.6346069
322	SP	CCOR_06988	4.342	5.741	9.734	0.976	1.389	0.0625518	7.74E-06
323	SP	CCOR_06996	0.587	47.458	171.819	6.902	8.406	2.18E-07	2.41E-28
324	SP	CCOR_07050	335.556	810.964	690.551	1.873	1.268	9.30E-08	3.50E-05
325	SP	CCOR_07055	4.500	60.352	73.785	4.307	4.263	2.71E-40	1.08E-63
326	SP	CCOR_07117	3.406	2.346	3.639	-0.622	0.362	0.1406534	0.2203021
327	SP	CCOR_07213	1245.294	1823.139	807.257	1.115	-0.399	0.0005163	0.1961799
328	SP	CCOR_07219	4.634	0.429	0.538	-2.806	-2.862	6.57E-07	1.71E-13
329	SP	CCOR_07220	51.169	58.929	14.394	-0.677	-2.556	0.0134689	4.58E-28
330	SP	CCOR_07278	20.728	198.557	284.223	3.089	2.847	8.51E-24	1.13E-25
331	SP	CCOR_07280	14.183	188.213	58.587	4.310	2.274	7.37E-18	4.23E-23
332	SP	CCOR_07296	27.376	51.950	35.086	1.391	0.589	2.35E-06	0.0175206

333	SP	CCOR_07409	27.173	5.338	26.546	-1.789	0.194	7.41E-08	0.4963287
334	SP	CCOR_07451	1.113	0.114	0.269	-2.623	-1.824	0.0026014	0.0025431
335	SP	CCOR_07490	16.070	5.984	6.263	-0.862	-1.133	0.0522322	0.0001318
336	SP	CCOR_07537	2.741	0.060	0.218	-4.694	-3.344	1.28E-06	3.40E-09
337	SP	CCOR_07538	0.178	48.940	33.881	8.546	7.668	6.34E-22	8.30E-12
338	SP	CCOR_07550	5.446	32.515	6.409	3.168	0.468	0.0093562	0.2930623
339	SP	CCOR_07551	0.174	38.526	6.869	8.251	5.399	9.86E-08	2.88E-20
340	SP	CCOR_07575	8.106	0.067	0.962	-6.378	-2.848	1.28E-11	8.28E-06
341	SP	CCOR_07632	1.426	8.168	15.908	3.072	3.700	8.07E-09	4.46E-30
342	SP	CCOR_07637	560.643	443.309	1333.431	0.250	1.472	0.4853195	9.29E-07
343	SP	CCOR_07800	4.101	148.901	87.285	5.753	4.638	1.16E-53	3.30E-21
344	SP	CCOR_07831	6.099	1.125	1.630	-1.857	-1.676	0.001125	8.08E-06
345	SP	CCOR_07832	7.847	11.914	7.197	1.179	0.101	0.0002829	0.7257707
346	SP	CCOR_07837	2.759	7.654	3.119	2.092	--	0.0071458	--
347	SP	CCOR_07840	0.000	2.726	0.230	Inf	--	2.79E-11	--
348	SP	CCOR_07845	2.972	0.583	0.904	-1.624	-1.449	0.0326414	0.0075006
349	SP	CCOR_07851	1.247	2445.425	2508.521	11.492	11.184	3.33E-105	3.89E-156
350	SP	CCOR_07852	15.234	401.408	448.398	5.286	5.108	6.82E-62	1.01E-40
351	SP	CCOR_07853	14.997	403.095	225.958	5.316	4.139	1.28E-71	3.53E-55
352	SP	CCOR_07855	0.024	55.991	22.990	11.328	9.683	1.52E-93	1.35E-67
353	SP	CCOR_07857	1.742	0.054	0.131	-3.883	-3.379	7.68E-06	3.32E-09
354	SP	CCOR_07859	5.586	0.643	1.301	-2.543	-1.863	0.000664	0.0008069
355	SP	CCOR_07907	22.641	0.572	1.728	-4.650	-3.477	1.57E-16	1.73E-21
356	SP	CCOR_07915	503.288	1271.431	1188.570	1.898	1.470	1.08E-07	6.31E-06
357	SP	CCOR_07937	24.291	60.452	87.860	1.869	2.083	7.75E-07	1.84E-19
358	SP	CCOR_07966	48.181	15.550	60.571	-1.059	0.557	0.0001881	0.0191318
359	SP	CCOR_08009	1899.540	7.272	164.624	-7.460	-3.298	7.63E-65	2.56E-25
360	SP	CCOR_08033	3.739	1.016	0.520	-1.285	-2.607	0.0202829	6.24E-07

361	SP	CCOR_08034	2.006	1.317	0.625	-0.002	-1.443	0.982434	0.0038467
362	SP	CCOR_08054	30.079	31.664	17.172	0.641	-0.576	0.080374	0.075876
363	SP	CCOR_08063	35.015	49.117	118.517	0.971	1.984	0.0008223	2.75E-17
364	SP	CCOR_08070	1.212	41.188	23.220	5.645	4.476	6.73E-44	6.87E-34
365	SP	CCOR_08079	13.125	3.878	5.178	-1.165	-1.111	0.0319514	0.0026133
366	SP	CCOR_08085	0.787	0.000	0.042	--	-3.922	1.74E-08	5.09E-11
367	SP	CCOR_08094	9.867	2.737	3.783	-1.277	-1.153	0.0198476	0.0011328
368	SP	CCOR_08103	8.645	316.568	141.520	5.769	4.257	6.40E-16	9.68E-25
369	SP	CCOR_08106	81.125	172.707	247.388	1.667	1.838	5.36E-05	1.61E-15
370	SP	CCOR_08126	1.091	38.408	9.766	5.638	3.325	1.78E-40	0.0001822
371	SP	CCOR_08127	0.046	11.447	1.587	8.111	--	1.50E-11	--
372	SP	CCOR_08135	0.111	58.846	3.289	9.501	4.993	9.77E-59	9.77E-07
373	SP	CCOR_08140	1.328	15.850	5.537	4.148	2.290	3.28E-07	1.48E-07
374	SP	CCOR_08148	2.542	10.203	14.561	2.441	2.870	3.96E-06	3.85E-07
375	SP	CCOR_08158	2.174	0.533	0.487	-1.980	-1.821	0.0135457	0.0003415
376	SP	CCOR_08163	4.215	0.255	4.400	--	0.293	--	0.8042888
377	SP	CCOR_08186	23.531	5.834	7.301	-1.438	-1.464	0.0002065	2.58E-07
378	SP	CCOR_08189	20.047	13.454	80.466	0.246	2.156	0.5080307	1.28E-10
379	SP	CCOR_08194	16.218	13.648	66.258	0.294	2.259	0.5434462	1.21E-22
380	SP	CCOR_08199	1.959	22.999	15.090	3.948	2.997	2.85E-35	2.65E-28
381	SP	CCOR_08201	2.700	7.805	23.451	2.082	3.336	0.0030472	3.12E-07
382	SP	CCOR_08207	0.709	0.110	0.134	-1.831	-2.143	0.0143784	7.73E-05
383	SP	CCOR_08208	84.368	0.279	5.236	-7.612	-3.806	6.37E-21	2.12E-10
384	SP	CCOR_08211	0.890	3.419	3.657	2.507	2.259	0.0001941	2.97E-07
385	SP	CCOR_08213	0.942	1.779	3.854	1.571	2.283	0.0378612	3.72E-06
386	SP	CCOR_08218	1.521	7.750	9.345	2.941	2.840	0.0032293	2.91E-13
387	SP	CCOR_08230	0.047	0.165	1.291	--	4.963	--	8.89E-15
388	SP	CCOR_08231	5.621	5.647	11.092	0.991	1.170	0.0171102	0.0011476

389	SP	CCOR_08236	1.903	0.154	1.212	-2.904	-0.424	4.29E-05	0.4585789
390	SP	CCOR_08237	6.555	170.517	98.749	5.271	4.144	2.20E-67	3.25E-28
391	SP	CCOR_08239	0.036	32.899	13.651	10.249	8.638	2.03E-151	8.26E-109
392	SP	CCOR_08245	13.521	26.711	47.369	1.068	1.585	0.012669	6.06E-07
393	SP	CCOR_08294	0.784	162.205	25.852	8.259	5.257	4.05E-35	3.98E-60
394	SP	CCOR_08298	0.389	335.428	66.213	10.311	7.616	1.67E-35	5.99E-143
395	SP	CCOR_08352	11.823	136.770	282.867	4.093	4.811	6.71E-20	7.05E-23
396	SP	CCOR_08418	62.637	87.510	61.836	1.054	0.209	0.0002882	0.4344472
397	SP	CCOR_08465	0.021	165.349	12.397	12.956	8.875	7.05E-41	4.47E-15
398	SP	CCOR_08469	1.606	20.815	8.002	4.261	2.533	3.71E-19	9.40E-10
399	SP	CCOR_08470	21.726	37.888	57.564	1.366	1.635	3.03E-06	1.46E-12
400	SP	CCOR_08486	0.559	10.457	4.898	4.803	3.352	5.45E-26	1.09E-06
401	SP	CCOR_08494	5.781	1.668	1.560	-1.201	-1.660	0.0984358	0.0001992
402	SP	CCOR_08504	31.741	101.625	230.002	2.244	3.084	7.90E-15	7.33E-31
403	SP	CCOR_08527	50.397	20.643	16.206	-0.743	-1.418	0.0060368	4.73E-07
404	SP	CCOR_08553	0.722	3.154	1.554	2.708	1.320	0.0001952	0.0114087
405	SP	CCOR_08555	21.994	8.624	7.357	-0.770	-1.348	0.0467232	1.65E-06
406	SP	CCOR_08561	9.035	2.354	3.028	-1.330	-1.342	0.0301173	0.0012006
407	SP	CCOR_08565	14.241	3.118	4.576	-1.598	-1.408	0.0105242	0.0006516
408	SP	CCOR_08632	1.252	0.143	0.687	-2.416	-0.626	0.0013757	0.199205
409	SP	CCOR_08653	15.607	21.876	23.387	1.045	0.810	0.0004724	0.0008326
410	SP	CCOR_08654	1.156	0.160	0.391	-2.079	-1.319	0.0062619	0.0163206
411	SP	CCOR_08740	99.849	225.149	137.358	1.718	0.647	6.92E-09	0.0073826
412	SP	CCOR_08811	143.105	306.850	445.232	1.654	1.872	0.0086105	0.0001306
413	SP	CCOR_08831	6.613	34.147	53.139	2.932	3.234	3.40E-24	3.37E-40
414	SP	CCOR_08953	10.984	2.560	5.091	-1.513	-0.881	0.0093096	0.0249115
415	SP	CCOR_09028	46.599	51.889	324.480	0.712	3.030	0.141403	1.84E-30
416	SP	CCOR_09065	171.919	217.714	113.448	0.916	-0.372	0.0023716	0.1589686

417	SP	CCOR_09093	0.936	46.604	17.189	6.184	4.411	1.28E-64	4.17E-17
418	SP	CCOR_09101	26.351	29.288	81.656	0.710	1.859	0.0128972	1.18E-15
419	SP	CCOR_09102	1.118	0.629	2.680	-0.234	1.488	0.8574822	0.0006696
420	SP	CCOR_09141	0.000	0.000	1.178	--	Inf	--	1.92E-19
421	SP	CCOR_09144	24.806	4.477	6.080	-1.890	-1.802	0.0011641	0.00043
422	SP	CCOR_09166	10.704	21.017	19.594	1.541	1.099	1.21E-07	3.16E-06
423	SP	CCOR_09203	12.515	25.208	27.709	1.573	1.379	6.69E-06	0.0152683
424	SP	CCOR_09224	15.255	1.988	16.034	-2.430	0.299	7.34E-06	0.3588495
425	SP	CCOR_09229	8.395	38.066	100.356	2.746	3.808	1.70E-20	3.35E-54
426	SP	CCOR_09238	60.320	103.832	501.162	1.359	3.293	1.16E-05	0.0036772
427	SP	CCOR_09255	1.434	15.003	24.341	3.946	4.308	3.34E-12	6.17E-13
428	SP	CCOR_09273	1.190	3.844	1.877	2.273	0.881	0.0010517	0.0515679
429	SP	CCOR_09276	34.804	31.492	8.408	0.436	-1.816	0.4113397	2.88E-07
430	SP	CCOR_09281	1.690	0.239	0.867	-2.132	-0.728	0.0040419	0.1418062
431	SP	CCOR_09326	29.319	114.082	33.925	2.533	0.441	6.77E-18	0.1449638
432	SP	CCOR_09334	47.879	0.769	1.519	-5.290	-4.733	6.35E-11	4.24E-11
433	SP	CCOR_09335	129.326	0.707	1.416	-7.065	-6.338	1.20E-11	2.99E-11
434	SP	CCOR_09338	8.290	3.001	2.362	-0.895	-1.577	0.1500499	4.61E-05
435	SP	CCOR_09347	0.043	12.052	34.684	8.281	9.469	3.62E-41	3.00E-123
436	SP	CCOR_09355	27.314	135.668	180.130	2.888	2.950	3.40E-20	2.89E-36
437	SP	CCOR_09359	3.326	40.506	29.860	4.191	3.388	2.63E-06	6.01E-13
438	SP	CCOR_09360	1.530	332.400	81.134	8.326	5.942	3.31E-22	1.12E-83
439	SP	CCOR_09365	4.765	1.338	1.205	-1.183	-1.741	0.0748135	0.0002232
440	SP	CCOR_09367	0.655	59.251	34.652	7.059	5.938	1.36E-52	1.45E-27
441	SP	CCOR_09388	0.016	31.056	5.499	10.958	8.115	2.00E-24	6.31E-08
442	SP	CCOR_09396	0.680	0.013	0.420	-4.471	-0.465	3.08E-07	0.4079938
443	SP	CCOR_09409	0.090	14.549	6.970	7.562	6.148	2.26E-09	1.68E-13
444	SP	CCOR_09419	0.265	1.377	1.649	--	2.713	--	4.26E-05

445	SP	CCOR_09423	40.940	247.680	296.577	3.157	3.085	6.60E-26	7.93E-31
446	SP	CCOR_09426	14.729	15.172	41.691	0.603	1.731	0.1911912	1.03E-10
447	SP	CCOR_09447	144.324	1321.358	776.837	3.778	2.656	1.32E-07	1.51E-07
448	SP	CCOR_09506	3.125	5.053	32.349	0.755	3.240	0.080374	1.28E-42
449	SP	CCOR_09540	0.818	1.857	2.128	1.778	1.606	0.0225989	0.0013018
450	SP	CCOR_09544	10.467	39.119	24.492	2.472	1.453	1.37E-17	5.28E-10
451	SP	CCOR_09563	21.031	33.873	39.258	1.258	1.134	0.0002003	0.0219566
452	SP	CCOR_09566	9.773	5.104	15.528	-0.373	0.895	0.4595344	0.0007679
453	SP	CCOR_09587	0.832	0.131	0.109	-1.902	-2.611	0.0108077	7.03E-07
454	SP	CCOR_09595	6.104	1.462	9.178	-1.465	0.814	0.0257418	0.0319783
455	SP	CCOR_09596	1.507	8.737	69.159	3.108	5.744	4.17E-09	1.89E-78
456	SP	CCOR_09616	41.640	7.532	11.536	-1.892	-1.627	1.51E-07	1.25E-09
457	SP	CCOR_09626	0.863	3.657	2.102	2.669	1.518	0.00028	0.0110962
458	SP	CCOR_09658	2.275	4.779	14.952	1.605	2.936	0.1849725	2.42E-15
459	SP	CCOR_09737	1.437	0.333	2.109	--	0.770	--	0.1634436
460	SP	CCOR_09859	215.971	377.564	311.157	1.375	0.757	8.81E-06	0.0061559
461	SP	CCOR_09960	108.175	9.732	15.368	-2.701	-2.457	2.18E-11	5.48E-12
462	SP	CCOR_09990	91.081	188.765	126.516	1.626	0.702	2.07E-08	0.0034465
463	SP	CCOR_09993	5.073	0.161	1.664	-4.349	-1.374	2.17E-13	6.15E-05
464	SP	CCOR_09998	3.823	8.747	0.266	1.769	--	0.0003484	--
465	SP	CCOR_10068	0.338	278.606	158.236	10.178	9.013	5.27E-41	1.50E-14
466	SP	CCOR_10110	8.406	104.214	116.488	4.198	4.025	6.86E-46	9.26E-14
467	SP	CCOR_10141	0.602	3.374	9.302	3.029	4.147	0.0005897	7.39E-05
468	SP	CCOR_10155	1.836	22.350	36.903	4.177	4.557	8.70E-22	2.24E-53
469	SP	CCOR_10160	32.664	49.929	40.393	1.182	0.536	0.0001338	0.0488648
470	SP	CCOR_10165	5.024	1.052	2.870	-1.671	-0.579	0.0060368	0.1613574
471	SP	CCOR_10176	42.177	105.587	34.697	1.901	-0.053	0.0001606	0.9470538
472	SP	CCOR_10188	43.513	6.442	25.131	-2.199	-0.565	2.68E-11	0.0250752

473	SP	CCOR_10191	8.957	49.981	27.178	3.063	1.808	2.07E-06	9.24E-15
474	SP	CCOR_10205	2.797	139.015	85.173	6.205	5.154	1.55E-72	1.06E-39
475	SP	CCOR_10213	4.970	157.623	111.779	5.561	4.718	1.31E-25	2.18E-23
476	SP	CCOR_10220	54.104	4.340	9.314	-3.039	-2.311	4.95E-09	1.50E-07
477	SP	CCOR_10231	117.244	397.574	309.116	2.344	1.629	9.73E-06	3.25E-06
478	SP	CCOR_10256	16.394	132.388	143.437	3.580	3.356	2.44E-35	1.10E-42
479	SP	CCOR_10299	8.355	3.036	13.003	-0.912	0.871	0.0507055	0.0604128
480	SP	CCOR_10304	0.673	133.798	50.610	8.128	6.377	9.62E-38	3.88E-13
481	SP	CCOR_10305	77.332	92.449	134.024	0.813	1.018	0.0270327	0.0036755
482	SP	CCOR_10310	4.172	0.634	0.980	-2.109	-1.863	0.0066536	0.000265
483	SP	CCOR_10322	0.000	45.384	21.134	Inf	Inf	7.33E-20	4.05E-14
484	SP	CCOR_10325	4.307	11.288	39.140	1.962	3.411	1.35E-05	3.11E-42
485	SP	CCOR_10339	2.074	2.227	11.278	0.648	2.671	0.25466	7.20E-24
486	SP	CCOR_10348	16.449	453.175	385.163	5.352	4.782	2.77E-30	8.39E-36
487	SP	CCOR_10352	3.757	1.026	1.058	-1.268	-1.598	0.0260182	4.15E-05
488	SP	CCOR_10360	9.881	21.850	47.749	1.713	2.503	0.0005361	1.50E-17
489	SP	CCOR_10361	0.882	16.863	292.952	4.786	8.581	6.27E-09	2.19E-74
490	SP	CCOR_10363	43.332	110.057	250.637	1.928	2.760	3.64E-05	5.73E-32
491	SP	CCOR_10376	6.486	0.851	1.225	-2.365	-2.171	0.0048844	0.0005167
492	SP	CCOR_10381	0.191	18.002	16.560	7.103	6.637	6.13E-41	2.50E-75
493	SP	CCOR_10386	0.469	1.381	1.985	2.125	2.286	0.0053384	2.31E-06
494	SP	CCOR_10391	3.184	0.371	0.726	-2.444	-1.878	3.21E-05	1.36E-06
495	SP	CCOR_10392	258.013	760.238	539.199	2.133	1.290	1.79E-12	1.74E-07
496	SP	CCOR_10400	25.171	6.834	31.768	-1.312	0.565	0.0157022	0.0567708
497	SP	CCOR_10440	0.305128	152.0389	86.774157	9.499429	8.356042	2.91E-156	3.80E-45
1	effector	CCOR_10322	0.000	45.384	21.134	Inf	Inf	7.33E-20	4.05E-14
2	effector	CCOR_09141	0.000	0.000	1.178	--	Inf	--	1.92E-19
3	effector	CCOR_07840	0.000	2.726	0.230	Inf	--	2.79E-11	--

4	effector	CCOR_04547	0.136	4.264	1.454	5.413	--	2.10E-11	--
5	effector	CCOR_07837	2.759	7.654	3.119	2.092	--	0.0071458	--
6	effector	CCOR_07851	1.247	2445.425	2508.521	11.492	11.184	3.33E-105	3.89E-156
7	effector	CCOR_10068	0.338	278.606	158.236	10.178	9.013	5.27E-41	1.50E-14
8	effector	CCOR_08465	0.021	165.349	12.397	12.956	8.875	7.05E-41	4.47E-15
9	effector	CCOR_10361	0.882	16.863	292.952	4.786	8.581	6.27E-09	2.19E-74
10	effector	CCOR_09388	0.016	31.056	5.499	10.958	8.115	2.00E-24	6.31E-08
11	effector	CCOR_00143	0.168	104.528	29.478	9.695	7.527	1.62E-127	1.55E-54
12	effector	CCOR_04710	0.450	174.022	72.597	9.130	7.523	1.20E-33	6.87E-11
13	effector	CCOR_06874	0.044	14.439	5.487	8.728	7.100	2.50E-08	7.30E-24
14	effector	CCOR_10304	0.673	133.798	50.610	8.128	6.377	9.62E-38	3.88E-13
15	effector	CCOR_04893	0.223	50.324	14.819	8.281	6.166	1.33E-22	3.58E-17
16	effector	CCOR_02097	2.916	276.750	172.555	7.136	6.112	2.24E-111	1.77E-22
17	effector	CCOR_09360	1.530	332.400	81.134	8.326	5.942	3.31E-22	1.12E-83
18	effector	CCOR_03918	10.968	931.712	469.909	6.987	5.653	4.81E-54	1.21E-20
19	effector	CCOR_00845	0.202	0.754	6.023	--	5.048	--	1.14E-16
20	effector	CCOR_08135	0.111	58.846	3.289	9.501	4.993	9.77E-59	9.77E-07
21	effector	CCOR_08230	0.047	0.165	1.291	--	4.963	--	8.89E-15
22	effector	CCOR_03113	4.249	107.240	111.873	5.221	4.943	5.63E-16	3.79E-52
23	effector	CCOR_10213	4.970	157.623	111.779	5.561	4.718	1.31E-25	2.18E-23
24	effector	CCOR_07800	4.101	148.901	87.285	5.753	4.638	1.16E-53	3.30E-21
25	effector	CCOR_01826	4.391	134.806	89.951	5.513	4.593	6.78E-67	0.0001728
26	effector	CCOR_09255	1.434	15.003	24.341	3.946	4.308	3.34E-12	6.17E-13
27	effector	CCOR_05239	0.332	10.386	5.610	5.486	4.243	2.18E-14	2.82E-15
28	effector	CCOR_01810	20.408	486.444	307.277	5.151	4.143	4.22E-30	2.41E-15
29	effector	CCOR_07853	14.997	403.095	225.958	5.316	4.139	1.28E-71	3.53E-55
30	effector	CCOR_03572	77.265	1340.720	944.419	4.696	3.844	7.69E-22	8.61E-07
31	effector	CCOR_06358	51.540	393.595	458.806	3.501	3.384	9.57E-34	2.40E-19

32	effector	CCOR_08126	1.091	38.408	9.766	5.638	3.325	1.78E-40	0.0001822
33	effector	CCOR_01055	35.412	366.352	295.835	3.954	3.296	7.79E-16	2.48E-05
34	effector	CCOR_05409	14.647	210.314	68.355	5.226	3.254	1.23E-63	1.87E-38
35	effector	CCOR_04734	148.766	1408.424	1172.454	3.807	3.213	1.39E-10	7.33E-21
36	effector	CCOR_09658	2.275	4.779	14.952	1.605	2.936	0.1849725	2.42E-15
37	effector	CCOR_05200	5.122	19.342	31.960	2.485	2.870	5.17E-10	5.97E-26
38	effector	CCOR_08218	1.521	7.750	9.345	2.941	2.840	0.0032293	2.91E-13
39	effector	CCOR_01848	1.351	3.151	7.897	1.779	2.767	0.014804	1.85E-10
40	effector	CCOR_09419	0.265	1.377	1.649	--	2.713	--	4.26E-05
41	effector	CCOR_04333	387.033	2456.957	2158.766	3.253	2.711	9.25E-06	7.94E-06
42	effector	CCOR_09447	144.324	1321.358	776.837	3.778	2.656	1.32E-07	1.51E-07
43	effector	CCOR_03588	19.079	10.487	101.733	-0.298	2.647	0.7448494	5.60E-07
44	effector	CCOR_00140	31.876	54.830	162.414	1.356	2.583	4.88E-05	1.93E-10
45	effector	CCOR_10360	9.881	21.850	47.749	1.713	2.503	0.0005361	1.50E-17
46	effector	CCOR_03575	42.030	70.959	200.025	1.310	2.479	0.0048125	9.17E-24
47	effector	CCOR_06418	13.370	153.416	55.665	4.095	2.288	9.93E-25	1.51E-10
48	effector	CCOR_03447	7.452	20.508	28.855	2.051	2.177	0.0333408	1.87E-09
49	effector	CCOR_05411	13.554	84.032	50.154	3.206	2.120	1.40E-25	1.66E-05
50	effector	CCOR_05000	16.396	43.899	58.004	1.964	2.054	0.003938	2.86E-05
51	effector	CCOR_06295	6.285	4.490	20.317	0.122	1.924	0.9455593	0.0072181
52	effector	CCOR_05165	3.835	9.043	11.636	1.834	1.888	0.1200752	0.0055876
53	effector	CCOR_00531	31.835	557.446	95.136	4.707	1.810	3.03E-40	2.50E-11
54	effector	CCOR_04556	256.105	643.527	741.393	1.886	1.757	1.13E-08	0.0001717
55	effector	CCOR_00058	12.054	6.155	33.798	-0.414	1.714	0.6992957	2.47E-05
56	effector	CCOR_02333	7.398	8.490	20.091	0.767	1.662	0.293333	0.0002105
57	effector	CCOR_10231	117.244	397.574	309.116	2.344	1.629	9.73E-06	3.25E-06
58	effector	CCOR_03675	160.665	705.377	420.320	2.706	1.619	2.79E-10	1.45E-05
59	effector	CCOR_07637	560.643	443.309	1333.431	0.250	1.472	0.4853195	9.29E-07

60	effector	CCOR_04332	18.491	50.507	31.541	2.024	1.005	2.58E-09	0.1921141
61	effector	CCOR_06299	38.909	17.035	51.634	-0.604	0.637	0.1619644	0.0282804
62	effector	CCOR_05781	274.638	515.401	359.287	1.487	0.613	0.0013208	0.0208085
63	effector	CCOR_07550	5.446	32.515	6.409	3.168	0.468	0.0093562	0.2930623
64	effector	CCOR_04113	46.271	84.972	53.814	1.459	0.445	0.0003108	0.0923644
65	effector	CCOR_08163	4.215	0.255	4.400	--	0.293	--	0.8042888
66	effector	CCOR_04980	3.534	35.648	3.393	3.899	0.177	3.40E-10	0.8963026
67	effector	CCOR_03749	3599.454	7089.089	2497.067	1.489	-0.360	0.0030169	0.7063424
68	effector	CCOR_07213	1245.294	1823.139	807.257	1.115	-0.399	0.0005163	0.1961799
69	effector	CCOR_01944	54.430	73.293	31.761	1.007	-0.547	0.0057281	0.075513
70	effector	CCOR_08054	30.079	31.664	17.172	0.641	-0.576	0.080374	0.075876
71	effector	CCOR_00593	101.684	23.441	48.224	-1.554	-0.847	2.88E-06	0.0005429
72	effector	CCOR_00211	11.976	4.502	4.896	-0.819	-1.059	0.1439563	0.0048696
73	effector	CCOR_05950	42.159	91.936	16.400	1.708	-1.130	0.0002844	0.0002825
74	effector	CCOR_08561	9.035	2.354	3.028	-1.330	-1.342	0.0301173	0.0012006
75	effector	CCOR_03801	2.436	0.721	0.810	-1.125	-1.345	0.1206241	0.0053479
76	effector	CCOR_01427	5.681	1.172	1.883	-1.705	-1.370	0.0220542	0.0040889
77	effector	CCOR_04703	8.615	0.665	2.477	-3.021	-1.558	1.01E-05	0.0001584
78	effector	CCOR_09276	34.804	31.492	8.408	0.436	-1.816	0.4113397	2.88E-07
79	effector	CCOR_08158	2.174	0.533	0.487	-1.980	-1.821	0.0135457	0.0003415
80	effector	CCOR_00138	48.455	8.379	8.353	-1.480	-1.827	0.0085398	0.0003594
81	effector	CCOR_10310	4.172	0.634	0.980	-2.109	-1.863	0.0066536	0.000265
82	effector	CCOR_02183	13.876	1.376	3.108	-2.734	-1.929	1.31E-05	0.0002051
83	effector	CCOR_06799	59.263	8.689	9.961	-2.189	-2.343	1.51E-11	6.44E-16
84	effector	CCOR_08009	1899.540	7.272	164.624	-7.460	-3.298	7.63E-65	2.56E-25
85	effector	CCOR_04164	19.087	8.853	1.610	-0.517	-3.316	0.4724717	0.0003704
86	effector	CCOR_07537	2.741	0.060	0.218	-4.694	-3.344	1.28E-06	3.40E-09
87	effector	CCOR_00093	5.965	0.740	0.459	-2.290	-3.404	0.0398952	0.0015009

88	effector	CCOR_07907	22.641	0.572	1.728	-4.650	-3.477	1.57E-16	1.73E-21
89	effector	CCOR_08208	84.368	0.279	5.236	-7.612	-3.806	6.37E-21	2.12E-10

Table S5 The putative CFEM proteins in *Calcarisoprium cordycipiticola* (CC) and *Cordyceps militaris* (CM).

Species	Genes name	Gene ID	CC/CM_fpkm	4 dpi_fpkm	8 dpi_fpkm	4 dpi vs CC/4 dpi vs CM_log2FC	8 dpi vs CC/4 dpi vs CM_log2FC	4 dpi vs CC/4 dpi vs CM_FDR	8 dpi vs CC/8 dpi vs CM_FDR	Pfam_annotation
CC	*Cccfem10*	CCOR_10392	258.01	760.24	516.07	2.13	1.29	0.00	0.00	CFEM domain
CC	*Cccfem11*	CCOR_10414	2.99	38.34	28.74	4.26	4.45	0.00	0.00	CFEM domain
CC	*Cccfem5*	CCOR_05591	3207.54	15829.92	10204.72	2.88	2.19	0.00	0.00	CFEM domain
CC	*Cccfem2*	CCOR_01645	112.21	651.50	592.11	3.11	3.75	0.00	0.00	CFEM domain
CC	*Cccfem7*	CCOR_06625	19.99	34.45	33.47	0.70	1.71	0.05	0.00	CFEM domain
**CC**	***Cccfem8***	**CCOR_08811**	**143.11**	**306.85**	**275.81**	**1.65**	**1.87**	**0.01**	**0.00**	**CFEM domain**
CC	*Cccfem9*	CCOR_09434	9.37	4.97	7.77	-0.33	0.29	0.57	0.61	CFEM domain
CC	*Cccfem4*	CCOR_04017	1011.26	2754.76	1835.55	2.03	0.96	0.00	0.00	CFEM domain
**CC**	***Cccfem6***	**CCOR_06299**	**38.91**	**17.04**	**33.43**	**-0.60**	**0.64**	**0.16**	**0.03**	**CFEM domain**
**CC**	***Cccfem3***	**CCOR_02862**	**156.38**	**267.81**	**205.84**	**1.35**	**0.47**	**0.00**	**0.06**	**CFEM domain**
CC	*Cccfem1*	CCOR_00920	6.08	1.92	4.03	-1.05	-0.34	0.16	0.39	CFEM domain
**CM**	***Cmcfem6***	**CCM_03313**	**1888.14**	**2473.18**	**2254.54**	**0.46**	**0.18**	**0.39**	**0.73**	**CFEM domain**
**CM**	***Cmcfem3***	**CCM_01862**	**448.54**	**440.00**	**409.03**	**0.05**	**-0.84**	**0.94**	**0.05**	**CFEM domain**
**CM**	***Cmcfem14***	**CCM_09541**	**120.97**	**83.98**	**96.51**	**-0.46**	**-0.92**	**0.14**	**0.00**	**CFEM domain**
CM	*Cmcfem5*	CCM_02872	62.86	70.48	62.43	0.24	-0.58	0.58	0.10	CFEM domain
CM	*Cmcfem11*	CCM_08734	28.35	14.17	18.61	-0.93	-1.76	0.06	0.00	CFEM domain
**CM**	***Cmcfem8***	**CCM_05467**	**13.08**	**14.88**	**14.55**	**0.25**	**0.11**	**0.51**	**0.78**	**CFEM domain**
CM	*Cmcfem9*	CCM_05729	12.84	9.56	12.39	-0.36	0.13	0.42	0.80	CFEM domain
CM	*Cmcfem12*	CCM_09020	2.35	4.79	16.37	1.08	4.41	0.06	0.00	CFEM domain
CM	*Cmcfem2*	CCM_00778	9.50	5.01	14.53	-0.86	1.81	0.04	0.00	CFEM domain
CM	*Cmcfem4*	CCM_02295	0.49	0.80	0.70	--	--	--	--	CFEM domain
CM	*Cmcfem7*	CCM_04352	0.68	0.25	0.48	--	--	--	--	CFEM domain
CM	*Cmcfem1*	CCM_00738	0.03	0.04	0.03	--	--	--	--	CFEM domain
CM	*Cmcfem10*	CCM_08030	2.33	2.28	3.78	0.04	1.68	0.98	0.00	CFEM domain
CM	*Cmcfem13*	CCM_09217	0.00	0.48	0.26	--	--	--	--	CFEM domain

Note: Bold represents GPI-anchored CFEM domain protein

Table S6 Genes encoding for HSP in *Cordyceps militaris*.

Gene ID	CM_fpkm	4 dpi_fpkm	8 dpi_fpkm	4 dpi vs CC_log2FC	8 dpi vs CC_log2FC	4 dpi vs CC_FDR	8 dpi vs CC_FDR	NR_annotation
CCM_05892	0.12	149.97	98.29	10.26	10.23	0.00	0.03	heat shock 78
CCM_03788	46.53	291.23	223.11	3.25	2.91	0.00	0.02	Hsp70 nucleotide exchange factor (Fes1)
CCM_07508	111.82	504.37	404.26	2.23	2.49	0.00	0.13	heat shock protein HSP98
CCM_04562	122.07	504.87	358.66	2.11	1.86	0.00	0.01	heat shock 60 (Antigen HIS-62)
CCM_02954	103.78	505.79	313.24	2.07	1.49	0.00	0.05	Hsp70 chaperone Hsp88
CCM_05326	80.55	282.32	223.78	1.86	1.94	0.00	0.00	heat shock 70 kDa protein
CCM_07839	1063.53	3026.23	2224.28	1.55	1.19	0.00	0.17	heat shock protein 90
CCM_04804	1451.31	3692.87	2927.06	1.41	1.30	0.01	0.13	heat shock 70 kDa protein
CCM_07379	70.14	176.38	111.86	1.40	-0.13	0.00	0.83	U-box domain containing protein
CCM_08816	65.65	160.48	111.60	1.35	0.47	0.00	0.18	sensor histidine kinase/response regulator TcsB/Sln1
CCM_08531	7.82	18.10	16.92	1.29	1.74	0.00	0.00	chaperone protein dnaK
CCM_00116	581.48	1304.08	1018.95	1.23	0.93	0.01	0.26	heat shock protein
CCM_04059	89.56	180.61	144.63	1.22	0.98	0.00	0.04	Activator of Hsp90 ATPase
CCM_05087	200.65	447.47	364.09	1.21	1.08	0.00	0.01	glucose-regulated protein
CCM_06217	60.93	125.48	180.48	1.11	2.74	0.00	0.00	HSP20-like chaperone
CCM_01884	14.05	24.29	20.58	0.91	0.74	0.00	0.25	Hsp90 co-chaperone Cdc37
CCM_08543	238.09	390.99	312.08	0.79	0.10	0.06	0.91	Hsp90 binding co-chaperone Sba1
CCM_01217	57.47	85.39	77.13	0.64	0.53	0.03	0.08	heat shock
CCM_09204	6.87	9.94	9.28	0.60	0.60	0.04	0.03	autoinducer 2 sensor kinase/phosphatase luxQ
CCM_08653	81.04	100.54	105.75	0.38	0.73	0.25	0.01	heat shock protein, Hsp40, DnaJ
CCM_03449	30.63	32.01	35.06	0.13	0.41	0.74	0.15	Hsp70 chaperone (BiP), putative
CCM_02931	2.38	2.44	2.73	0.10	0.44	0.90	0.37	DNA mismatch repair protein PMS1
CCM_05489	152.16	148.76	189.59	0.04	0.86	0.99	0.02	heat shock protein 70
CCM_03319	87.50	84.63	94.52	0.02	-0.01	0.99	0.98	Heat shock protein Hsp70

CCM_01005	28.52	25.63	25.53	-0.09	-0.71	0.91	0.08	Hsp40 co-chaperone Jid1, putative
CCM_04288	11.64	10.33	11.15	-0.10	-0.21	0.77	0.56	DNA topoisomerase 2
CCM_00261	9.90	8.81	10.87	-0.10	0.47	0.80	0.11	two-component sensor protein histidine protein kinase
CCM_03553	21.94	18.24	22.87	-0.16	0.37	0.64	0.26	Hsp70 family chaperone, putative
CCM_09141	20.46	17.70	18.27	-0.17	-0.63	0.63	0.03	pyruvate dehydrogenase kinase
CCM_04461	9.10	7.41	8.49	-0.23	-0.19	0.53	0.60	sensor histidine kinase/response regulator
CCM_08162	5.85	4.72	6.18	-0.24	0.45	0.69	0.61	autoinducer 2 sensor kinase/phosphatase luxQ
CCM_01592	5.00	3.90	4.27	-0.28	-0.73	0.58	0.11	TPR domain protein
CCM_01554	46.39	38.39	41.00	-0.32	-0.56	0.45	0.13	two-component osmosensing histidine kinase (Bos1)
CCM_06813	16.27	12.26	14.08	-0.34	-0.49	0.35	0.16	DNA mismatch repair protein
CCM_04285	6.34	4.77	6.05	-0.35	0.04	0.62	0.89	tetratricopeptide repeat domain-containing protein
CCM_07878	33.50	23.22	27.55	-0.45	-0.64	0.13	0.02	sensor histidine kinase/response regulator TcsB/Sln1
CCM_00658	83.52	57.39	70.81	-0.48	-0.43	0.24	0.28	Hsp70 family chaperone
CCM_07380	5.84	3.61	4.83	-0.51	-0.43	0.21	0.37	Hsp90 co-chaperone Cdc37
CCM_04086	16.71	11.01	14.80	-0.54	-0.12	0.14	0.83	DNA mismatch repair protein Mlh1
CCM_02360	41.18	26.55	33.65	-0.57	-0.54	0.11	0.12	Hsp70 family chaperone
CCM_06910	12.08	7.88	13.19	-0.57	0.80	0.15	0.01	Hsp70 family chaperone
CCM_09248	41.01	23.94	29.13	-0.70	-1.42	0.02	0.00	ThiJ/PfpI family protein
CCM_01061	23.21	13.09	18.86	-0.76	-0.35	0.02	0.32	pyruvate dehydrogenase kinase
CCM_07879	3.81	2.11	3.60	-0.78	0.38	0.12	0.42	ethylene receptor
CCM_00801	9.72	3.40	5.89	-1.44	-1.63	0.02	0.01	Hsp40 co-chaperone Jid1
CCM_05412	14.14	3.98	8.98	-1.76	-0.92	0.00	0.00	ethylene receptor
CCM_01403	0.08	0.04	0.07	--	--	--	--	Hsp70 family protein
CCM_02964	0.00	0.00	0.00	--	--	--	--	kinase isozyme 4
CCM_03318	0.01	0.04	0.02	--	--	--	--	Heat shock Hsp70
CCM_06821	0.00	0.00	0.00	--	--	--	--	30 kDa heat shock protein
CCM_07174	0.10	0.02	0.05	--	--	--	--	chaperonin

Table S7 Primers used in this study.

Primer Name	Primer Sequence (5' to 3')	Description
qCctef_CCOR_04235-F	CTACCAGTGCGGAGGTATCG	Reference gene of *C. cordycipiticola*
qCctef_CCOR_04235-R	TGTCAAGGACCCAGGCGTA	
qCcCBM1_CCOR_06535-F	CGATCTCGTCGGAGGTGAC	*Cccbm18* fluorescence quantitative detection
qCcCBM1_CCOR_06535-R	CAGCGGTGCAGATGTCGTT	
qCcCBM1_CCOR_09350-F	CCCTCGCTATGCTCCTCTCTA	
qCcCBM1_CCOR_09350-R	GGGGAAGTAGGTGCTGATGC	
qNPS6_CCOR_00725-F	CAGGCTGGAACGCTTTATTTTA	NPS6 Cluster fluorescence quantitative detection
qNPS6_CCOR_00725-R	CCGAGTAGGGGAAGTAGAGGAG	
qNPS6_CCOR_00726-F	GGCTGTCGATGAAGAGGGAT	
qNPS6_CCOR_00726-R	AGGACGCTCTGGTGGATGTT	
qNPS6_CCOR_00727-F	CGAGTCTGGCTCAGATGAATCT	
qNPS6_CCOR_00727-R	GTGCTTGTTAAAGGCTGAGGTG	
qNPS6_CCOR_00728-F	TGCGTACTTTGAGGTGTACTGGG	
qNPS6_CCOR_00728-R	TTGAGTTGGTGTCCTTGGGTTC	
qNPS6_CCOR_00729-F	CATCCACGTTATCGGCTATCTC	
qNPS6_CCOR_00729-R	CTGCTCGTAAACTCGGTCAT	
qNPS6_CCOR_00730-F	CGACGATAATGATTCTGGGTC	
qNPS6_CCOR_00730-R	GATGGTTCTGCGAGTATTGAGT	
qCmrpb1_CCM_05485-F	CTGTTCCCCCTCCTCCTGTG	Reference gene of *C. militaris*
qCmrpb1_CCM_05485-R	ATGTTGCGGCGATCCTTCTC	
qCmhyd1_CCM_03537-F	CCTCTACTCCAACCCCATCTG	*Cmhyd1* fluorescence quantitative detection
qCmhyd1_CCM_03537-R	CAGAGGACTGCCTGGTCAAT	
qCmhsp78_CCM_05892-F	CCCGAAAGCATAAAGAACA	*Cmhsp78* fluorescence quantitative detection
qCmhsp78_CCM_05892-R	TTGGAGGCGTCAATGGAAC	

qCCM_03788_hsp70_F	TTCGCAACTACCAGCCCGC	*Cmhsp70* fluorescence
qCCM_03788_hsp70_R	TGTCCACCGCCTCCATGTC	quantitative detection

Table S8 GPCRs genes in *Calcarisporium cordycipiticola*.

GPCR classes	Description GPCR Class	Gene ID	CC_fpkm	4 dpi_fpkm	8 dpi_fpkm	4 dpi vs CC_log2FC	8 dpi vs CC_log2FC	4 dpi vs CC_FDR	8 dpi vs CC_FDR
I	Pheromone	CCOR_04397	11.50753	3.229426	7.7850284	-1.2637698	-0.1010185	0.0141149	0.8087624
II	Pheromone	CCOR_05130	1.122667	0.5877153	0.9583937	-0.3084582	0.4393032	0.6620241	0.4058386
II	Pheromone	CCOR_01411	16.579717	4.8559363	9.6743893	-1.2010067	-1.2135612	1.73E-05	2.31E-07
III	Rhodopsin/Carbon Sensory	CCOR_09332	17.973287	11.261393	15.974069	-0.1114176	0.4168829	0.8608358	0.1264374
IV	PQ loop	CCOR_02432	34.181083	27.575279	29.486709	0.2563263	-0.2309803	0.4402899	0.408531
IV	PQ loop	CCOR_03488	14.2049	7.2037143	10.665964	-0.4175087	-0.2011321	0.4615644	0.5518303
V	Secretin	CCOR_05467	9.5512693	2.3880917	6.6560618	-1.4003941	0.1299521	0.0050186	0.7160504
VI	Complex sensor containing a RGS domain	CCOR_09200	388.07705	320.07014	437.14369	0.260517	1.0802315	0.5810972	0.0040417
VI	Complex sensor containing a RGS domain	CCOR_04339	32.227146	16.460959	28.43818	-0.4193012	0.6066778	0.5922212	0.4609484
VII	MG00532-like	CCOR_02803	24.762105	5.0682817	17.857763	-1.7096134	0.3865351	6.59E-07	0.1338256
VII	MG00532-like	CCOR_01839	10.337765	19.006027	16.938712	1.2227763	1.4437274	0.0004939	5.74E-09
VIII	Haemolysin	CCOR_03378	10.887578	1.9609977	5.7465295	-1.8840726	-1.4134852	0.0007471	0.000101
VIII	Haemolysin	CCOR_02996	41.195578	12.068803	25.214309	-1.2030441	-0.781276	3.06E-05	0.0012565
IX	Microbial opsins	CCOR_05453	111.43667	425.72237	246.23658	2.5098839	0.8516211	1.05E-15	0.0005062
X	Lung 7TM Superfamily or PTM1-like GPCR	CCOR_02599	37.79609	34.034051	35.932785	0.4211169	0.157823	0.1771879	0.5761958
XI	GPCR89/ABA-GPCR	0							
XII	Family C-like	CCOR_05961	121.10357	8.4782873	59.997294	-3.3508612	-1.2333857	3.39E-37	7.81E-07
XIII	DUF300 superfamily/PsGPR11	CCOR_01323	0.735934	4.6738833	2.5386304	3.2180999	1.6400175	8.44E-07	0.0005618
XIII	DUF300 superfamily/PsGPR11	CCOR_00911	20.888709	14.643231	18.711342	0.0567868	0.2957883	0.875055	0.2953009
XIII	DUF300 superfamily/PsGPR11	CCOR_09173	10.022765	5.479337	9.6979413	-0.4265988	0.9227052	0.3878643	0.000265
XIV	PTH11	CCOR_05703	110.83444	62.438178	88.371341	-0.1422788	-0.0400726	0.6758512	0.8765237
XIV	PTH11	CCOR_04605	4.0728187	0.3567313	2.0420028	-2.8080261	-1.2440812	0.0000398	0.0053514
XIV	PTH11	CCOR_06769	2.5129723	28.202445	15.901355	4.0651399	3.0421502	0.0000754	4.08E-22
XIV	PTH11	CCOR_05682	31.905163	2.360636	15.862211	-3.1460183	-1.2255753	3.4E-17	1.54E-06

Table S9 Lectin-related genes in *Calcarisporium cordycipiticola* (CC) and *Cordyceps militaris* (CM).

Species	Gene ID	CC/CM_fpkm	4 dpi_fpkm	8 dpi_fpkm	4 dpi vs CC/4 dpi vs CM_log2FC	8 dpi vs CC/4 dpi vs CM_log2FC	4 dpi vs CC/4 dpi vs CM_FDR	8 dpi vs CC/8 dpi vs CM_FDR
CC	CCOR_01894	8.4972237	142.73374	84.788084	4.6349237	3.991655	4.58E-46	5.51E-58
CC	CCOR_03676	0.6084453	146.74659	76.126196	8.4625509	7.3325294	2.44E-143	1.68E-28
CC	CCOR_08831	6.612823	34.147497	27.939946	2.9324994	3.2342657	3.40E-24	3.37E-40
CC	CCOR_08882	29.087094	135.02358	80.085565	2.7814564	1.5663293	2.84E-22	1.87E-11
CC	CCOR_01446	95.770853	197.82397	141.33481	1.6100239	0.5864326	1.17E-07	0.0229777
CC	CCOR_08418	62.637385	87.509562	72.018612	1.0535684	0.2086841	0.0002882	0.4344472
CC	CCOR_01100	136.75791	62.665382	95.754539	-0.55892	-0.501066	0.0534806	0.0466473
CC	CCOR_01584	7.1061153	5.9904633	6.4585307	0.3226148	0.0231405	0.5977463	0.9913975
CC	CCOR_06488	10.078534	10.451377	10.027697	0.6151562	0.1037727	0.2026591	0.8557817
CC	CCOR_06501	11.763589	4.583437	8.3725334	-0.795781	-0.154964	0.0063069	0.6165438
CC	CCOR_08641	43.95299	18.950197	34.377445	-0.649184	0.2337451	0.0321539	0.3812302
CC	CCOR_07013	12.765231	3.7532857	7.8038966	-1.285503	-0.840386	0.0006229	0.0020436
CC	CCOR_09537	55.867354	16.952923	33.727404	-1.156329	-0.9443	4.24E-05	7.19E-05
CC	CCOR_04845	0.0178043	0.1840633	0.118803	--	--	--	--
CM	CCM_05447	20.242294	40.134046	31.934046	1.0612589	0.687636	5.58E-05	0.0125484
CM	CCM_04992	1.981599	1.5102913	2.2871086	-0.324732	0.821459	0.6027147	0.0529281
CM	CCM_07787	42.578451	54.08043	61.920147	0.4363458	1.1404388	0.6574467	0.0076615
CM	CCM_01589	98.339737	153.20762	127.95951	0.7114841	0.2418446	0.0244807	0.4505497
CM	CCM_01779	85.874078	124.75908	109.49876	0.6112095	0.3128173	0.0842756	0.4122463
CM	CCM_01250	11.401083	11.970943	12.655793	0.1424653	0.2586337	0.7989455	0.506542
CM	CCM_01136	16.133191	9.3267603	14.613224	-0.723869	0.1550118	0.0099468	0.6739575
CM	CCM_03227	9.9715977	7.706394	9.5293386	-0.304186	0.0303926	0.7022986	0.9758293
CM	CCM_09483	6.0304003	5.3282387	5.9642577	-0.110269	-0.020563	0.8360886	0.9642181

CM	CCM_08208	7.5978387	4.7697317	6.0841574	-0.604846	-0.620137	0.1970148	0.1852844
CM	CCM_06271	1.7056127	1.7261613	1.6988925	0.0807886	-0.264762	0.9370704	0.7133695
CM	CCM_03679	3.6505937	1.7260053	2.4786201	-1.021088	-1.254429	0.1160434	0.0383493
CM	CCM_03832	5214.7682	3185.7759	3719.8964	-0.6432	-1.521067	0.2265436	0.0020328
CM	CCM_06464	175.60435	246.57383	187.56019	0.5584474	-0.90939	0.1125896	0.0016681
CM	CCM_03178	40.84072	14.825626	37.436867	-1.38837	0.5554495	0.0003301	0.0667224
CM	CCM_05769	0.518962	0.2497803	0.3598403	--	--	--	--
CM	CCM_04539	0.2415137	0.1395057	0.1904543	--	--	--	--
CM	CCM_02177	0	0.0161093	0.012641	--	--	--	--

Table S10 DEG CAZyme genes in *Calcarisporium cordycipiticola* (CC) and *Cordyceps militaris* (CM).

CAZyme classification	Gene ID	CC/CM_fpkm	4 dpi_fpkm	8 dpi_fpkm	4 dpi vs CC/4 dpi vs CM_log2FC	8 dpi vs CC/4 dpi vs CM_log2FC	4 dpi vs CC/4 dpi vs CM_FDR	8 dpi vs CC/8 dpi vs CM_FDR
AA1_3	CCOR_10256	16.394459	132.38849	143.43665	3.5799373	3.3559046	2.44E-35	1.10E-42
AA11	CCOR_03113	4.2491977	107.24035	111.87341	5.2207431	4.9432515	5.63E-16	3.79E-52
AA11	CCOR_01848	1.35114	3.1510187	7.8968487	1.7793188	2.7667529	0.014804	1.85E-10
AA12	CCOR_05890	78.887955	286.63608	441.53417	2.4286117	2.7153603	8.11E-16	2.26E-15
AA2	CCOR_01769	4.6001363	183.23077	53.947169	5.8791992	3.7789331	3.77E-82	8.18E-39
AA2	CCOR_05190	1.8880567	4.9329427	3.582395	1.4566778	1.0179559	0.0021161	0.0019752
AA2	CCOR_03182	32.908562	63.594838	11.902396	1.4477955	-1.089528	0.0034195	0.3157874
AA2	CCOR_09102	1.118352	0.6288223	2.680359	-0.234348	1.4879684	0.8574822	0.0006696
AA3	CCOR_01717	0.4146323	10.133268	3.7202947	5.13754	3.3674271	1.09E-27	1.15E-07
AA3	CCOR_05011	36.839614	140.91595	133.0431	2.5073856	2.0792254	7.32E-18	1.93E-18
AA3	CCOR_01074	30.716639	11.195308	30.337757	-0.88601	0.2082547	0.0022346	0.4457333
AA3	CCOR_00497	613.82113	102.90475	287.25688	-2.004433	-0.86779	2.87E-12	0.0056681
AA3	CCOR_04454	5.7949663	0.707994	1.0815287	-2.434634	-2.186189	1.30E-05	3.02E-09
AA3	CCOR_07910	74.41906	2.0988937	1.9285967	-4.570912	-5.044559	2.36E-15	3.16E-19
AA3_2	CCOR_09575	5.5218473	13.290904	12.683707	1.8388429	1.4274679	8.42E-07	2.88E-07
AA3_2	CCOR_09169	40.128398	66.305737	97.71435	1.2951625	1.5108664	6.76E-06	1.37E-10
AA3_2	CCOR_00560	6.526887	5.7290187	19.755252	0.3809883	1.8257568	0.4664331	8.73E-13
AA3_2	CCOR_05581	17.527289	12.37459	32.763037	0.0709844	1.1299429	0.9072331	2.83E-06
AA3_2	CCOR_05702	54.265388	18.301421	21.877255	-1.016278	-1.091147	0.0003061	2.90E-06
AA3_2	CCOR_09652	54.794032	16.254074	48.608708	-1.186121	0.0529805	1.65E-05	0.8377731
AA3_2	CCOR_06191	2.1016917	0.3736333	2.024495	-1.926077	0.1720365	0.0105116	0.7886881
AA3_2	CCOR_00591	0.8146293	0.0940643	0.168386	-2.364691	-2.018283	0.0029945	9.73E-05
AA3_3	CCOR_01998	10.742902	6.134692	42.129864	-0.25234	2.1956491	0.7053871	1.06E-10
AA3_3	CCOR_02394	36.808732	10.584478	23.213	-1.376927	-0.443125	8.58E-07	0.0970408

AA4	CCOR_04160	88.581685	263.3539	158.27087	2.1424416	1.0644694	1.10E-09	0.0004026
AA7	CCOR_01697	28.393135	69.148109	77.272016	1.8424322	1.6736961	6.70E-10	2.53E-13
AA7	CCOR_07968	1051.4179	943.3422	1932.6049	0.401976	1.1078337	0.3326802	0.0032459
AA7	CCOR_08189	20.046518	13.454357	80.465979	0.2457754	2.1564178	0.5080307	1.28E-10
AA7	CCOR_08193	38.559904	26.931423	114.31817	0.0513766	1.7950352	1	1.01E-11
AA7	CCOR_07966	48.18144	15.550252	60.571271	-1.058935	0.5574255	0.0001881	0.0191318
AA7	CCOR_01799	852.23984	219.80714	808.48169	-1.397956	0.1553914	4.62E-05	0.698303
AA7	CCOR_01008	6.2320153	1.1171	4.6819887	-1.907939	-0.183904	0.0011381	0.6811629
AA7	CCOR_02835	2.054203	0.3345783	0.8249963	-2.029176	-1.086309	0.0062273	0.0221375
AA7	CCOR_05681	14.053212	1.914435	5.6513897	-2.287074	-1.083763	9.83E-08	0.0001712
AA7	CCOR_03564	6.69204	0.5556217	3.167413	-2.428849	-0.824641	8.17E-06	0.017645
AA7	CCOR_00025	18.630254	2.0883797	23.925213	-2.633364	0.5808077	2.43E-10	0.0221864
AA7	CCOR_09378	2390.5578	206.0078	1646.5754	-2.971149	-0.316065	4.36E-18	0.5285103
AA7	CCOR_03070	6.9336677	0.5436147	8.452981	-3.070313	0.5147392	5.05E-07	0.1384316
CBM13	CCOR_08831	6.612823	34.147497	53.139233	2.9324994	3.2342657	3.40E-24	3.37E-40
CBM18	CCOR_09360	1.530408	332.39967	81.133591	8.3264495	5.9417793	3.31E-22	1.12E-83
CBM18	CCOR_06535	8.873688	1559.9777	428.50387	8.0373341	5.8200519	6.12E-20	2.99E-98
CBM18	CCOR_01694	1.458979	0.0174867	0.348235	-5.338109	-2.17801	1.98E-08	1.13E-05
CBM20	CCOR_08891	20.685265	6.1031093	10.783108	-1.178138	-0.715961	0.0002274	0.007779
CBM21	CCOR_01735	182.18726	18.72937	37.814577	-2.724236	-2.049391	2.29E-23	3.09E-14
CBM24	CCOR_04359	25.56148	85.762919	195.91001	2.3307862	3.1653956	0.0006741	4.26E-14
CBM50	CCOR_04819	78.192929	23.075841	36.601573	-1.19179	-0.866638	0.0002276	0.0004869
CE1	CCOR_03121	40.639747	174.32001	188.79089	2.6674338	2.4397551	2.91E-15	2.52E-21
CE1	CCOR_03929	7.3757087	24.028842	34.101787	2.2847304	2.4390408	0.0006045	5.56E-08
CE1	CCOR_05000	16.396237	43.899064	58.003752	1.963983	2.0543115	0.003938	2.86E-05
CE10	CCOR_00053	7.1559743	71.017069	45.797473	3.886393	2.9073206	1.56E-23	9.63E-17
CE10	CCOR_09398	6.978843	10.674885	11.153237	1.1963704	0.9070076	0.0032659	0.0019622
CE10	CCOR_07053	20.903124	49.060198	32.344099	1.1306029	0.1877498	0.0001554	0.505575

CE10	CCOR_00245	20.544638	9.981668	25.227657	-0.478036	0.526412	0.1750205	0.0449017
CE10	CCOR_08216	15.030922	4.4709453	5.6412353	-1.177325	-1.188031	0.0232513	0.0006417
CE10	CCOR_05460	5.8469503	1.1866097	1.919398	-1.570546	-1.29575	0.0040965	0.0003915
CE10	CCOR_04564	9.6053093	2.0485323	9.9924753	-1.692775	0.2901888	0.0048422	0.5445643
CE10	CCOR_07704	13.411737	1.0323713	0.185055	-3.148816	-5.907567	1.62E-10	2.64E-47
CE10	CCOR_03972	2.9946243	0.1369677	2.431334	-3.739622	-0.073735	2.24E-06	0.9146977
CE10	CCOR_08655	22.370454	0.805559	2.18002	-4.194394	-3.127136	2.67E-05	0.0001855
CE16	CCOR_05998	36.179895	48.700714	64.944829	0.9992776	1.0750261	0.0009596	3.79E-05
CE16	CCOR_00645	3.376099	4.214814	28.074866	0.8909641	3.282468	0.1938227	1.84E-27
CE3	CCOR_08103	8.644672	316.56804	141.51997	5.7688883	4.2573726	6.40E-16	9.68E-25
CE3	CCOR_08148	2.5424383	10.203369	14.56094	2.4413444	2.8702416	3.96E-06	3.85E-07
CE3	CCOR_10230	12.573462	30.158796	64.210676	1.8180224	2.5826761	4.25E-08	6.87E-13
CE3	CCOR_06713	13.293217	13.852995	70.895729	0.6374133	2.638979	0.1989881	6.78E-27
CE4	CCOR_09327	2.302621	0.5610247	0.726834	-1.38238	-1.420094	0.0507728	0.0023034
CE5	CCOR_06055	1.4924503	12.937392	18.070977	3.6561996	3.8243789	6.82E-05	3.35E-07
CE5	CCOR_03354	73.549431	169.16895	148.04334	1.7718082	1.2358461	6.42E-10	8.49E-08
CE5	CCOR_06530	10.929239	11.916063	6.3255627	0.7051494	-0.558038	0.4222061	0.5768986
CE9	CCOR_04531	17.29699	36.452987	80.470502	1.6255539	2.4453282	0.0004436	2.57E-26
GH1	CCOR_08653	15.606626	21.875515	23.387097	1.0452047	0.8104516	0.0004724	0.0008326
GH1	CCOR_01406	42.665923	42.908698	78.701579	0.5719798	1.108369	0.0939533	5.16E-07
GH10	CCOR_08654	1.156007	0.159831	0.39055	-2.078859	-1.318979	0.0062619	0.0163206
GH105	CCOR_05189	0.0856137	3.292194	5.8002737	--	6.2105595	--	1.29E-32
GH105	CCOR_01846	12.415996	32.788998	82.0777	1.9497326	2.9549894	0.0013258	2.88E-35
GH109	CCOR_05504	49.429493	69.650935	42.804473	1.0728007	0.0214656	0.0004392	0.9738688
GH12	CCOR_05495	56.247026	113.72899	187.64505	1.5865733	1.9734122	0.0125058	0.0017541
GH125	CCOR_08063	35.014563	49.117209	118.51705	0.9713393	1.9841343	0.0008223	2.75E-17
GH125	CCOR_04513	25.498304	26.220088	63.391905	0.6012067	1.5405663	0.0421155	1.22E-11
GH128	CCOR_08240	0.051575	2.2589723	8.9617637	--	7.5931629	--	1.39E-41

GH128	CCOR_01213	475.28637	1745.7783	1361.8182	2.4529796	1.7461164	1.28E-11	9.60E-09
GH13_1	CCOR_07632	1.426182	8.1675903	15.908383	3.0717569	3.70041	8.07E-09	4.46E-30
GH13_22	CCOR_08102	11.233756	0.034031	0.2169867	-7.732164	-5.466995	2.40E-10	1.06E-07
GH13_32	CCOR_04977	2.1564253	5.1832817	14.25281	1.8256704	2.9492105	0.000197	1.36E-26
GH13_40	CCOR_06830	0.0780137	14.886403	10.003387	8.015113	7.1139869	3.49E-64	1.54E-61
GH130	CCOR_00647	0.9263347	4.3168473	12.333077	2.7592887	3.9507337	0.0033738	2.71E-11
GH16	CCOR_09367	0.655045	59.250624	34.651961	7.0588723	5.9375849	1.36E-52	1.45E-27
GH16	CCOR_10348	16.449369	453.17464	385.16256	5.3516154	4.7817229	2.77E-30	8.39E-36
GH16	CCOR_05118	5.1350167	101.60473	79.274139	4.8675653	4.1719302	1.40E-58	1.25E-59
GH16	CCOR_05314	3.050243	12.08748	10.248313	2.5572084	1.9744733	4.03E-07	4.79E-08
GH16	CCOR_00820	468.75013	1521.4344	993.01731	1.8040464	1.3113051	6.92E-06	0.0001793
GH16	CCOR_01121	18.709097	36.301933	39.281712	1.5262743	1.2994305	1.15E-06	8.93E-08
GH16	CCOR_05781	274.63807	515.4014	359.28651	1.4873528	0.6125076	0.0013208	0.0208085
GH16	CCOR_05134	243.52393	113.90225	56.44237	-0.594526	-1.066947	0.0358604	4.09E-05
GH16	CCOR_08527	50.39709	20.643179	16.206456	-0.743313	-1.418089	0.0060368	4.73E-07
GH17	CCOR_06659	2.5528007	0.1369667	3.0085157	--	0.483391	--	0.8259415
GH18	CCOR_07055	4.500121	60.351865	73.78478	4.3067126	4.2630094	2.71E-40	1.08E-63
GH18	CCOR_10110	8.4055157	104.21384	116.48789	4.1975432	4.024928	6.86E-46	9.26E-14
GH18	CCOR_08352	11.822867	136.77012	282.86679	4.0930987	4.8112263	6.71E-20	7.05E-23
GH18	CCOR_00575	14.914113	101.90171	43.313981	3.3484116	1.7605771	1.84E-19	5.52E-05
GH18	CCOR_09203	12.51502	25.207964	27.708866	1.5731893	1.3794651	6.69E-06	0.0152683
GH18	CCOR_00381	15.906017	11.8241	36.698169	0.1795304	1.2089397	0.5906329	2.27E-07
GH18	CCOR_04883	33.285708	10.601738	12.325313	-1.085318	-1.204986	0.0014825	2.06E-06
GH18	CCOR_03036	104.81141	0.50266	3.4961407	-7.082303	-4.677569	6.69E-79	5.73E-56
GH2	CCOR_03578	21.831219	77.209287	242.13566	2.4308685	3.5879081	9.53E-05	1.38E-18
GH2	CCOR_05129	2.347153	7.923294	21.646787	2.3097183	3.4334433	4.50E-06	1.01E-43
GH2	CCOR_04821	49.169075	160.12994	333.06851	2.2609693	2.9900506	2.81E-11	3.61E-27
GH2	CCOR_01898	14.520291	36.571925	48.195896	1.8988068	1.9558796	3.95E-11	1.36E-16

GH2	CCOR_02213	11.354715	12.413607	48.501983	0.6762606	2.3224872	0.2123011	2.01E-23
GH2	CCOR_08194	16.21787	13.648114	66.257685	0.2936146	2.258754	0.5434462	1.21E-22
GH2	CCOR_01468	20.629796	7.4490447	7.8289347	-0.77753	-1.167482	0.0118594	1.47E-06
GH2	CCOR_04831	26.897311	7.225402	13.273922	-1.33288	-0.794571	2.71E-05	0.0040465
GH20	CCOR_03570	44.744206	55.291322	187.84583	0.8562052	2.2983276	0.0236021	8.40E-20
GH20	CCOR_03764	27.219286	32.665226	46.573512	0.8271871	1.0020336	0.004973	2.26E-05
GH23	CCOR_04381	1.607876	4.7484437	7.0184437	2.1349492	2.3563805	0.0020201	0.0003678
GH25	CCOR_04332	18.490642	50.506798	31.540558	2.0239978	1.0052035	2.58E-09	0.1921141
GH27	CCOR_01894	8.4972237	142.73374	115.36343	4.6349237	3.991655	4.58E-46	5.51E-58
GH28	CCOR_03575	42.029834	70.95888	200.02515	1.3102477	2.4789229	0.0048125	9.17E-24
GH28	CCOR_01751	3.8513133	2.4904117	14.181665	-0.07947	2.1053305	1	1.38E-11
GH29	CCOR_03480	3.1230093	0.6041913	1.836968	-1.821148	-0.535089	0.0087132	0.2257942
GH3	CCOR_05158	15.614844	107.22294	71.140104	3.3519607	2.4124777	2.87E-27	1.26E-25
GH3	CCOR_06961	31.357139	51.981238	91.783969	1.3060192	1.7277117	4.81E-06	2.22E-12
GH3	CCOR_01564	76.354005	110.50364	210.52241	1.0910414	1.6858646	0.0001814	3.13E-10
GH3	CCOR_00994	65.900394	66.203844	125.11086	0.5715529	1.1493476	0.0440872	7.22E-06
GH3	CCOR_06464	10.18827	8.576204	18.030988	0.3102116	1.0529251	0.367314	1.91E-05
GH3	CCOR_08555	21.994452	8.623573	7.3568163	-0.770181	-1.347724	0.0467232	1.65E-06
GH31	CCOR_08245	13.521124	26.711373	47.369259	1.0681916	1.5852419	0.012669	6.06E-07
GH31	CCOR_06967	11.413545	18.776293	22.204595	1.0508617	1.0968933	0.0002717	2.27E-05
GH31	CCOR_04375	68.720811	27.448345	74.905289	-0.760245	0.3490388	0.0076498	0.2662976
GH31	CCOR_01845	30.441934	5.2386603	14.24978	-1.972854	-0.868336	3.57E-07	0.0027635
GH31	CCOR_03846	1.381071	0.129522	0.6959213	-2.72372	-0.759411	0.0001533	0.1003505
GH35	CCOR_01893	4.3111303	61.754734	57.167464	4.4060581	3.9586979	1.58E-51	4.13E-20
GH35	CCOR_07832	7.847246	11.913621	7.1967527	1.178793	0.1013091	0.0002829	0.7257707
GH35	CCOR_04035	14.027029	21.393306	28.388685	1.1650442	1.2403665	3.19E-05	1.92E-06
GH37	CCOR_09101	26.351058	29.288053	81.656319	0.710417	1.8593202	0.0128972	1.18E-15
GH38	CCOR_01164	40.021397	111.14756	207.60364	1.898967	2.4847361	1.01E-06	1.01E-18

GH43	CCOR_03966	30.033344	73.813655	135.97823	1.8592275	2.4073926	1.06E-10	1.51E-23
GH43_26	CCOR_05191	0.102552	0.7458643	1.3948237	--	3.7778406	--	1.12E-11
GH43_26	CCOR_02114	1.403845	9.222852	41.57364	3.291972	5.1118138	0.0009503	6.96E-27
GH43_30	CCOR_09338	8.2896303	3.000882	2.362098	-0.894713	-1.577112	0.1500499	4.61E-05
GH43_33	CCOR_03561	32.437379	9.930334	18.017563	-1.145157	-0.621757	0.0001651	0.0159686
GH43_6	CCOR_07117	3.4064163	2.3460977	3.638657	-0.622297	0.3617546	0.1406534	0.2203021
GH47	CCOR_02884	0.294888	19.471628	0.36379	6.5447259	--	0.0032884	--
GH47	CCOR_09423	40.939731	247.68047	296.57667	3.1572027	3.0850665	6.60E-26	7.93E-31
GH47	CCOR_07937	24.290783	60.451853	87.860067	1.8689164	2.0832136	7.75E-07	1.84E-19
GH5_11	CCOR_02546	40.192431	134.28108	218.17431	2.3045525	2.6688918	8.11E-16	6.34E-27
GH5_15	CCOR_05326	7.225169	38.928245	29.994223	3.0099614	2.2826704	2.47E-06	5.23E-19
GH5_15	CCOR_01875	2.780872	1.85723	6.3472693	-0.010654	1.4186478	1	0.0092963
GH5_31	CCOR_04996	4.921505	0.4374097	1.2177817	-2.85207	-1.842358	2.75E-06	2.77E-06
GH5_5	CCOR_09595	6.1035027	1.462191	9.17772	-1.464685	0.8143436	0.0257418	0.0319783
GH5_9	CCOR_04544	9.1779	17.051438	17.511532	1.4597398	1.1589261	6.99E-06	4.58E-06
GH51	CCOR_04932	2.4866923	6.444405	9.7871307	1.9740176	2.1775289	0.0440872	6.89E-12
GH54	CCOR_02115	19.680944	84.300583	77.735769	2.6691085	2.2105358	8.84E-21	1.03E-06
GH54	CCOR_09537	55.867354	16.952923	24.784956	-1.156329	-0.9443	4.24E-05	7.19E-05
GH54	CCOR_04899	5.6745273	1.2208683	2.4136433	-1.631165	-1.005997	0.0052089	0.0115732
GH55	CCOR_01570	45.082939	36.366569	88.112757	0.2421577	1.1946463	0.4596331	1.03E-06
GH55	CCOR_03930	2.9940573	0.9075913	3.998461	-1.112519	0.6431649	0.0698143	0.0818841
GH6	CCOR_03460	15.251779	5.1892657	4.8604583	-1.258342	-1.479788	0.0002712	2.72E-08
GH64	CCOR_03586	0.27	10.779526	48.877311	5.8593403	7.6967195	9.10E-19	2.38E-51
GH64	CCOR_03438	5.6515423	29.092629	16.946746	2.9312425	1.8149777	4.61E-19	1.92E-05
GH64	CCOR_04347	3.7061957	10.303883	9.3236207	2.0432207	1.5595515	1.39E-05	0.0004675
GH64	CCOR_02602	28.406644	17.427234	44.937951	-0.13524	0.8887275	0.7427004	0.0002095
GH67	CCOR_00054	1.8580253	0	5.581792	--	1.8080428	6.98E-14	7.75E-08
GH71	CCOR_05364	6.1672873	21.656485	16.452552	2.305855	1.6489845	9.02E-05	6.09E-12

GH71	CCOR_03668	4.4703137	0.9634817	1.2414213	-1.633673	-1.610688	0.0136746	0.000137
GH72	CCOR_05536	510.80035	985.82369	655.9164	1.524719	0.5876889	5.12E-05	0.0829968
GH75	CCOR_10191	8.9574667	49.980821	27.177733	3.0631189	1.80797	2.07E-06	9.24E-15
GH75	CCOR_04380	2.627229	5.0063987	6.4590943	1.5179073	1.521372	0.0665829	0.0005062
GH75	CCOR_07907	22.641251	0.5723663	1.7280963	-4.650417	-3.476811	1.57E-16	1.73E-21
GH76	CCOR_03676	0.6084453	146.74659	84.288453	8.4625509	7.3325294	2.44E-143	1.68E-28
GH76	CCOR_05199	1.9254043	86.304349	55.280313	5.3133886	4.4696198	5.04E-70	8.62E-09
GH76	CCOR_02172	6.1447377	131.22699	119.66159	4.9774662	4.5122405	1.15E-63	1.14E-48
GH76	CCOR_08219	6.1369023	28.519346	22.012971	2.7746509	2.0670212	7.85E-18	1.16E-15
GH76	CCOR_05996	37.41014	77.703832	84.278585	1.6180875	1.4001169	1.56E-08	1.22E-09
GH76	CCOR_07296	27.375607	51.950082	35.086243	1.3909164	0.5885253	2.35E-06	0.0175206
GH76	CCOR_04337	8.9711873	6.1547713	22.532788	0.0125913	1.5557494	0.8826649	8.55E-10
GH76	CCOR_09566	9.7727723	5.103533	15.527802	-0.372762	0.8950025	0.4595344	0.0007679
GH76	CCOR_05742	26.528727	12.374911	36.701877	-0.528393	0.6987392	0.13842	0.0066156
GH78	CCOR_10154	3.7413543	0.4840007	0.5187163	-2.371791	-2.612927	0.0002538	7.74E-06
GH81	CCOR_09506	3.124911	5.0533593	32.349445	0.7550702	3.2403311	0.080374	1.28E-42
GH84	CCOR_00579	14.693719	15.484468	42.913846	0.622911	1.7736522	0.1185953	8.47E-15
GH88	CCOR_04871	56.827077	16.348566	36.439775	-1.238929	-0.413225	1.71E-05	0.1000498
GH89	CCOR_01482	72.227223	11.610804	22.472823	-2.078348	-1.455748	8.63E-14	3.55E-10
GH92	CCOR_08159	9.7143393	25.976835	24.462816	1.9845894	1.5594221	3.17E-12	6.51E-12
GH92	CCOR_00585	12.914121	29.974256	60.363405	1.7629202	2.4550858	0.0018749	8.51E-12
GH92	CCOR_00646	19.820648	41.702246	127.52837	1.6325307	2.9133509	9.69E-09	7.93E-31
GH92	CCOR_03689	8.7900567	17.389245	44.056897	1.0075634	1.7447566	0.0003762	3.35E-14
GH92	CCOR_00822	27.159682	21.730258	33.669623	0.2184896	1.0907039	0.4690659	2.26E-05
GH92	CCOR_03965	14.278235	6.9061117	19.697555	-0.494552	0.6895169	0.2150625	0.0043228
GH92	CCOR_10299	8.3546893	3.0356783	13.002863	-0.911609	0.8708918	0.0507055	0.0604128
GH92	CCOR_00912	22.744171	7.322705	18.072764	-1.245004	-0.105874	2.12E-05	0.780593
GT1	CCOR_00415	34.89851	49.530247	41.854144	1.2721922	0.4883562	3.35E-05	0.0714467

GT1	CCOR_06612	26.760873	6.289341	16.409687	-1.537408	-0.479008	3.13E-08	0.0661828
GT1	CCOR_09644	55.633199	10.976017	16.579917	-1.768592	-1.519381	3.06E-10	8.47E-11
GT1	CCOR_07216	4.5641467	0.2517333	0.2248017	-3.513284	-4.063216	2.26E-07	4.71E-19
GT1	CCOR_05146	7.104404	0.384434	8.1090183	-3.619743	0.4164685	1.40E-16	0.1110423
GT2	CCOR_05650	11.10626	19.843805	12.722251	1.4136883	0.4242375	0.0001339	0.1978353
GT2	CCOR_04921	10.023045	18.04936	7.4826647	1.4125845	-0.193745	0.0010765	0.6158643
GT25	CCOR_03766	23.95734	9.8793307	31.653527	-0.708701	0.630694	0.0633585	0.0183829
GT25	CCOR_09496	34.873071	11.265472	16.39166	-1.049874	-0.854653	0.0004075	0.0003691
GT3	CCOR_07315	13.676444	20.002827	23.845537	1.1131627	1.0264466	0.0001001	1.15E-05
GT31	CCOR_04305	23.585488	33.61921	34.929806	1.07928	0.796848	0.0003806	0.0012165
GT32	CCOR_00264	23.882495	67.832254	64.972632	2.0744665	1.6715419	6.31E-13	3.25E-13
GT32	CCOR_00977	29.294706	20.343948	55.107499	0.0315611	1.1083117	0.936769	3.97E-06
GT32	CCOR_10355	22.540187	2.5257003	2.9985213	-2.587426	-2.684528	0.0031342	0.0009428
GT34	CCOR_00623	1.07634	4.9303277	1.775993	2.7713698	0.955446	0.0001482	0.0271263
GT39	CCOR_07561	36.520082	94.866887	54.686534	1.9520993	0.8091962	4.88E-11	0.0008235
GT39	CCOR_00285	64.507232	106.17782	83.599843	1.1906747	0.5276912	8.74E-05	0.0444301
GT39	CCOR_02889	46.49152	68.056138	54.958693	1.12675	0.4685695	0.0001567	0.0639875
GT4	CCOR_00163	15.105328	28.039533	16.539017	1.4651074	0.3586993	3.22E-06	0.2158469
PL20	CCOR_09658	2.2747317	4.779435	14.951652	1.6048654	2.9363059	0.1849725	2.42E-15
PL27	CCOR_09544	10.467498	39.118915	24.491515	2.4719765	1.4528985	1.37E-17	5.28E-10
PL8	CCOR_10352	3.7568537	1.0263063	1.0579003	-1.267789	-1.598072	0.0260182	4.15E-05
AA1	CCM_08297	17.31613	38.204482	59.358685	1.2153057	1.5653322	1.969E-05	8.606E-09
AA1	CCM_02267	7.334825	4.7291593	15.287666	-0.565573	0.8373027	0.2273665	0.0257001
AA1	CCM_05132	4.981147	1.6516813	2.4456333	-1.519093	-1.249185	0.0018038	0.0129739
AA11	CCM_03237	23.95132	18.633638	11.820316	-0.299336	-1.250692	0.5155193	0.001166
AA12	CCM_05331	126.72756	11.975208	8.867467	-3.337024	-4.05819	9.142E-09	4.726E-11
AA2	CCM_01912	104.09059	131.48338	375.70867	0.3793009	1.5936906	0.3752507	1.165E-05
AA3	CCM_06376	804.02879	1107.6224	636.91903	0.5330427	-0.55619	0.3058211	0.2716919

AA3	CCM_01704	14.841678	14.189233	41.158551	-0.00111	1.2545131	0.9995828	1.86E-05
AA3	CCM_05125	158.10128	98.494558	43.919462	-0.610993	-2.068677	0.081871	1.076E-13
AA3	CCM_06092	152.83111	77.40694	54.576922	-0.792364	-1.642045	0.0250776	6.077E-07
AA3	CCM_00884	56.629149	25.441617	18.559144	-1.082682	-1.827894	3.225E-05	4.874E-13
AA5	CCM_06214	11.914313	6.296783	6.1198607	-0.850762	-1.179941	0.043711	0.0054737
AA6	CCM_06006	14.280216	28.91752	29.411581	1.087697	0.8222004	0.001265	0.0340963
AA7	CCM_04963	112.79039	73.191907	42.678533	-0.553959	-1.624475	0.0862254	1.295E-07
CBM21	CCM_09357	5.9084247	3.645618	2.584377	-0.630099	-1.420715	0.1716858	0.0010077
CE1	CCM_02120	23.509068	16.807641	47.80091	-0.41891	0.8092437	0.3782194	0.0373881
CE10	CCM_04420	0.169822	3.1755413	21.11115	4.2641892	6.7123425	6.914E-09	4.249E-32
CE10	CCM_09395	1.6669307	3.6495663	3.5647023	1.4692133	1.1075974	3.376E-05	0.0065021
CE10	CCM_07887	24.499533	53.073951	73.999439	1.1779727	1.3676612	2.285E-05	3.577E-07
CE10	CCM_02982	22.363778	41.988838	85.072741	0.9738302	1.709886	0.0007546	1.546E-11
CE10	CCM_04887	10.897051	11.340596	6.1999793	0.1269442	-1.033258	0.7444525	0.0018115
CE10	CCM_04930	11.043433	5.14364	3.8696913	-1.03055	-1.732467	0.0127546	4.183E-05
CE10	CCM_00456	8.7311357	3.239159	4.5857363	-1.360549	-1.153849	0.0024579	0.0073267
CE10	CCM_00143	20.955875	6.4786957	9.8628813	-1.628287	-1.307838	1.845E-07	0.0001041
CE10	CCM_05289	12.307763	3.7933007	12.550743	-1.629772	-0.200639	0.0014693	0.7652514
CE4	CCM_03626	1.443402	7.44198	4.4990007	2.4237755	1.4246596	8.783E-09	0.0043464
CE4	CCM_06324	9.1899523	27.575567	41.846385	1.5838343	1.896334	1.196E-09	2.43E-12
CE5	CCM_06273	1.063306	6.9805907	4.7961093	2.7647223	1.937953	4.94E-09	0.000185
CE5	CCM_00649	33.690056	65.578177	116.9706	1.0256646	1.5752561	0.000241	6.375E-10
CE5	CCM_01575	13.611999	5.4792173	11.455484	-1.240495	-0.464618	0.0078405	0.3855481
CE9	CCM_04622	1.9611243	7.4504197	28.379045	1.980333	3.570009	2.223E-06	1.346E-21
GH125	CCM_04607	128.88226	51.852756	51.274481	-1.247958	-1.575451	0.0001105	2.326E-06
GH128	CCM_04757	1140.2583	1115.1665	576.20797	0.0397131	-1.199825	0.9710638	0.0057669
GH13	CCM_09557	2105.6092	606.47394	190.07213	-1.595634	-3.588078	0.0007453	3.2E-11
GH135	CCM_03479	17.794485	28.009104	44.490738	0.7181612	1.1054994	0.0450675	0.0018425

GH142	CCM_03178	40.84072	14.825626	69.449178	-1.38837	0.5554495	0.0003301	0.0667224
GH15	CCM_08817	30.031964	45.654293	75.707753	0.6592778	1.1202342	0.0263766	6.707E-05
GH16	CCM_02110	14.849632	48.42518	128.02235	1.7748652	2.8905579	8.795E-11	2.558E-30
GH16	CCM_06466	8.5394417	27.098198	29.820298	1.7248678	1.5917347	7.029E-09	0.0052917
GH16	CCM_00948	86.753016	196.21126	173.82896	1.249697	0.7910922	5.265E-05	0.0088575
GH16	CCM_02336	27.215487	55.550757	51.124575	1.0963153	0.6885384	5.583E-05	0.0264974
GH16	CCM_07787	42.578451	54.08043	107.2225	0.4363458	1.1404388	0.6574467	0.0076615
GH16	CCM_02867	15.818788	17.102837	67.932007	0.1809476	1.8925153	0.75274	2.387E-08
GH16	CCM_04006	196.17175	173.29868	466.46691	-0.116354	1.0166613	0.8633128	0.007576
GH16	CCM_04992	1.981599	1.5102913	4.0909867	-0.324732	0.821459	0.6027147	0.0529281
GH16	CCM_05770	117.03708	75.124651	65.577838	-0.569625	-1.059714	0.1734945	0.0049587
GH16	CCM_05126	94.722638	34.84286	57.9983	-1.375017	-0.913958	0.0001255	0.016393
GH16	CCM_01469	10.588314	3.347945	4.00021	-1.59086	-1.622349	0.0022318	0.0020328
GH17	CCM_07328	265.32784	335.42658	168.24885	0.4064254	-0.872148	0.423587	0.0380568
GH18	CCM_08236	10.215027	4.9045927	3.2746133	-0.993717	-1.869291	0.0221324	7.181E-05
GH18	CCM_08922	18.481653	7.8041343	6.432957	-1.172934	-1.731795	0.0005163	1.419E-06
GH2	CCM_04640	9.550455	5.5090317	1.1349693	-0.77936	-3.398396	0.0080122	2.167E-28
GH2	CCM_04958	236.48436	117.87854	89.962774	-0.929881	-1.605242	0.0146468	4.257E-06
GH2	CCM_02883	331.32998	89.124819	799.26516	-1.826641	1.0688312	9.304E-07	0.0571144
GH20	CCM_09001	4.001522	7.482614	14.122108	0.9682362	1.5943399	0.030124	8.392E-05
GH25	CCM_08272	23.207572	31.638061	15.519289	0.5157888	-0.79856	0.1993676	0.0457301
GH28	CCM_08605	2.3200143	1.0221767	4.0388017	-1.11733	0.5704315	0.041153	0.2389024
GH29	CCM_09392	36.131676	13.4411	9.2771943	-1.360607	-2.186361	0.0430568	0.0016669
GH3	CCM_04729	27.432512	54.346653	34.311457	1.0574054	0.0990497	0.0012842	0.7540761
GH3	CCM_08719	3.707832	1.629096	1.4860937	-1.113602	-1.535335	0.0608863	0.0036326
GH3	CCM_00791	198.52923	86.593721	58.62382	-1.122899	-1.97348	0.006083	5.18E-06
GH31	CCM_03514	76.711754	44.019596	37.491437	-0.731114	-1.251101	0.0161593	3.866E-06
GH32	CCM_00448	11.654347	5.7302693	6.0201613	-0.95301	-1.171223	0.0142132	0.0042304

GH35	CCM_03330	2.8583517	1.3640473	8.3613893	-0.987928	1.3488196	0.0890323	0.000941
GH37	CCM_08788	8.1341197	6.1499287	63.592766	--	2.8160313	--	1.468E-07
GH37	CCM_05551	8.8103597	42.618062	84.299199	2.3402435	3.038587	2.253E-18	1.527E-32
GH5	CCM_00770	0.0806363	0.321945	0.9143153	--	3.1327168	--	7.941E-07
GH54	CCM_05741	445.31847	379.57897	58.281986	-0.1613	-3.157999	0.7253224	1.496E-16
GH55	CCM_02756	5.2941497	5.4749147	25.539828	0.123086	2.0641988	0.8669718	9.003E-11
GH72	CCM_04426	61.842974	82.057139	39.518166	0.4819309	-0.866536	0.1285307	0.0016067
GH72	CCM_00215	58.562101	74.909665	38.653726	0.4273758	-0.816926	0.2950375	0.0073544
GH72	CCM_04709	52.748135	50.65926	233.21333	0.3101955	1.130975	0.4546184	0.0028428
GH75	CCM_04645	17.511136	49.132072	58.307608	1.5123187	1.4525735	1.587E-07	4.727E-06
GH76	CCM_08571	1.7163107	10.441325	1.5302253	2.6635116	-0.380527	3.335E-10	0.5874207
GH76	CCM_03213	14.226009	21.128095	40.339347	0.6383616	1.2789994	0.0754126	2.82E-05
GH76	CCM_01968	402.23888	478.32891	321.49213	0.3168771	-1.032629	0.7041641	0.2885042
GH76	CCM_04240	5.5563157	4.4735253	25.713294	-0.25083	2.017004	0.6044277	1.069E-13
GH76	CCM_07600	5.912343	3.6403557	9.4028413	-0.630495	0.4537773	0.0469225	0.1374327
GH84	CCM_02676	3.6021063	1.7178127	1.5100313	-0.996302	-1.480818	0.0477726	0.0043988
GH88	CCM_07984	8.07984	12.169004	24.090611	0.6556835	1.3672862	0.1187581	0.0003135
GH89	CCM_04090	1.2050503	1.5727383	11.090959	0.449852	2.9777257	0.4364103	1.976E-15
GH92	CCM_01417	188.53568	107.4857	60.078077	-0.736418	-1.862952	0.0971282	3.063E-05
GH92	CCM_02086	115.20331	29.964766	99.5562	-1.870874	-0.418928	0.0003728	0.3150274
GT1	CCM_01158	10.277253	11.076442	39.814554	0.1773608	1.7413114	0.7047758	6.983E-12
GT1	CCM_09564	2.1225907	0.893987	4.3518567	-1.19299	0.8146443	0.0622659	0.1416003
GT15	CCM_05618	11.530431	10.975859	39.586598	-0.013919	1.5688284	0.9870034	0.0004183
GT17	CCM_01490	31.094952	97.233251	137.53526	1.7152617	1.9350474	7.95E-12	1.499E-14
GT17	CCM_03026	66.05581	108.2299	163.8439	0.7845881	1.094797	0.0055592	3.087E-05
GT2	CCM_00949	15.820438	35.781205	55.459728	1.244645	1.5953881	0.013691	3.152E-08
GT2	CCM_05447	20.242294	40.134046	37.753633	1.0612589	0.687636	5.577E-05	0.0125484
GT2	CCM_00447	4.8675207	8.302447	16.039264	0.840475	1.5041116	0.0322418	0.0001131

GT2	CCM_06975	2.4162453	2.3044857	10.463917	-0.000962	1.8888803	0.9926923	6.309E-05
GT2	CCM_02966	38.397827	36.303324	88.443077	-0.009906	0.9888527	0.9580813	0.0051416
GT2	CCM_08517	2.8864067	2.2159383	9.57206	-0.31237	1.5123953	0.777777	0.0043296
GT20	CCM_06851	84.9664	51.492076	34.240056	-0.648875	-1.533016	0.0690025	4.106E-06
GT21	CCM_09527	6.1734803	4.328676	2.1252277	-0.363175	-1.808053	0.7054271	0.0028168
GT22	CCM_02670	3.9971173	4.6143743	13.498685	0.2798148	1.5457472	0.6653404	0.0001607
GT25	CCM_08718	3.5642643	9.1468457	5.9543077	1.4233683	0.5263066	2.124E-05	0.2857845
GT32	CCM_08824	23.459565	10.347044	42.478383	-1.099477	0.653361	0.1728885	0.1399882
GT32	CCM_05218	4.1739707	1.3660897	2.6399793	-1.547383	-0.880134	0.0005375	0.0647252
GT4	CCM_02472	18.242071	30.648106	44.454785	0.8193642	1.0736942	0.0071502	0.000169
GT62	CCM_06375	3.139237	0.152263	1.0254567	-4.269079	-1.788269	7.056E-17	4.148E-05
GT64	CCM_02315	0.2492293	0.4611153	2.4717567	--	3.0511011	--	1.757E-07
GT66	CCM_06369	52.060267	82.230573	123.14053	0.7286909	1.0277208	0.0585617	0.0064486
GT90	CCM_05631	13.061758	14.280321	30.862181	0.1974556	1.0184018	0.6621352	0.0002633
GT90	CCM_01090	70.767606	55.799207	37.333609	-0.269869	-1.145278	0.4768754	1.543E-05
PL35	CCM_03529	8.934408	9.559947	5.518669	0.1620782	-0.920558	0.7359812	0.0354589

Table S11 GH18 genes in *Cordyceps militaris*.

CAZyme classification	Gene ID	CM_fpkm	4 dpi_fpkm	8 dpi_fpkm	4 dpi vs CM_log2FC	8 dpi vs CM_log2FC	4 dpi vs CM_FDR	8 dpi vs CM_FDR
GH18	CCM_00318	5.8434663	6.488753	13.263693	0.5887234	0.2225097	0.0007941	0.9654879
GH18	CCM_00485	0.5992523	0.3546713	0.2200173	--	--	--	--
GH18	CCM_03279	7.65143	4.876818	6.4819283	0.2858528	-0.583354	0.4192103	-0.469754
GH18	CCM_03587	0	0	0	--	--	--	--
GH18	CCM_03652	0.2731563	0.3548317	0.6131457	--	--	0.2720191	0.7451595
GH18	CCM_04817	0.1208963	0.0947343	0.1262533	--	--	--	--
GH18	CCM_05117	841.67894	863.58852	1111.7562	0.8293606	0.1435992	0.8026938	0.1678622
GH18	CCM_05689	39.218196	0	0	--	--	--	--
GH18	CCM_05745	2.81086	1.7668443	1.5632927	0.3964085	-0.604684	0.0605885	-1.053663
GH18	CCM_06072	2.1388433	2.1514953	3.747042	--	--	0.407147	0.5842829
GH18	CCM_07123	11.944839	9.1530233	8.2033327	0.5887822	-0.312236	0.1093028	-0.76079
GH18	CCM_07460	0	0	10.303017	--	--	--	--
GH18	CCM_07476	34.938585	21.605226	27.409118	0.0904031	-0.624175	0.1562667	-0.567803
GH18	CCM_08025	0	0	0	--	--	--	--
GH18	CCM_08177	19.658963	22.027689	21.828281	0.5207277	0.2339456	0.8709858	-0.06625
GH18	CCM_08236	10.215027	4.9045927	3.2746133	0.0221324	-0.993717	7.181E-05	-1.869291
GH18	CCM_08848	2.5289597	2.1339797	3.5701623	0.9692597	0.0470205	0.3796628	0.4480156
GH18	CCM_08922	18.481653	7.8041343	6.432957	0.0005163	-1.172934	1.419E-06	-1.731795
GH18	CCM_09128	0.161261	0.193939	0.44699	--	--	--	--
GH18	CCM_09133	16.30937	12.880942	16.270336	0.5389125	-0.265494	0.6548259	-0.213657
GH18	CCM_09134	11.88455	15.871543	25.167233	0.1569474	0.4932649	0.0035494	0.8527688
GH18	CCM_09185	103.98846	112.15359	125.89918	0.6128099	0.1749772	0.886942	0.0568099

Table S12 Core gene in *Calcarisporium cordycipiticola* (CC) and *Cordyceps militaris* (CM).

Species	Cluster number	Type	Most similar known cluster	Similarity	Gene ID	CC/CM_fpkm	4 dpi_fpkm	8 dpi_fpkm	4 dpi vs CC/4 dpi vs CM_log2FC	8 dpi vs CC/4 dpi vs CM_log2FC	4 dpi vs CC/4 dpi vs CM_FDR	8 dpi vs CC/8 dpi vs CM_FDR
CC	Cluster 1	NRPS	Oxaleimide C	0.1	CCOR_00011	4.3108797	0.7773533	3.3889347	-1.927394	-0.097464	7.75E-07	0.8615127
CC	Cluster 2	NRPS-like			CCOR_00026	2.4089407	0.2370927	10.755294	-2.701048	2.3854316	0.0002689	2.40E-19
CC	Cluster 3	NRPS			CCOR_00072	3.1906977	1.7510423	4.3421757	-0.286983	0.668846	0.4597454	0.00681
CC	Cluster 4	NRPS-like			CCOR_00263	6.7495543	7.5563953	31.360483	0.5002532	1.6521205	0.0979148	5.92E-13
CC	Cluster 5	T1PKS			CCOR_00606	0.3940103	0.0746317	0.5614637	-1.702071	0.7365673	0.0102592	0.0915458
CC	Cluster 6	NRPS	Dimethylcoprogen	1	CCOR_00729	10.709652	71.780731	146.99878	3.3181513	4.0080524	9.24E-30	2.31E-55
CC	Cluster 6	NRPS	Dimethylcoprogen	1	CCOR_00730	6.7141103	39.240594	35.270165	3.1248006	2.6182331	5.42E-20	2.02E-28
CC	Cluster 7	NRPS			CCOR_00896	79.898013	51.796577	50.94407	-0.059484	-0.427757	0.8855371	0.2843408
CC	Cluster 8	T1PKS,NRPS	Pyranonigrin E	1	CCOR_01013	3.8472487	2.0076537	4.9852497	-0.365926	0.6006833	0.2699954	0.0146792
CC	Cluster 9	T1PKS,NRPS			CCOR_01067	2.8069193	0.4612847	1.121402	-2.051029	-1.097722	2.95E-08	2.50E-05
CC	Cluster 10	NRPS-like			CCOR_01465	25.769001	16.702039	18.42585	-0.052534	-0.259034	0.9142647	0.3704487
CC	Cluster 11	T1PKS			CCOR_01537	2.341706	2.6507663	2.428761	0.7404604	0.2871652	0.0932318	0.3997709
CC	Cluster 12	NRPS	Apicidin	0.18	CCOR_01636	0.238879	0.075478	0.283662	-1.024763	0.469539	0.194251	0.3435292
CC	Cluster 13	NRPS			CCOR_01714	0.0011483	3.5496563	4.507634	--	11.686742	--	3.68E-66
CC	Cluster 14	NRPS			CCOR_01762	0.004119	0.0304717	0.0309097	--	--	--	--
CC	Cluster 15	NRPS	Aureobasidin A1	1	CCOR_01788	0.8923477	0.0633543	0.4250897	-3.228922	-0.843509	6.75E-15	0.0025351
CC	Cluster 16	NRPS-like,T1PKS			CCOR_01802	48.663972	4.8394327	28.0942	-2.764679	-0.566241	5.14E-10	0.0721377
CC	Cluster 16	NRPS-like,T1PKS			CCOR_01803	52.772303	4.0499203	30.878766	-3.19165	-0.551042	1.67E-12	0.086017
CC	Cluster 17	T1PKS			CCOR_01813	6.5244273	1.5215033	3.7616097	-1.532587	-0.568968	7.51E-07	0.0278144
CC	Cluster 18	indole			CCOR_01834	5.9115347	1.383548	4.1221597	-1.52629	-0.295519	0.0015509	0.4364836
CC	Cluster 19	T1PKS			CCOR_01967	4.1607797	2.6935477	3.603054	0.0127275	0.0662923	0.9304744	0.8774006
CC	Cluster 20	terpene			CCOR_02130	43.154054	59.369897	41.773214	1.0319256	0.1797517	0.0004536	0.5087567
CC	Cluster 21	NRPS-like			CCOR_02161	9.386554	2.9818977	6.0848917	-1.091757	-0.400207	0.0049026	0.3669116

CC	Cluster 22	T1PKS			CCOR_02686	1.875077	1.441089	1.7824507	0.1928662	0.153286	0.7287749	0.6668532
CC	Cluster 22	T1PKS			CCOR_02691	4.2807963	0.2251093	5.5406647	-3.687842	0.6051391	0.0266841	0.4946309
CC	Cluster 23	indole,T1PKS	Neurosporin A	0.33	CCOR_02928	1.1458877	0.1143383	0.5921133	-2.711817	-0.72293	7.34E-06	0.0639875
CC	Cluster 24	T1PKS	Neurosporin A	0.2	CCOR_03499	47.475948	8.9241597	6.7877363	-1.896592	-2.579367	0.0146285	0.000332
CC	Cluster 25	NRPS			CCOR_03543	3.588753	7.6047613	5.683736	1.652648	0.8876891	1.56E-08	0.0026442
CC	Cluster 26	T1PKS	Asperfuranone	0.18	CCOR_03606	2.2348047	0.3637293	0.8202113	-2.063327	-1.216736	5.57E-06	7.67E-05
CC	Cluster 26	T1PKS	Asperfuranone	0.18	CCOR_03607	1.8014973	0.151109	1.0169793	-3.005375	-0.598985	3.43E-08	0.0979097
CC	Cluster 27	T1PKS,NRPS	Fusarielin H	0.25	CCOR_03646	4.959034	7.0086797	5.609803	1.0773969	0.4027754	0.0233409	0.2873534
CC	Cluster 28	NRPS			CCOR_03665	41.8266	32.249534	78.887271	0.1967064	1.14141	0.8062798	0.0321022
CC	Cluster 29	NRPS-like			CCOR_03834	59.222772	66.474476	112.46435	0.7149289	1.1518028	0.3115025	0.0030089
CC	Cluster 30	NRPS-like			CCOR_03887	0.3064127	0.013615	0.2245387	--	--	--	--
CC	Cluster 31	NRPS			CCOR_03955	9.2615823	8.514405	23.956554	0.4317044	1.5992401	0.2809397	7.44E-11
CC	Cluster 31	NRPS			CCOR_03957	0.143075	0	0.0550587	--	-1.135042	2.48E-11	0.0147223
CC	Cluster 32	T1PKS			CCOR_04046	0.1282413	0.0570677	0.059929	--	--	--	--
CC	Cluster 33	T1PKS			CCOR_04255	323.45127	555.04749	429.4566	1.3429029	0.6374861	6.74E-06	0.0173277
CC	Cluster 34	NRPS			CCOR_04278	0.060568	0.0239877	0.073064	--	--	--	--
CC	Cluster 35	NRPS-like,T1PKS			CCOR_04335	0.7973203	0.019501	0.3085083	-4.385139	-1.130042	1.52E-07	0.0196919
CC	Cluster 35	NRPS-like,T1PKS			CCOR_04336	0.5172263	0.20567	1.0736243	-0.791646	1.2814067	0.4191545	0.0014726
CC	Cluster 36	NRPS			CCOR_05013	7.445516	3.7221413	6.4918733	-0.432766	0.0285194	0.1430137	0.9718426
CC	Cluster 37	NRPS,T1PKS,terpene			CCOR_05051	1.6011553	0.2514567	1.4131157	-2.077288	0.0491952	0.0009596	0.9160046
CC	Cluster 37	NRPS,T1PKS,terpene			CCOR_05052	4.985006	1.4560293	2.6636687	-1.184933	-0.671873	0.080916	0.0823201
CC	Cluster 37	NRPS,T1PKS,terpene			CCOR_05054	0.7031723	0.1694093	1.2167873	-1.470486	1.0225084	0.0326533	0.1220976
CC	Cluster 37	NRPS,T1PKS,terpene			CCOR_05055	1.2023103	0.291966	0.8816563	-1.470833	-0.216833	0.0118826	0.5719634
CC	Cluster 38	T1PKS			CCOR_05213	8.9099547	10.437013	16.392601	0.7677228	1.1100589	0.0273925	2.80E-06
CC	Cluster 39	T1PKS	Neurosporin A	0.26	CCOR_05235	0.5769437	0.391661	0.4407033	0.0540075	-0.164671	1	0.8253145
CC	Cluster 40	T1PKS,NRPS	Botryenalol / botrydial / dihydrobotrydial / botryendial / beta-O-	1	CCOR_05312	2.85448	0.7058497	1.4902767	-1.446851	-0.71332	0.0011029	0.0200605

			Methyl-dihydrobotrydialone / botcinic acid / botcinin A									
CC	Cluster 40	T1PKS,NRPS	Botryenalol / botrydial / dihydrobotrydial / botryendial / beta-O-Methyl-dihydrobotrydialone / botcinic acid / botcinin A	1	CCOR_05320	0.638939	0.3879227	0.454401	-0.175297	-0.265256	0.9738135	0.6407013
CC	Cluster 40	T1PKS,NRPS	Botryenalol / botrydial / dihydrobotrydial / botryendial / beta-O-Methyl-dihydrobotrydialone / botcinic acid / botcinin A	1	CCOR_05324	0.431633	2.134266	1.6587837	2.8774741	2.1661305	1.49E-13	2.14E-09
CC	Cluster 41	T1PKS			CCOR_05354	1.712636	0.205623	0.5896943	-2.396136	-1.257544	2.51E-06	0.0001543
CC	Cluster 42	T1PKS,NRPS-like			CCOR_05397	0.0029753	0	0	--	--	--	--
CC	Cluster 42	T1PKS,NRPS-like			CCOR_05400	5.6786613	0.7895703	5.3776247	-2.271968	0.145373	1.15E-06	0.6442847
CC	Cluster 43	fungal-RiPP			CCOR_05413	40.717374	21.862861	62.834939	-0.342176	0.8542635	0.3594397	0.0002496
CC	Cluster 43	fungal-RiPP			CCOR_05418	5.3244493	4.8524827	7.4958593	0.4435033	0.7253849	0.483554	0.0525397
CC	Cluster 44	terpene	Copalyl diphosphate	0.28	CCOR_05686	2.0677203	0.176294	2.2250097	-2.844758	0.3359941	1.11E-05	0.4920226
CC	Cluster 45	terpene			CCOR_06225	93.323893	74.172165	97.336116	0.24005	0.2888936	0.4709569	0.2879056
CC	Cluster 46	T1PKS	Depudecin	0.5	CCOR_06814	0.1576853	0.024319	0.1918097	-1.918491	0.5087806	0.0185758	0.3467684
CC	Cluster 47	T1PKS			CCOR_06829	0.1583357	4.6928043	3.2811187	5.4506856	4.575834	3.43E-35	2.68E-06
CC	Cluster 49	terpene	Clavaric acid	1	CCOR_07026	25.699979	19.480595	23.69934	0.1716229	0.112236	0.6366152	0.7215866
CC	Cluster 50	T1PKS,NRPS	Radicicol	1	CCOR_07359	0.3090317	0.025055	0.0832817	-2.909139	-1.650402	0.0004537	0.0010982
CC	Cluster 50	T1PKS,NRPS	Radicicol	1	CCOR_07363	0.1613917	0.0245107	0.149696	-1.81369	0.124731	0.0230053	0.8276934
CC	Cluster 50	T1PKS,NRPS	Radicicol	1	CCOR_07369	0.6164807	0.0808823	0.9708383	-2.376057	0.8806181	0.0004144	0.011998
CC	Cluster 51	T1PKS	Citrinin	0.18	CCOR_07571	4.691381	0.0180087	0.157838	-7.292128	-4.662806	6.98E-14	2.18E-09

CC	Cluster 52	NRPS-like			CCOR_07643	2.3169103	0.1336153	2.0152553	-3.420132	0.0321154	0.1263917	0.9443341
CC	Cluster 53	NRPS-like			CCOR_07791	0.1607037	0.030143	0.0967923	--	--	--	--
CC	Cluster 54	NRPS,T1PKS	Fusarielin H	0.25	CCOR_07890	18.040047	8.481526	18.765279	-0.51619	0.2823738	0.1294903	0.28684
CC	Cluster 55	T1PKS	Citrinin	0.18	CCOR_07909	212.89985	1.2531233	3.0470587	-6.865441	-5.90078	4.23E-91	9.00E-66
CC	Cluster 56	T1PKS,NRPS-like	Swainsonine	0.28	CCOR_07947	12.348507	0.0109327	0.0291067	-9.305467	-8.4396	0.000966	0.0011285
CC	Cluster 57	NRPS			CCOR_07994	0.306747	0	0	--	--	--	--
CC	Cluster 57	NRPS			CCOR_08002	0.1324133	0.032691	0.1188577	--	0.0737335	--	0.9267021
CC	Cluster 48	NRPS-like			CCOR_08319	0.58591	0.5952417	0.1000543	--	--	--	--
CC	Cluster 58	T1PKS,NRPS	Curvupallides	0.11	CCOR_09394	0.7130873	0.1114283	0.6120577	-1.724388	0.2400922	0.0117678	0.597852
CC	Cluster 59	T1PKS			CCOR_09635	0.1161143	0	0.0088407	--	--	--	--
CC	Cluster 60	NRPS-like			CCOR_09651	14.567303	1.2396993	14.163766	-2.993169	0.18554	4.92E-19	0.498121
CC	Cluster 61	NRPS-like			CCOR_09742	1.2504237	0.299645	0.945584	-1.442431	-0.186706	0.0481976	0.9564392
CC	Cluster 62	NRPS,T1PKS	Squalestatin S1	0.09	CCOR_09935	0.4377713	0.1125663	1.4655757	--	1.9542965	--	0.0077203
CC	Cluster 63	NRPS-like			CCOR_10045	5.5749353	2.299106	3.9675667	-0.708947	-0.262444	0.0625088	0.3821108
CC	Cluster 64	NRPS,terpene			CCOR_10244	4.0493303	3.64858	4.423391	0.4109943	0.34845	0.22325	0.5999754
CC	Cluster 65	T1PKS	Phomopsins	1	CCOR_10268	0.0479297	0.0188993	0.0098057	--	--	--	--
CC	Cluster 66	T1PKS,NRPS			CCOR_10417	0.6220973	1.0210347	4.5681343	1.2898668	3.0965677	0.0066425	2.83E-07
CM	Cluster 1	T1PKS	Nivalenol / deoxynivalenol / 3-acetyldeoxynivalenol / 15-acetyldeoxynivalenol / neosolaniol / calonectrin / apotrichodiol / isotrichotriol / 15-decalonectrin / T-2 Toxin / 3-acetyl T-2 toxin / trichodiene	8%	CCM_00603	0.2692857	0.069956	0.1566407	-1.863306	-0.999348	0.0014895	0.0987855
CM	Cluster 2	NRPS			CCM_00621	14.23804	8.4869663	20.793585	-0.67704	0.3188677	0.064174	0.4301283
CM	Cluster 3	NRPS			CCM_00713	9.1841607	5.3635387	7.6561737	-0.711682	-0.485843	0.1507278	0.35785

CM	Cluster 3	NRPS			CCM_00722	1.3885273	2.6950203	3.5817963	1.0257237	1.1464236	0.0206924	0.0070266
CM	Cluster 4	NRPS,T1PKS	Emericellamide A / emericellamide B	60%	CCM_01282	4.7843597	3.6055583	9.0883323	-0.336816	0.7129654	0.3864656	0.0167774
CM	Cluster 4	NRPS,T1PKS	Emericellamide A / emericellamide B	60%	CCM_01284	0.4654267	5.1599683	20.227628	3.5156895	5.2175506	0.0265334	1.46E-35
CM	Cluster 4	NRPS,T1PKS	Emericellamide A / emericellamide B	60%	CCM_01285	1.058745	0.969521	2.8363403	-0.058984	1.2071882	0.9642853	0.0007432
CM	Cluster 5	T1PKS			CCM_01518	0.115717	0.0339787	0.054446	--	--	--	--
CM	Cluster 6	NRPS			CCM_01705	2.6827927	3.1347327	5.2814157	0.2892033	0.7592585	0.4466159	0.0115627
CM	Cluster 7	T1PKS	Cercosporin	100%	CCM_01921	2.736407	2.33327	1.4304637	-0.318248	-1.314968	0.4393773	0.0009455
CM	Cluster 8	fungal-RiPP	Ustiloxin B	36%	CCM_02059	10.641917	6.116101	1.1866583	-0.735569	-3.372315	0.5624831	0.0124223
CM	Cluster 8	fungal-RiPP	Ustiloxin B	36%	CCM_02060	0.495103	0.159964	0.0833073	--	--	--	--
CM	Cluster 8	fungal-RiPP	Ustiloxin B	36%	CCM_02061	0.0522777	0.000124	0.058084	--	--	--	--
CM	Cluster 8	fungal-RiPP	Ustiloxin B	36%	CCM_02065	14.163469	12.020684	9.253311	-0.158483	-0.82056	0.6471955	0.0013696
CM	Cluster 9	T1PKS			CCM_02374	0.7068423	0.175913	0.311639	-1.931844	-1.391036	0.0001227	0.0053796
CM	Cluster 10	NRPS			CCM_02437	4.6278433	1.7005727	2.3468263	-1.373237	-1.196512	0.0002031	0.0025469
CM	Cluster 11	NRPS-like			CCM_02691	33.080881	41.76066	31.11305	0.4059854	-0.30541	0.231001	0.3340692
CM	Cluster 12	terpene	PR toxin	50%	CCM_03050	0.0422023	0	0.0301967	--	--	--	--
CM	Cluster 13	NRPS			CCM_03255	2.0428663	1.2653463	1.5845473	-0.619596	-0.586086	0.105826	0.1723857
CM	Cluster 14	NRPS-like			CCM_03390	20.49698	19.059702	25.398435	-0.036373	0.0908469	0.9272836	0.8034752
CM	Cluster 15	T1PKS	Elsinochrome A	100%	CCM_03663	14.89853	11.477554	8.4175403	-0.417468	-1.085712	0.1915455	1.44E-05
CM	Cluster 16	terpene	Elsinochrome A	100%	CCM_03697	69.257394	71.817385	67.893809	0.1194622	-0.249687	0.7524494	0.4457679
CM	Cluster 17	NRPS,T1PKS			CCM_04722	0.165978	0.196013	0.3651277	0.3018966	0.921794	0.6527935	0.0983634
CM	Cluster 17	NRPS,T1PKS			CCM_04725	4.9409187	5.544209	7.5933597	0.2297376	0.3943608	0.5671159	0.316549
CM	Cluster 18	NRPS-like			CCM_05078	3.6599397	1.8967327	2.6885267	-0.876377	-0.666174	0.0436257	0.1606798
CM	Cluster 19	NRPS			CCM_05307	2.92943	1.966676	2.4098753	-0.504539	-0.497601	0.3477433	0.3263017
CM	Cluster 20	NRPS	Dimethylcoprogen	100%	CCM_06701	4.367122	6.841284	7.432348	0.7164272	0.5624619	0.3066	0.5499282
CM	Cluster 20	NRPS	Dimethylcoprogen	100%	CCM_06702	4.7879097	3.4965247	3.1406397	-0.383564	-0.821615	0.3330017	0.0313117

CM	Cluster 21	NRPS	Aureobasidin A1	100%	CCM_07302	9.380108	6.7022743	7.3402507	-0.458298	-0.710038	0.1796798	0.0320483
CM	Cluster 22	terpene			CCM_07634	53.856689	77.220698	76.559288	0.5903198	0.2922706	0.0498365	0.3490937
CM	Cluster 23	T1PKS,NRPS			CCM_08016	4.752719	2.7877757	4.287298	-0.700743	-0.367522	0.0420682	0.3561972
CM	Cluster 23	T1PKS,NRPS			CCM_08018	0.2578443	0.2201463	0.3621533	-0.167372	0.2694302	0.8756694	0.6835723
CM	Cluster 24	NRPS,T1PKS	Fumosorinone	83%	CCM_08261	6.01187	9.9375583	17.11046	0.7859456	1.2791979	0.0053541	1.93E-06
CM	Cluster 25	NRPS-like			CCM_08331	8.1754553	123.64492	131.65795	3.9922795	3.7978797	1.86E-33	5.09E-31
CM	Cluster 26	NRPS-like			CCM_08730	7.3203193	5.5388707	6.5099973	-0.34043	-0.383589	0.3230402	0.198622
CM	Cluster 27	NRPS-like			CCM_08771	18.980465	31.006879	20.459579	0.7753944	-0.120651	0.0046878	0.8748334
CM	Cluster 28	T1PKS			CCM_09042	106.80284	11.859124	9.3834583	-3.10535	-3.72348	1.36E-11	9.53E-15
CM	Cluster 29	NRPS,T1PKS			CCM_09341	3.5778633	1.8315043	2.090693	-0.900571	-0.999106	0.0328882	0.021943
CM	Cluster 29	NRPS,T1PKS			CCM_09346	0.1614207	0.4056133	1.5396947	1.355224	3.007941	0.5570797	1.34E-05
CM	Cluster 30	terpene	Clavaric acid	100%	CCM_09526	46.987684	28.63436	31.609418	-0.643792	-0.788454	0.0246848	0.0076413

Table S13 Siderophore synthesis related gene clusters in *Calcarisporium cordycipiticola* (CC) and *Cordyceps militaris* (CM).

Species	Gene ID	CC/CM_fpkm	4 dpi_fpkm	8 dpi_fpkm	4 dpi vs CC/4 dpi vs CM_log2FC	8 dpi vs CC/4 dpi vs CM_log2FC	4 dpi vs CC/4 dpi vs CM_FDR	8 dpi vs CC/8 dpi vs CM_FDR
CC	CCOR_00724	16.67266933	17.647939	26.884399	0.665029229	0.926209115	0.669157235	0.320893384
CC	CCOR_00725	0.218702667	17.439137	0.6126517	6.873509359	1.704655673	7.23E-46	0.041670714
CC	CCOR_00726	67.012061	278.28196	285.91552	2.628262604	2.319751422	4.77E-18	8.57E-20
CC	CCOR_00727	12.89356333	111.57242	159.51713	3.680777285	3.856584734	8.18E-37	3.87E-51
CC	CCOR_00728	42.423611	253.91926	236.04757	3.154688714	2.702843718	9.70E-25	1.64E-25
CC	CCOR_00729	10.70965167	71.780731	146.99878	3.318151291	4.008052366	9.24E-30	2.31E-55
CC	CCOR_00730	6.714110333	39.240594	35.270165	3.124800565	2.618233081	5.42E-20	2.02E-28
CC	CCOR_00731	8.803260333	5.6939267	10.486598	-0.076657317	0.480348044	0.98277364	0.160523878
CC	CCOR_00732	0.411281667	0.2797043	0.5952777	--	0.760959658	--	0.147317355
CC	CCOR_00733	29.741816	22.977175	22.318635	-0.008895435	-0.162958926	0.956897563	0.6035774
CC	CCOR_00734	40.09741833	31.5191	37.539263	0.222068091	0.135522555	0.529766988	0.686177145
CC	CCOR_00735	3.722678667	1.6438217	3.228785	-0.620741344	0.022358798	0.299971398	0.949490227
CC	CCOR_00736	61.80241733	75.242941	67.424946	0.620871963	0.310641126	0.039661372	0.238052173
CC	CCOR_00737	26.003261	44.246007	19.349846	1.339969849	-0.198560334	2.62E-05	0.500662965
CM	CCM_06695	7.771594	6.6891323	10.252148	-0.148933495	0.185096763	0.811564501	0.752061225
CM	CCM_06696	34.45024467	42.067829	60.575778	0.357721914	0.603916871	0.292460835	0.041448823
CM	CCM_06697	11.723194	12.130128	11.800273	0.1214705	-0.199327511	0.79838414	0.529946836
CM	CCM_06698	80.95054633	50.829558	29.74775	-0.601716488	-1.656147598	0.033140865	2.67E-11
CM	CCM_06699	0.986613	1.4197317	1.1475317	0.58559533	--	0.448871456	--
CM	CCM_06700	61.00826167	56.665244	41.410413	-0.037728277	-0.778874464	0.925777301	0.022593291
CM	CCM_06701	4.367122	6.841284	7.432348	0.716427161	0.56246189	0.3066	0.549928167
CM	CCM_06702	4.787909667	3.4965247	3.1406397	-0.383563512	-0.821615167	0.333001732	0.031311671
CM	CCM_06703	5.160271333	6.2532647	18.00691	0.337195887	1.603169116	0.736379292	0.001263737
CM	CCM_06704	14.73592933	17.339974	13.735623	-0.361063605	-0.318188639	0.423896932	0.313399437

CM	CCM_06705	60.167418	45.199075	65.759974	-0.345361309	-0.085747722	0.295027995	0.798723043
CM	CCM_06706	2.869962333	3.558753	4.6689003	0.376988969	0.487386919	0.451282817	0.315572594
CM	CCM_06707	86.395968	96.845383	115.5263	0.201324967	0.174127832	0.538804282	0.629538721

Table S14 ROS- related genes in *Cordyceps militaris* (CM) and *Calcarisporium cordycipiticola* (CC).

Species	Annotation	Gene ID	CC/CM_fpkm	4 dpi_fpkm	8 dpi_fpkm	4 dpi vs CC/4 dpi vs CM_log2FC	8 dpi vs CC/4 dpi vs CM_log2FC	4 dpi vs CC/4 dpi vs CM_FDR	8 dpi vs CC/8 dpi vs CM_FDR
CM	catalase	CCM_02506	6.3676057	5.1000513	5.819448	-0.2538483	-0.3491107	0.6469155	0.487815
CM	catalase	CCM_07732	7.0153937	4.1643253	5.7016097	-0.6802222	-0.5184961	0.1277933	0.2918333
CM	cytochrome oxidase	CCM_01362	14.565938	18.719665	28.23865	0.4239262	0.7330005	0.4210032	0.118165
CM	cytochrome oxidase	CCM_04541	72.686955	116.19567	90.388826	0.4277301	-0.1681616	0.1568793	0.6549584
CM	cytochrome oxidase	CCM_05057	15.4847	30.201708	34.779936	1.0293555	0.9475335	0.0007503	0.0039659
CM	cytochrome oxidase	CCM_05315	23.997817	31.933203	33.01598	0.4821404	0.2439553	0.2055078	0.6111995
CM	cytochrome oxidase	CCM_06413	18.566327	16.03999	16.946753	-0.1412933	-0.3465285	0.8208453	0.4854815
CM	cytochrome oxidase	CCM_08896	26.007138	32.177931	35.836414	0.374138	0.2445921	0.385946	0.6172586
CM	*glutaredoxin*	CCM_06629	84.937156	107.82322	91.578731	0.414127	-0.1083498	0.2546584	0.7871271
CM	glutaredoxin	CCM_07003	64.705232	70.503191	55.874479	0.1923785	-0.4319895	0.5672958	0.1585858
CM	glutaredoxin	CCM_07058	8.5273333	9.736698	10.12607	0.2600048	0.0280914	0.7273197	0.9692346
CM	glutaredoxin	CCM_09072	73.999953	55.48375	71.548744	-0.3624712	-0.3161287	0.294662	0.4300709
CM	glutaredoxin	CCM_09582	69.431661	67.416669	63.398647	0.0257185	-0.3469628	0.9591004	0.3786244
CM	glutathione peroxidase	CCM_01615	1.2947807	2.2604847	3.1891367	0.8602771	1.0746754	0.196077	0.071614
CM	glutathione peroxidase	CCM_03086	169.7123	142.8242	135.34714	-0.1172634	-0.4741507	0.8034936	0.1710237
CM	glutathione peroxidase	CCM_05274	0.1986367	0.229933	0.387508	--	--	--	--
CM	glutathione reductase	CCM_09201	68.798842	84.557383	86.777758	0.3647908	0.1201612	0.2687071	0.7876712
CM	NADPH oxidase	CCM_01316	37.61355	39.784727	44.304737	0.1492403	0.016022	0.6870094	0.9389349
CM	NADPH oxidase	CCM_01357	132.56395	268.8731	282.88902	1.102871	0.8861028	0.0170839	0.0973396
CM	NADPH oxidase	CCM_08161	18.931064	9.8224247	17.941001	-0.8151433	-0.1830634	0.0100034	0.6775908
CM	peroxidase	CCM_01398	1175.2406	664.07085	384.712	-0.6692207	-1.7022987	0.1787105	0.0003067

CM	peroxidase	CCM_01912	104.09059	131.48338	375.70867	0.3793009	1.5936906	0.3752507	1.16E-05
CM	peroxidase	CCM_05131	69.85829	45.679759	56.577624	-0.5390286	-0.5223014	0.0622659	0.0697746
CM	peroxidase	CCM_06954	0.0301653	0.154259	0.4984327	--	--	--	--
CM	peroxidase	CCM_08032	340.42799	132.78213	58.012378	-1.2883053	-2.7729157	0.0011488	8.96E-13
CM	peroxidase	CCM_09023	50.441314	26.354717	74.408665	-0.7550447	0.2988918	0.5743873	0.7285287
CM	peroxiredoxin	CCM_03275	470.61466	286.21834	331.80157	-0.6491243	-0.6746015	0.7960139	0.7529745
CM	peroxiredoxin	CCM_03643	152.41056	158.28416	129.98039	0.1207334	-0.453521	0.7696878	0.1536079
CM	peroxiredoxin	CCM_06109	1.2068623	0.5370283	0.362008	--	--	--	--
CM	peroxiredoxin	CCM_08136	10.109028	18.009875	21.991136	0.8977934	0.9050585	0.0074473	0.0126056
CM	peroxiredoxin	CCM_08201	40.875512	34.835675	37.364523	-0.164636	-0.3566163	0.7467505	0.3838533
CM	superoxide dismutase	CCM_02198	0.3146463	0.5180077	0.4475487	--	--	--	--
CM	superoxide dismutase	CCM_03403	176.79003	161.36657	93.271485	-0.0581278	-1.1403422	0.962232	0.0199135
CM	superoxide dismutase	CCM_04979	284.31869	196.73599	193.13709	-0.6850687	-0.8828554	0.0537747	0.0058002
CM	superoxide dismutase	CCM_07115	1279.4084	923.88281	639.5979	-0.4002761	-1.2168259	0.4262396	0.0027304
CM	superoxide dismutase	CCM_08562	111.58514	236.46508	223.70622	1.1506152	0.7907271	2.165E-05	0.0252671
CM	superoxide dismutase	CCM_09430	12.042042	8.6270127	11.427859	-0.4249287	-0.2973752	0.2537461	0.4420954
CM	thioredoxin	CCM_00029	28.765161	75.702235	161.71778	1.4585156	2.2803179	4.59E-07	0.0001757
CM	thioredoxin	CCM_00285	0.4215973	0.3110107	0.261521	--	--	--	--
CM	thioredoxin	CCM_00331	41.187091	35.421649	50.305392	-0.1302797	0.0718915	0.7438172	0.8360884
CM	thioredoxin	CCM_00956	23.957037	13.887676	31.872764	-0.7199197	0.1986134	0.0732895	0.7081361
CM	thioredoxin	CCM_00965	38.080297	63.017821	54.927474	0.800272	0.3153928	0.0146685	0.4572592
CM	thioredoxin	CCM_01163	46.073924	125.3958	201.06105	1.5076353	1.908805	2.20E-09	2.70E-14
CM	thioredoxin	CCM_01377	68.685394	104.34696	111.41706	0.6787113	0.4898202	0.0221174	0.1004805
CM	thioredoxin	CCM_01863	11.666287	10.490626	20.213701	-0.0891268	0.570384	0.8306612	0.0546482

CM	thioredoxin	CCM_02074	0	0	0	--	--	--	--
CM	thioredoxin	CCM_03175	18.609202	5.6645337	6.300413	-1.6420766	-1.782069	2.59E-05	1.74E-05
CM	thioredoxin	CCM_03179	6.3823837	3.8702677	3.5602497	-0.6582345	-1.064416	0.2621139	0.0380755
CM	thioredoxin	CCM_03584	52.39135	52.211431	63.136822	0.0636794	0.0543033	0.9073806	0.9117603
CM	thioredoxin	CCM_04069	20.076206	19.934823	20.39205	0.0555019	-0.1978889	0.9077577	0.6830514
CM	thioredoxin	CCM_04323	73.891096	98.956695	100.2314	0.4929554	0.2258866	0.1897937	0.5768932
CM	thioredoxin	CCM_06167	41.4848	50.744244	48.16568	0.2091088	-0.0685374	0.6407669	0.8290733
CM	thioredoxin	CCM_06952	250.84643	334.48851	243.46001	0.4865795	-0.257986	0.2660965	0.5443022
CM	thioredoxin	CCM_07450	85.265078	82.795138	88.638486	0.1604531	0.0317735	0.7120233	0.966762
CM	**thioredoxin**	**CCM_07663**	**47.00457**	**122.0111**	**58.02748**	**1.443159**	**0.085397**	**6.94E-08**	**0.877416**
CM	thioredoxin	CCM_07753	40.801675	34.802757	29.756288	-0.1644066	-0.6814634	0.7029781	0.0203625
CM	thioredoxin	CCM_07812	132.00811	267.67021	215.52999	1.0856368	0.4998838	1.93E-05	0.4718828
CM	thioredoxin	CCM_07865	131.82911	181.61264	157.01213	0.3429019	-0.1462511	0.434821	0.7391035
CM	thioredoxin	CCM_08985	1.103672	2.3173843	18.577649	1.1205103	3.8234744	0.0698877	1.70E-16
CM	thioredoxin reductase	CCM_05420	41.236635	65.578687	103.05077	0.7448795	1.120726	0.0286209	0.0008096
CM	thioredoxin reductase	CCM_08835	531.71928	1223.0133	504.59275	1.2758225	-0.2908696	0.0028109	0.5485539
CC	catalase	CCOR_01363	1.622936	17.223579	39.072208	3.9351595	4.7706416	1.77E-30	1.29E-77
CC	catalase	CCOR_01696	24.88525	3.3405997	5.4021023	-2.315947	-1.9680475	1.27E-05	2.20E-05
CC	catalase	CCOR_03431	1.0303797	0.275401	0.5621567	--	--	--	--
CC	cytochrome oxidase	CCOR_00279	76.007657	72.128717	47.846969	0.5044047	-0.4381017	0.1267278	0.0886857
CC	cytochrome oxidase	CCOR_04587	30.135263	26.560813	17.354011	0.3882209	-0.5673165	0.2209684	0.0273141
CC	cytochrome oxidase	CCOR_07955	112.33708	26.462745	25.122914	-0.6907982	-0.6480757	0.0598718	0.0056215
CC	cytochrome oxidase	CCOR_07983	33.394573	52.958403	23.229332	1.2440066	-0.2987717	0.000104	0.3254717
CC	cytochrome oxidase	CCOR_08880	132.84956	234.74875	83.003289	1.3586312	-0.4270887	3.78E-06	0.0872834

CC	cytochrome oxidase	CCOR_09667	48.415593	54.742926	34.31292	0.7488669	-0.2664314	0.037235	0.4135784
CC	cytochrome oxidase	CCOR_09823	44.048633	48.546467	31.413628	0.7110295	-0.2585118	0.023318	0.3569038
CC	glutaredoxin	CCOR_03099	25.757844	40.008564	31.515982	1.2113584	0.5207534	0.0002459	0.059336
CC	glutaredoxin	CCOR_03142	2.2850677	29.422019	1.6525003	--	--	--	--
CC	glutaredoxin	CCOR_03236	197.42449	115.10513	131.21777	-0.2105279	-0.3602996	0.522059	0.1700275
CC	glutaredoxin	CCOR_05615	0.2167963	0.0506667	0.186363	--	--	--	--
CC	glutaredoxin	CCOR_06438	85.951752	84.845617	56.118898	0.5575651	-0.3851928	0.0865101	0.1483107
CC	glutathione peroxidase	CCOR_03861	35.347817	19.850444	12.306858	-0.2517488	-1.2886721	0.7997045	0.1573704
CC	glutathione peroxidase	CCOR_07175	125.20642	116.90995	93.268269	0.4678697	-0.1948404	0.1322362	0.4891165
CC	glutathione peroxidase	CCOR_08416	87.609033	37.061822	47.952955	-0.6753705	-0.5057567	0.0194917	0.0371667
CC	glutathione reductase	CCOR_08804	106.64463	95.105332	82.9842	0.4395465	-0.0430337	0.1374914	0.8941568
CC	NADPH oxidase	CCOR_00372	65.34957	99.794329	95.472463	1.1877575	0.7720924	7.12E-05	0.0015325
CC	NADPH oxidase	CCOR_09663	35.853354	19.122584	44.286608	-0.3378767	0.533652	0.2927889	0.0369146
CC	peroxidase	CCOR_01766	234.46347	121.95064	142.78956	-0.3680635	-0.4965668	0.3087115	0.1412044
CC	peroxidase	CCOR_01769	4.6001363	183.23077	53.947169	5.8791992	3.7789331	3.77E-82	8.18E-39
CC	peroxidase	CCOR_03182	32.908562	63.594838	11.902396	1.4477955	-1.0895281	0.0034195	0.3157874
CC	peroxidase	CCOR_03486	12.388627	6.8365137	9.8836543	-0.2977453	-0.0981039	0.646205	0.8212073
CC	peroxidase	CCOR_04082	0.779731	1.1009493	1.3282787	1.0640659	0.9958033	0.1305762	0.0459707
CC	peroxidase	CCOR_05122	2.8847687	0.1125213	0.766419	-3.9111517	-1.666845	2.46E-06	0.0009577
CC	peroxidase	CCOR_05190	1.8880567	4.9329427	3.582395	1.4566778	1.0179559	0.0021161	0.0019752
CC	peroxidase	CCOR_05341	812.9998	686.84481	607.42325	0.3228643	-0.1909385	0.4323465	0.6555372
CC	peroxidase	CCOR_06879	0	0.101904	0	--	--	--	--
CC	peroxidase	CCOR_07859	5.5857327	0.6434027	1.300852	-2.5429896	-1.8633338	0.000664	0.0008069
CC	peroxidase	CCOR_08207	0.708919	0.1100137	0.1342623	-1.831382	-2.1432913	0.0143784	7.73E-05

CC	peroxidase	CCOR_09102	1.118352	0.6288223	2.680359	-0.2343481	1.4879684	0.8574822	0.0006696
CC	peroxidase	CCOR_09270	1.9339853	0.9072407	0.7608367	-0.4711705	-1.1118663	0.5764668	0.0313098
CC	peroxidase	CCOR_09882	374.83654	373.05457	445.31162	0.569311	0.4704991	0.2361253	0.1924517
CC	peroxidase	CCOR_10391	3.184442	0.3714437	0.725664	-2.444343	-1.8778122	3.21E-05	1.36E-06
CC	peroxiredoxin	CCOR_00474	928.77057	6338.3701	3609.6353	3.3414261	2.1861942	8.71E-16	5.36E-11
CC	peroxiredoxin	CCOR_01029	26.480492	28.211313	28.154742	0.6620152	0.3150567	0.1220161	0.3355254
CC	peroxiredoxin	CCOR_04168	355.54518	255.08662	146.13298	0.0976867	-1.0504767	0.8644921	0.0098698
CC	peroxiredoxin	CCOR_04937	685.12968	362.77889	505.38828	-0.3655024	-0.2116715	0.3341688	0.5404279
CC	peroxiredoxin	CCOR_06320	140.80607	170.59956	115.45289	0.8535471	-0.0610225	0.0047634	0.8836013
CC	peroxiredoxin	CCOR_09228	207.01198	51.212077	36.886159	-1.4535141	-2.2583503	1.45E-07	6.81E-23
CC	peroxiredoxin	CCOR_09783	59.924177	47.82188	45.53199	0.2388879	-0.1689714	0.4802801	0.5639297
CC	superoxide dismutase	CCOR_00480	184.59172	220.19259	136.38434	0.8250208	-0.2055896	0.0061215	0.4595581
CC	superoxide dismutase	CCOR_02852	233.01026	233.25184	196.11758	0.5671345	-0.0218228	0.0614526	0.9567935
CC	superoxide dismutase	CCOR_03994	153.37919	211.24693	116.33378	1.0330261	-0.171397	0.0057248	0.6409467
CC	superoxide dismutase	CCOR_05552	1105.4823	183.45201	106.70381	-2.0065922	-3.1427076	8.30E-06	4.60E-12
CC	superoxide dismutase	CCOR_08621	97.6809	54.903696	46.114368	-0.5867845	-0.8005105	0.0404481	0.0009489
CC	superoxide dismutase	CCOR_08947	51.383157	101.75325	47.247012	1.5617172	0.105403	1.00E-07	0.7128555
CC	thioredoxin	CCOR_00771	52.836647	18.003222	24.763761	-0.9781628	-0.8627112	0.0040814	0.000916
CC	thioredoxin	CCOR_00933	69.222617	34.919967	71.453749	-0.4256832	0.2778817	0.2214575	0.4168551
CC	thioredoxin	CCOR_01459	17.188567	18.633195	17.984045	0.6218147	0.3002433	0.0500396	0.2954677
CC	thioredoxin	CCOR_01988	86.445869	136.34616	96.16155	1.234029	0.3833315	3.44E-05	0.1493972
CC	thioredoxin	CCOR_01996	0.0310873	0.0346647	0.003619	--	--	--	--
CC	thioredoxin	CCOR_02075	50.750759	57.601868	66.314826	0.7437934	0.6165678	0.0144097	0.0162158
CC	thioredoxin	CCOR_02607	12.832947	5.4293393	12.394046	-0.6799068	0.1804122	0.3042696	0.713789

CC	thioredoxin	CCOR_03022	96.390793	65.316186	80.570821	0.0029492	-0.0293823	0.9564308	0.9243884
CC	thioredoxin	CCOR_03181	240.13164	699.45746	428.25811	2.1158257	1.0625936	4.19E-10	0.0002125
CC	thioredoxin	CCOR_03253	0.017049	0.9984477	0.1128627	--	--	--	--
CC	thioredoxin	CCOR_03448	86.004842	41.658104	26.589013	-0.4618819	-1.4644768	0.1065915	7.20E-10
CC	thioredoxin	CCOR_03782	32.517525	22.05098	33.179535	-0.0003603	0.2585486	0.9324546	0.3434911
CC	thioredoxin	CCOR_03902	67.79143	80.784462	37.630978	0.8308406	-0.6185333	0.0132327	0.0219566
CC	thioredoxin	CCOR_04174	43.430613	30.488321	66.110133	0.0498824	0.8352594	0.8437525	0.0006945
CC	thioredoxin	CCOR_04539	2.771951	1.8939323	3.715752	0.0189328	0.6459537	0.9605548	0.1904369
CC	thioredoxin	CCOR_04983	244.49428	335.48774	414.18492	1.0081067	0.9964893	0.030968	0.2031691
CC	thioredoxin	CCOR_05700	98.88249	52.706402	54.209245	-0.3383336	-0.6404287	0.3036495	0.0084373
CC	thioredoxin	CCOR_05920	62.550526	52.774736	70.066185	0.3220237	0.3905943	0.2901793	0.1289542
CC	thioredoxin	CCOR_06273	125.01176	169.43906	90.401985	1.0125295	-0.2396108	0.000608	0.3720977
CC	thioredoxin	CCOR_06520	32.841773	15.89583	35.33272	-0.1614881	0.7395874	0.8642096	0.23318
CC	thioredoxin	CCOR_06974	13.437222	63.361745	3.3538347	2.7590023	-1.7881111	0.049844	0.6346069
CC	thioredoxin	CCOR_07126	20.988531	13.278144	16.845227	-0.0905805	-0.0897626	0.810911	0.7953674
CC	thioredoxin	CCOR_08668	5.9905963	2.354212	3.276695	-0.7506517	-0.631431	0.4461886	0.2342589
CC	thioredoxin	CCOR_08714	5.6385253	4.731016	5.9204073	0.3677206	0.3117478	0.5545912	0.4143475
CC	thioredoxin	CCOR_08813	71.944051	44.508643	55.201879	-0.1309928	-0.1566679	0.7743696	0.6652129
CC	thioredoxin	CCOR_09066	58.129758	49.949355	55.301061	0.3446732	0.1551225	0.2496094	0.576148
CC	thioredoxin	CCOR_09339	88.872846	96.690099	44.745024	0.6970563	-0.7612272	0.033093	0.0037071
CC	thioredoxin	CCOR_09546	101.40589	123.4783	81.312953	0.8505787	-0.1006386	0.0048155	0.7039157
CC	thioredoxin	CCOR_09552	105.91214	42.290137	72.698311	-0.7572237	-0.3175974	0.008608	0.250341
CC	thioredoxin	CCOR_09697	13.647689	10.415242	10.973985	0.1753871	-0.0872162	0.7419713	0.8557299
CC	thioredoxin	CCOR_09859	215.97132	377.56388	311.15658	1.3752901	0.7567926	8.81E-06	0.0061559

CC	thioredoxin	CCOR_10237	185.14627	39.609385	56.546349	-1.657543	-1.482764	4.93E-10	1.23E-10
CC	thioredoxin reductase	CCOR_01671	1.900472	2.8126887	2.889257	1.1529319	0.8364408	0.1287084	0.1057575
CC	thioredoxin reductase	CCOR_02356	77.18603	107.13228	63.874779	1.046485	-0.0461989	0.0003165	0.921359
CC	thioredoxin reductase	CCOR_07370	0.3349257	0	1.12753	--	1.9555502	--	0.0001418
CC	thioredoxin reductase	CCOR_07915	503.2881	1271.4309	1188.5702	1.8983395	1.469906	1.08E-07	6.31E-06
CC	thioredoxin reductase	CCOR_09265	54.030731	5.9896067	71.362676	-2.5978801	0.6290787	1.43E-16	0.0086517
